# Pd-, Cu-, and Ni-Catalyzed Reactions: A Comprehensive Review of the Efficient Approaches towards the Synthesis of Antibacterial Molecules

**DOI:** 10.3390/ph17101370

**Published:** 2024-10-15

**Authors:** Almeera Zia, Shehla Khalid, Nasir Rasool, Nayab Mohsin, Muhammad Imran, Sebastian Ionut Toma, Catalin Misarca, Oana Andreescu

**Affiliations:** 1 Department of Chemistry, Government College University, Faisalabad 38000, Pakistan; almeerazia817@gmail.com (A.Z.); shehlakhalid47@gmail.com (S.K.); nayabmohsin7869@gmail.com (N.M.); 2 Research Center for Advanced Materials Science (RCAMS), King Khalid University, P.O. Box 9004, Abha 61413, Saudi Arabia; imranchemist@gmail.com; 3 Chemistry Department, Faculty of Science, King Khalid University, P.O. Box 9004, Abha 61413, Saudi Arabia; 4 Faculty of Medicine, Transilvania University of Brasov, 500036 Brasov, Romania; cmisarca@gmail.com (C.M.); oana.andreescu@unitbv.ro (O.A.)

**Keywords:** Pd, Ni and Cu catalyst, cross coupling reactions, bioactive molecules, antibacterial molecules

## Abstract

A strong synthetic tool for many naturally occurring chemicals, polymers, and pharmaceutical substances is transition metal-catalyzed synthesis. A serious concern to human health is the emergence of bacterial resistance to a broad spectrum of antibacterial medications. The synthesis of chemical molecules that are potential antibacterial candidates is underway. The main contributions to medicine are found to be effective in transition metal catalysis and heterocyclic chemistry. This review underlines the use of heterocycles and certain effective transition metals (Pd, Cu, and Ni) as catalysts in chemical methods for the synthesis of antibacterial compounds. Pharmaceutical chemists might opt for clinical exploration of these techniques due to their potential.

## 1. Introduction

Over time, bacterial illnesses have grown to be a serious hazard, with elevated rates of death and morbidity globally [1]. Antimicrobial agents combat pathogenic bacteria by either eliminating or drastically lowering their metabolic activity. When antibiotics were initially developed in the 1900s, people believed that the battle against microbes had been won. However, it was quickly found that the microbes may become resistant to any medication that was being offered. Antibiotics that we currently have in stock are rapidly losing their effectiveness, and there is no indication that they will be sufficiently restocked soon [2,3]. An increasing number of bacterial infections resist conventional treatment and are challenging, if not untreatable, to cure. Globally, resistance to several antibiotics is becoming more widespread, and there are an increasing number of reports of treatment failures and rising expenses, particularly in the hospital setting [4,5]. Based on their ability to withstand these antimicrobial drugs, bacteria can be classified into two categories: drug-resistant bacteria and multiple drug-resistant bacteria. Bacteria that have developed a resistance to specific medications are known as multidrug-resistant, meaning that the bacteria can no longer be controlled or killed by the antibiotics. More importantly, the emergence of these multi-drug-resistant bacteria is one of the biggest hazards to public health and the whole economy. The main causes of multiple drug-resistant bacteria are overexpression of the efflux pump, bacterial gene mutation, and bacterial adaptability to antibiotics [6]. Most of the world’s countries have abused antibacterial agents, which complicates this issue. Between 1983 and 2012, the FDA approved fewer new systemic antibacterial medications, while the phenomenon of bacterial resistance was becoming even worse [2]. Antibiotic resistance is a global health concern that poses a danger to the effectiveness of traditional antibiotics against common bacterial diseases. In 2022, the WHO released the Global Antimicrobial Resistance and Use Surveillance System (GLASS) report, which reveals resistance rates among common bacterial infections. In 2019, the Centers for Disease Control and Prevention published an overview of risks posed by antibiotic resistance in the US, highlighting the “potentially catastrophic consequences of inaction”. To synthesize novel antibacterial drugs in response, new mechanisms are incredibly needed [7].

Small drug molecules have historically been the most often utilized medicines in antibacterial therapy [8,9]. The antibacterial properties of heterocyclic organic compounds are mostly mediated by metal ions [10,11]. Some natural products, including alkaloids such as morphine, vinblastine, and reserpine, and antibiotics such as cephalosporin and penicillin, include a heterocyclic moiety. Aromatic heterocyclic derivatives, such as β-lactam derivatives, are a key component of the chemical structure of antibiotics. These demonstrate the significant role that heterocyclic compounds play in the formation of antibacterial drugs when they combine with other molecules. Additional methods such as the azide-alkyne reaction and click reaction are also employed in the synthesis of antibiotics. Therefore, chemists must combine heterocycles with other small compounds and copper-catalyzed reactions, such as the azide-alkyne reaction and click reaction, to develop new antibacterial agents. FDA-approved different antibacterial drugs are synthesized by using different transition metals as catalysts [12].

Thus, it is necessary to alter the medications that are now on the market to develop brand-new antibiotics that are impervious to germs. It is now clear that there is an immediate requirement for new antibacterial medications that do not show resistance to currently used antibiotics, as well as for increased efforts to promote the prudent and responsible use of antibiotics [13,14] and improved infection control practices. The ability of organic synthesis to synthesize compounds that are primarily carbon-based, including those found in living things, was a key factor in this revolution. With time, pharmacists and chemists produced new medications by utilizing different approaches and techniques; however, these medications are insufficient. More precisely, the best chance for the quick discovery and development of novel antibiotics in the near future is for us to provide workable, diversifiable, fully synthetic pathways to antibiotic scaffolds that are not now accessible [15].

Drugs synthesized via metal catalysis are valsartan [16], losartan [17], irbesartan [18], atazanavir [19], ruxolitinib derivatives [20], etoricoxib [21], lapatinib [22], etc. Some marketed antibacterial drugs have also been synthesized by using transition metal catalysis, e.g., penicillin [23], cephalosporin [24], linezolid [25], vancomycin [26], and tetracycline derivatives [27]. Microwave Ullmann reaction conditions were used by Sun and colleagues (2012) to synthesize macrocyclic diaryl ethers, including diarylheptanoids [28]. Significant antibacterial activity was shown by the 15-membered p-fluorophenethylamine derivative and the geranylamine derivative, which were produced by the Ullmann process [29,30]. This review highlights the chemical approaches that might be used in the future to synthesize marketed drugs against antibacterial infections. This review provides insight into research areas of heterocyclic chemistry, transition metal catalysis, and medicinal chemistry. Pd, Cu, and Ni are widely used in synthetic chemistry because of their efficiency.

In this study, we compiled research from the previous five years that discussed the role of transition metals as catalysts in the synthesis of crucial small organic compounds and antibacterial agents. The research data have been organized specifically for efficient Pd, Cu, and Ni-catalyzed antibacterial molecule synthesis. Subdivision according to cross-coupling and named reactions is also being completed. The WHO has designated certain bacterial infections as “critical priorities”, and the rate of death from these diseases has increased in humans as well as in plants and animals. As a result, rising rates of antibiotic resistance are widely regarded as an impending problem. Studying all antibacterial compounds made with transition metal as a catalyst is therefore imperative to combat bacterial resistance to several drugs, reduce the mortality rate in living things, and combat multidrug-resistant germs.

## 2. Palladium-Catalyzed Synthesis

Currently, one of the most adaptable and practical methods for performing organic synthesis in both academic labs and industrial production facilities is cross-couplings and related reactions. For all these reactions to continue at a synthetically useful pace, a transition metal catalyst is typically required. Transition metal complexes find extensive uses in photochemistry, biological systems, material production, and catalysis.

Palladium is a key component in cross-coupling reactions, even though other metal centers might theoretically catalyze the different stages of these processes. Cross-coupling reactions in which carbon-carbon bonds are formed by using palladium as a catalyst include those of Suzuki, Sonogashira, Heck, Negishi, Stille, Corriu-Kumada, Ullmann, Tsuji-Trost, and Hiyama. Pd salts or complexes, either prepared or generated in situ upon the addition of a ligand, are popular Pd sources for cross-couplings and related processes. Palladium (II) species are typically selected as starting materials due to their greater stability. Following their in-situ reduction, these substances become palladium (0) species, which then add to the catalytic cycle. It is interesting to note that Pd metal has been used as a catalyst for these reactions for almost as long as the reactions themselves: the Heck reaction was successfully used for the first time in the early 1970s [31,32], and Julia and colleagues’ revolutionary research was the first to apply supported Pd metal [33,34]. Up to now, Pd metal-based catalytic systems for these reactions have been continually developed, yielding catalysts with good activity, selectivity, and durability.

### 2.1. Suzuki Cross-Coupling

**Scheme 1:**
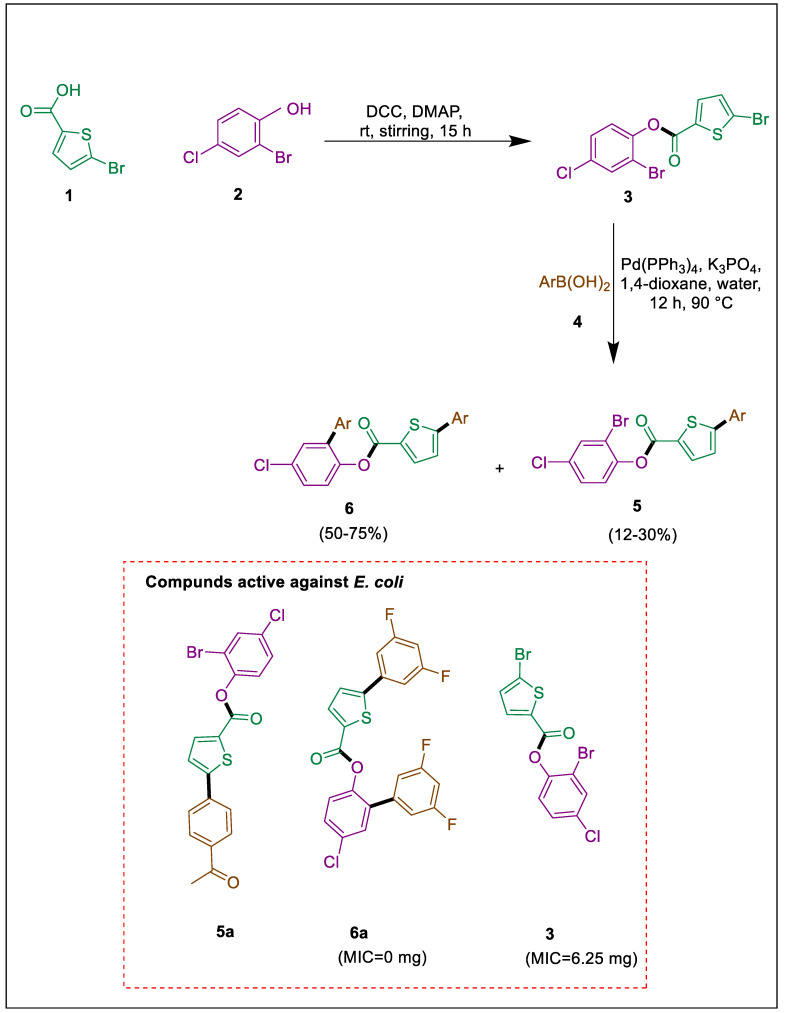
Synthesis of thiophene carboxylates by using Suzuki-Miyaura cross-coupling.

Suzuki cross-coupling is a significant synthetic reaction utilized in laboratories and industries for combinatorial and synthetic objectives [35]. Palladium-catalyzed Suzuki-Miyaura coupling reaction, was employed by Mujahid et al. to produce thiophene carboxylate derivatives of **5** and **6** with moderate to acceptable yields. In contrast, compound **3** was synthesized in the presence of DCC and DMAP via Steglich esterification of carboxylic acid **1** with 2-bromo-4-chlorophenol **2**. Compound **6a** exhibited the highest potential value, possessing the strongest binding affinities and the largest potential utility. It was demonstrated as an antibacterial agent against *E. coli* with a minimum inhibitory concentration (MIC) of 50 mg/mL. Antibacterial effectiveness in vitro was assessed for the active lead molecules; compounds **5a**, **6a**, and **3** exhibited activities against bacteria (Scheme 1) [36].

**Scheme 2:**
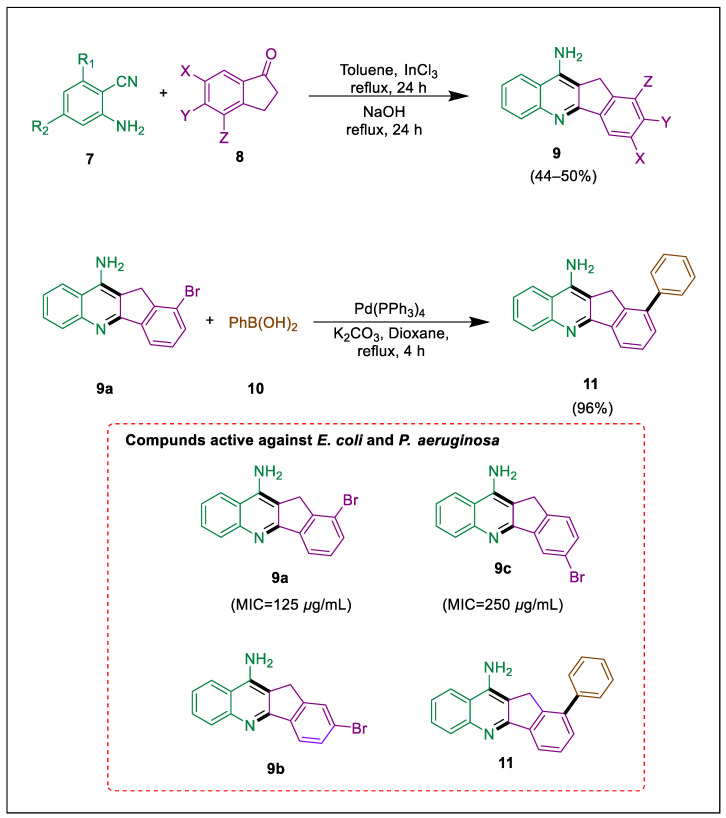
Synthesis of indenoquinoline amine by using Suzuki-Miyaura cross-coupling.

The number of antibiotic-resistant microbes has increased due to the recent overuse of medications [37]. All these advancements challenge scientific research regarding the synthesis of novel antimicrobial and anticancer agents. The noteworthy inhibitory effects of bromoindanol[1,2-*b*]quinolineamine compounds motivate us to examine their potential anticancer properties against various cancer cells and demonstrate their ability to kill bacteria. In their attempt to synthesize amine derivatives of indenol[1,2-*b*], Aydin et al. employed the Friedlander reaction. With MIC values ranging from 15.62–250 μg/mL, the monosubstituted indenol[1,2-*b*]quinolines (**9** and **11**) exhibit significantly enhanced antiproliferative and antimicrobial activity, according to the present investigation. The compound **11** (96% yield) was produced via Suzuki-Miyaura cross-coupling and possesses potent anticancer and antibacterial properties. Several bromo indan-1-ones **8** and 2-aminobenzonitrile **7** undergo a cyclodehydration reaction under toluene reflux, with Lewis’s acid serving as a catalyst and bromo indan-1-ones **8** treated with 2-amino-3,5-dibromobenzonitrile **7** to produce monobromo compound **9**. Subsequently, one equivalent of phenylboronic acid **10** was coupled with compound **9a** to produce 1-phenyl-5-indenol[1,2-*b*]quinoline amines **11** by using a Suzuki-Miyaura coupling reaction. The growth of all bacteria that are gram-positive was effectively halted by derivatives **9** and **11**. At the same time, moderate antibacterial effects of compound **9a** against *E. coli* and *P. aeruginosa* compound **9c** against only *E. coli* were observed (Scheme 2) [38].

**Scheme 3:**
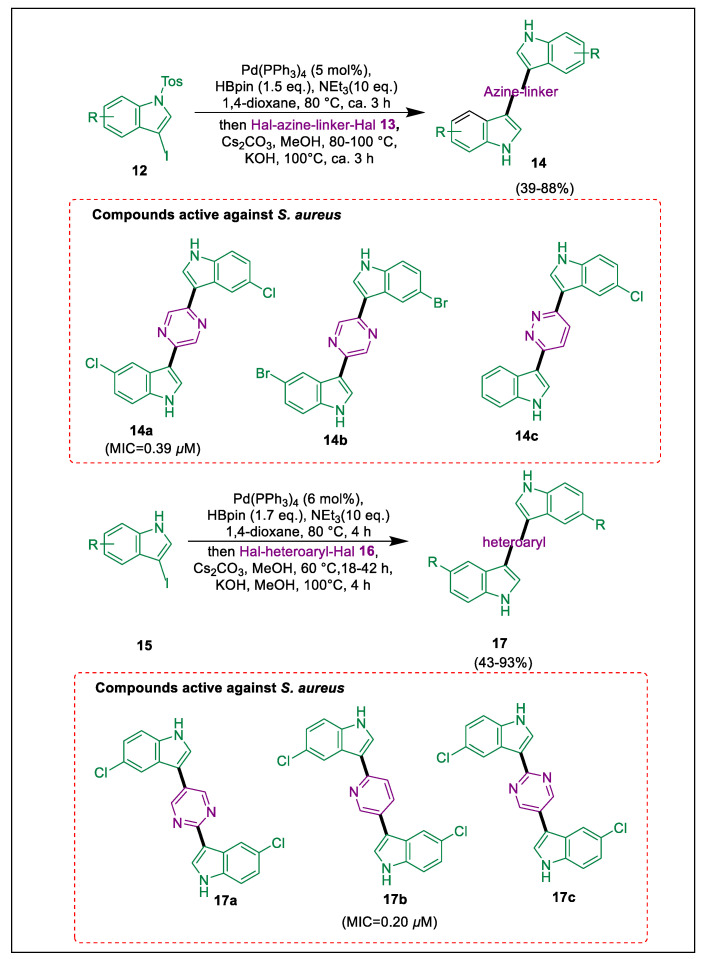
Synthesis of bisindoles derivatives by using Masuda borylation Suzuki coupling.

Types that are extraordinarily resistant to drugs, for which virtually no antibiotic is effective. *Staphylococcus aureus* is a bacterium that can cause disease [39]. The increasing resistance of *S. aureus* to antibacterial agents poses a significant clinical challenge in treating such infections. Natural bindles exhibit cytostatic and antifungal properties but no antibacterial activity by synthesizing natural compounds into pharmacophores, which have enhanced antibacterial activity compared to their parent molecules [40]. Rehberg et al. used the Masuda borylation-Suzuki coupling pathway to produce a library comprised of natural product-derived and artificial (di)azine-bridged bisindoles because unmodified bisindoles had no antibacterial action. 5,5′-chloro derivatives have remarkably significant action against other gram-positive bacteria as well as methicillin-resistant *S. aureus* (MRSA). Compounds **17a** and **17b** demonstrated significant in vivo effectiveness against MRSA in a wound infection test on mice. Natural Bisnode alkaloids, exemplified by Hyrtinadine A and Alocasin A, offer chemical scaffolds for drug development with considerable promise due to their recognized versatility in bioactivity. Desired product bisindoles **14** or **17** were obtained in high yield through the Masuda borylation of indoles **12** or **15** in dry 1,4-dioxane in an argon environment at 80–100 °C with water-free triethylamine and base cesium carbonate as carbonate/methanol mixtures for Suzuki coupling processes at elevated temperatures, followed by the addition of dithiatopazines or heteroaryl halides utilized in a mixture containing water as a co-solvent during the Suzuki coupling process. Compounds **17a**, **17b**, and **17c** demonstrated broad-spectrum antibacterial activity against every gram-positive nosocomial pathogen examined. The bactericidal activity of compounds **14a**, **14b**, **17j**, **17b**, **17c**, and **14c** was significant (Scheme 3) [41].

**Scheme 4:**
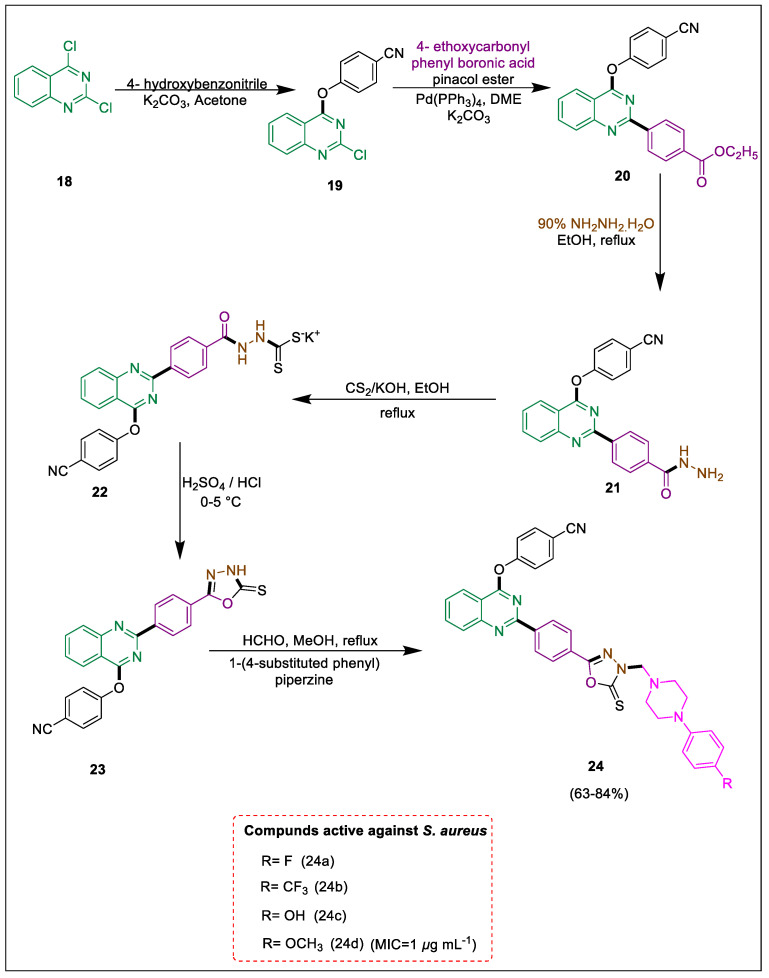
Synthesis of Mannich bases by using Suzuki cross-coupling.

Exploring novel heterocycles that exhibit efficacy against numerous biological indicators continues to be a captivating technological pursuit. It has been documented that compounds comprising a quinazoline moiety reveal diverse natural and therapeutic characteristics. 1,3,4-Oxadiazole as a possible physical component. Phenylpiperazine bases that have been *N*-substituted exhibit various pharmacological effects [42,43,44,45,46]. A series of piperazine-fused and oxadiazole-fused quinazoline derivatives were produced by Patel et al. After an effective Suzuki cross-coupling on the quinazoline ring, 1,3,4-oxadiazole intermediates are generated as part of the synthetic protocol. In methanol, these oxadiazoles are treated with piperazine bases, while formalin is present to produce the final *N*-Mannich compounds. A considerable proportion of these compounds exhibit formidable antibacterial properties when tested against diverse strains of gram-positive bacteria. The synthesis of benzonitrile **19** involves the reaction of dichlorquinazoline **18** with 4-hydroxybenzonitrile under basic conditions. This molecule **19** then undergoes a Suzuki coupling with the phenyl ring of the boronic acid pinacol ester, resulting in the formation of quinazoline benzoate **20**. Subsequently, hydrazide derivative **21** was obtained by treating the compound **20** with hydrazine hydrate in ethanol. Hydrazinecarbodithioate salt **22** was produced by cyclizing analogue **21** with carbon disulfide in ethanolic KOH. Subsequently, sulfuric acid and HCl were utilized to convert it into the comparable 1,3,4-oxadiazole derivatives **23,** which were reacted with various piperazines in the presence of formalin in methanol to obtain Mannich bases **24**. In contrast, the quinazoline derivative **24b** exhibited more encouraging efficacy against *S. aureus*. Furthermore, the activity of **24a**, **24c**, and **24d** against *S. aureus* was observed to be half-fold lower than that of **24b**. Notably, the methoxy piperazine-fused derivative **24d** exhibited activity against *S. aureus* as well. It was discovered that **24b**, **24c**, and **24d** were effective against quinolone-resistant *S. aureus* (Scheme 4) [47].

**Scheme 5:**
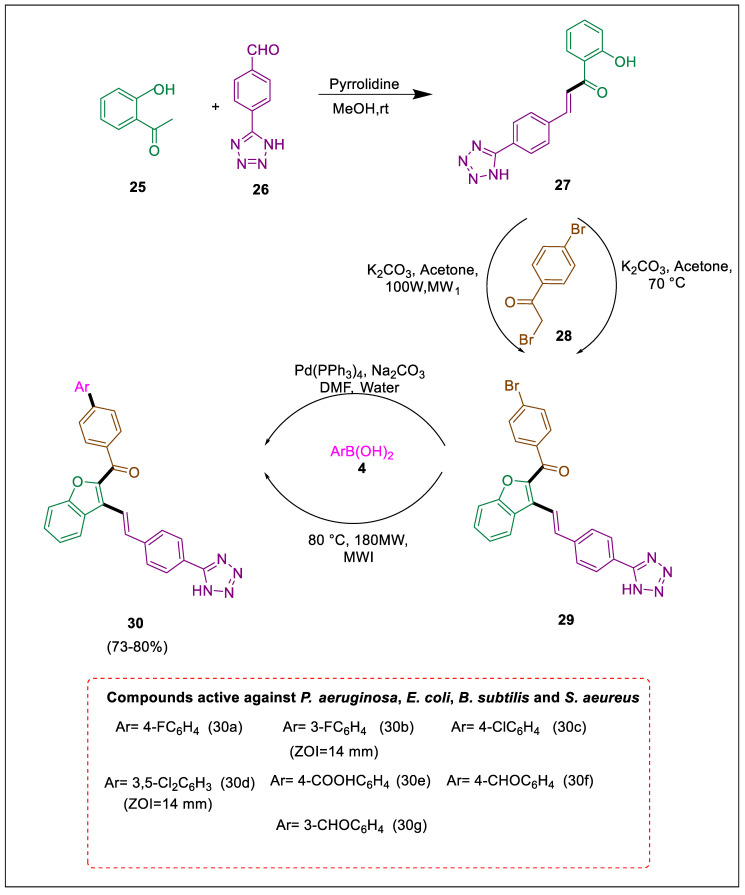
Synthesis of tetrazole derivatives by using Suzuki cross-coupling.

Tetrazole is an essential structure of numerous drug molecules [48,49,50,51,52,53,54]. For the manufacturing of biphenyls and polyaryls, a Suzuki cross-coupling reaction catalyzed by palladium is utilized due to the importance of aryl-aryl bond formation in organic synthesis [55,56,57]. In organic synthesis, using microwave irradiation [58] is advantageous due to its eco-friendliness and increased product yield, and the solvent used for the reaction is water. An eco-friendly approach was devised by Ashok et al. to produce substituted methanone scaffolds. This was accomplished by utilizing a Suzuki interaction reaction in an aqueous solution, employing conventional heating methods and microwaves. In vitro, the evaluation of synthesized frameworks for antifungal and antibacterial properties was performed. Using microwave irradiation increases the reaction rate, decreases by-products, and uses water as a solvent. 1-(*o*-tolyl)ethan-1-one **25** reacted with tetrazole benzaldehyde **26** to form compound **27**, which underwent a reaction with various substituted phenacyl bromides **28** used solvent dry acetone and anhydrous K_2_CO_3_ as a base to intermediate **29**, which was subsequently subjected to treatment with replaced arylboronic acids **4** in the presence of a Pd catalyst and Na_2_CO_3_ to yield tetrazole derivatives **30**. Compounds **30a**, **30b**, **30c**, and **30d** demonstrated the most significant zone of inhibition and superior activity against all bacterial organisms. The compounds **30e**, **30f**, and **30g** exhibit a moderate zone of prevention against bacterial strains but demonstrate limited activity (Scheme 5) [49].

**Scheme 6:**
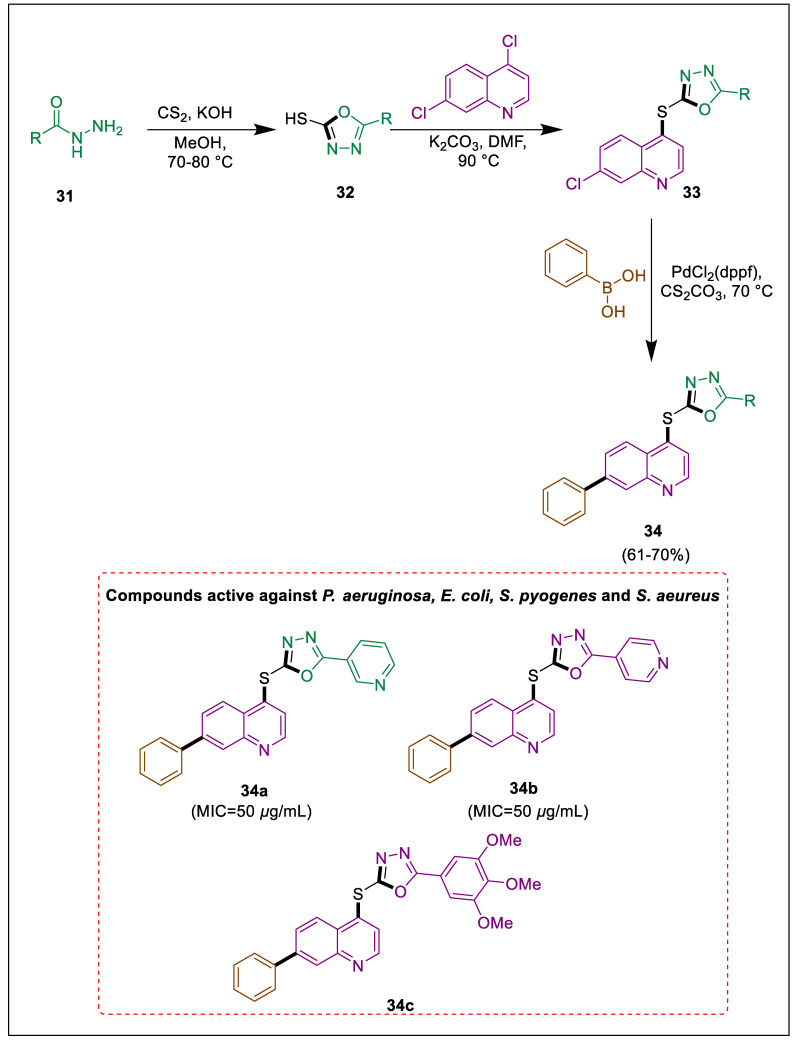
Synthesis of oxadiazole derivatives by using Suzuki cross-coupling.

It has been noticed that products of various heterocycles, which include quinoline and 1,3,4-oxadiazole, contain an extensive array of biologically efficacious molecules [59,60,61,62,63]. Both quinoline and oxadiazoles are widely recognized heterocycles that exhibit a substantial biochemical profile. The synthesis, evaluation, and antibacterial and antifungal effects of derivatives based on quinoline and oxadiazole are detailed in this article. Ten novel oxadiazole derivatives were formed by Thummar and Bhatt utilizing 4,7-dichloroquinoline in sequential reactions with various moieties, including phenylboronic acid and 1,3,4-oxadiazole, which produced the desired target with an exceptional yield. The antibacterial activity of the synthesized substances against gram-positive and gram-negative microorganisms was assessed in vitro. Oxadiazole derivatives were synthesized via a Pd-catalyzed Suzuki coupling reaction and a nucleophilic substitution reaction. Arylhydrazide **31** and CS_2_ in KOH form 1,3,4-oxadizole derivatives **32**, which were treated with 4,7-dichloroquinoline to afford a nucleophilic substitution product on the fourth position of 4,7-dichloroquinoline **33**. The desired oxadiazole derivatives **34** resulted from the palladium-catalyzed reaction of the -Cl remaining at position 7 of **33** with phenylboronic acid in an N_2_ atmosphere. Compounds **34** were assessed for antimicrobial capacity at 50 to 500 µg/mL concentrations. Compounds **34a**, **34b**, and **34c** exhibited MIC values of 50, 50, and 70 µg/mL, respectively (Scheme 6) [64].

**Scheme 7:**
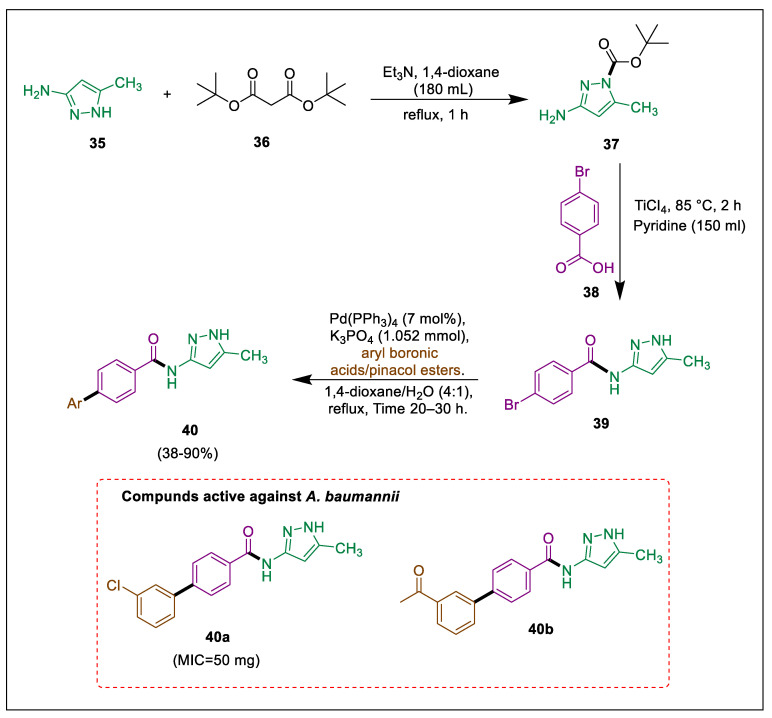
Synthesis of pyrazole amide derivatives by using metal catalysts through Suzuki cross-coupling.

The requirement for novel antimicrobial pharmaceuticals persists due to the diminishing efficacy of antibiotics that are presently accessible. With numerous biological functions, the pyrazole skeleton is a prominent structure in the pharmaceutical sector [65,66,67]. The initial identification of New-Delhi metallo-*b*-lactamase (NDM-1) occurred in 2009 in clinical isolates of *E. coli* and *K. pneumoniae* obtained from a patient in Sweden [68]. Ahmad et al. conducted a direct amination of protected amine **37** to produce benzamide **39**. The pyrazole amide derivatives of interest **40** were formed through a Suzuki coupling of the intermediate molecule **39** with various boronic acids catalyzed by palladium. The antibacterial activity of newly synthesized analogues **40** against NDM-1-positive *A. baumannii* and *K. pneumoniae* was assessed. Protection of amine **35** by di-*tert*-butyl tricarbonate **36** to synthesize carboxylate **37** and its one pot condensation with benzoic acid **38** in pyridine to form intermediate **39**, which undergo Suzuki coupling with different boronic acids to obtain pyrazole hybrids **40**. Compounds **40a** and **40b** exhibited the most significant zone of suppression towards NDM-positive *A. baumannii* compared to other compounds (Scheme 7) [69].

**Scheme 8:**
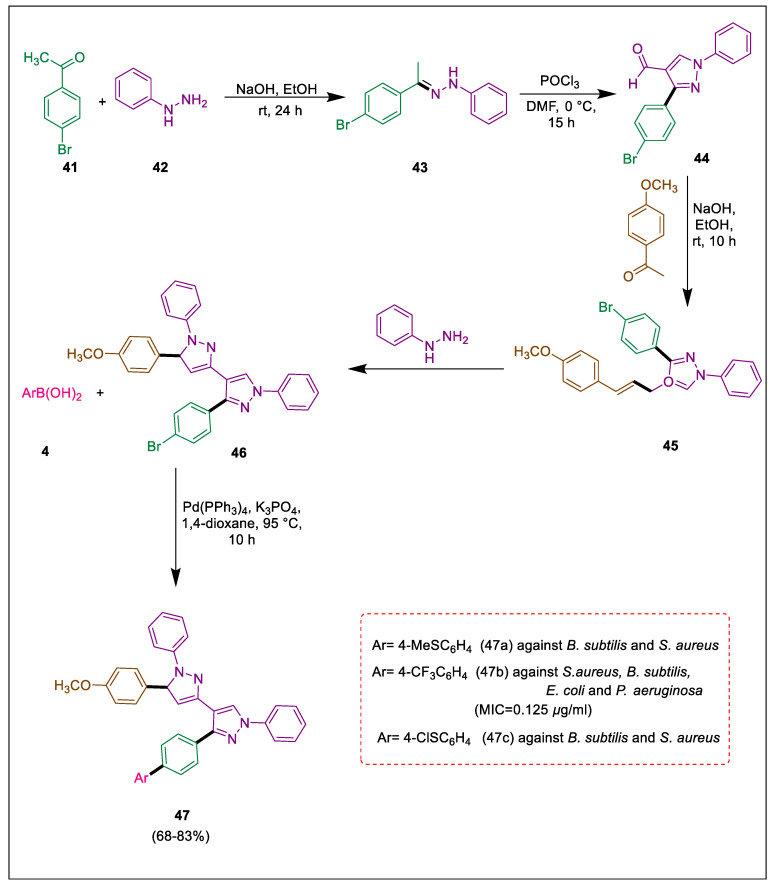
Synthesis of bispyrazole derivatives by using Suzuki cross-coupling reaction.

Globally, antibiotic-resistant infections are regarded as the primary cause of mortality due to antibacterial resistance. Heterocycles with nitrogen have numerous biological and chemical applications; for instance, pyrazole synthesized numerous biologically active compounds. Furthermore, natural compounds incorporating the pyrazole moiety exhibit highly potent physical properties [70,71,72,73]. In their study, Salman et al. produced an innovative sequence of bispyrazole derivatives with yields ranging from 68 to 83%, which can be classified as moderate to outstanding. The synthesized compounds were screened against gram-positive and gram-negative bacteria. Each newly synthesized compound demonstrated antibacterial activity ranging from excellent to moderate. Compound **47d** significantly inhibited the growth of all microorganisms that were tested. Using Suzuki coupling, bispyrazole derivatives are produced with a high yield [74]. Compounds bromophenylethane-1-one **41** and phenyl hydrazine **42** react to form compound **43**, which forms compound 4-(4-bromophenyl)-1-phenyl-1*H*-pyrazole-4-carbaldehyde **44** in the presence of DMF and POCl_3_. Compound **44** then reacts with *p*-methoxyacetophenone using NaOH and ethanol, followed by a dehydration reaction to form oxadiazole derivative **45**, which forms bispyrazole **46** in ethanol with some drops of glacial acetic acid under reflux [75]. Suzuki cross-coupling reaction [76,77,78,79,80] between bispyrazole **46** and various arylboronic acids **4** using [Pd(PPh_3_)], K_3_PO_4_, and dioxane at a temperature of 95 °C for 10 h for the formation of bispyrazole derivatives **47**. In vitro evaluation was conducted to determine the antimicrobial potency of target compounds **47**; compound **47b** demonstrated activity against all bacterial isolates. Compounds **47a** and **47c** exhibited significant inhibitory activity when tested towards *B. subtilis* and *S. aureus* (Scheme 8) [74].

**Scheme 9:**
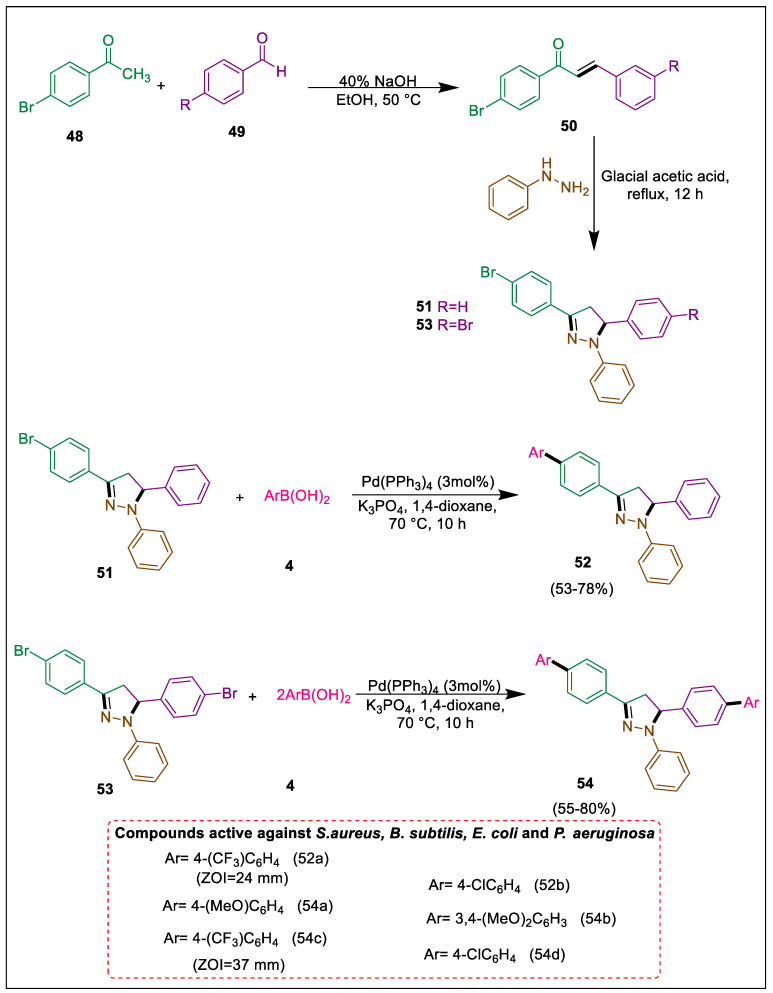
Synthesis of pyrazoline derivatives by using Suzuki cross-coupling reaction.

Natural goods often contain nitrogen-containing heterocyclic compounds, such as the pyrazoline scaffold, which have demonstrated exceptional utility in medicinal chemistry [81]. Substances comprising a 2-pyrazoline moiety exhibit biological activity [82,83,84,85]. Preliminary antibacterial activity of 2-pyrazoline derivatives was determined, which were produced via arylation, by using Suzuki-Miyaura reactions. In their study, Karim et al. utilized one equivalent of arylboronic acid in a Suzuki-Miyaura reaction with **51** to produce 3-(biphenyl)-1,5-diphenylpyrazoline. In contrast, two equivalents of aryl boronic acids with **53** via Suzuki-Miyaura reactions formed 3,5-bis(biphenyl)-1-phenylpyrazoline. Mono-coupling compound **52** demonstrated negligible antibacterial activity in contrast to the di-coupling compound **54**, which exhibited antibiotic activity against all bacterial strains. *p*-Bromoacetophenone **48** is condensed with benzaldehyde **49** in the presence of ethanol and NaOH to get substituted chalcone **50**. In the presence of glacial acetic acid, this compound interacts with phenyl hydrazine to generate derivatives of pyrazoline (**51**, **53**). In the presence of Pd catalyst, dioxane, and K_3_PO_4_, the SM reaction between **51** and arylboronic acids **4** results in pyrazoline derivative **52**. Suzuki-Miyaura reaction between **53** with arylboronic acid **4** formed 3,5-bis(biphenyl)-1-phenylpyrazoline **54** in the presence of Pd catalyst, K_2_CO_3_, 1,4-dioxane, at 100 °C temperature. Di-coupling compounds **54** demonstrated high bioactivity against four bacteria in comparison to trimethoprim, while mono-coupling compounds **52** showed slight activity against both gram-positive and negative bacteria, of which **52a**,**b**, and **54a**–**d** were more potent and showed antibacterial activity (Scheme 9) [86].

**Scheme 10:**
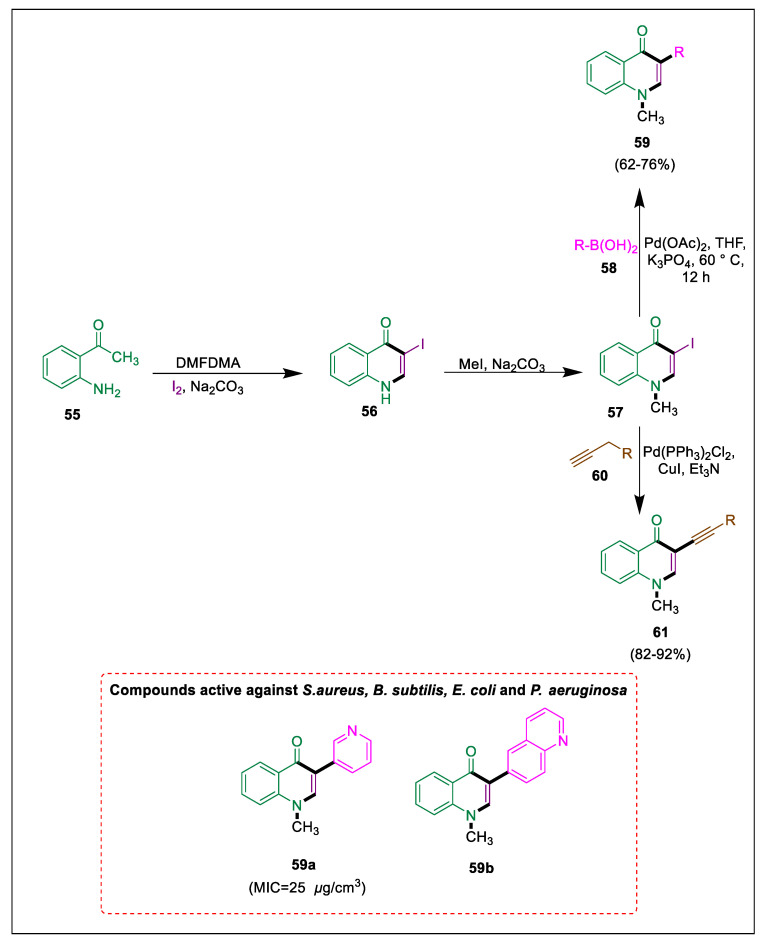
Synthesis of quinoline and aza-isoflavone derivatives by using Suzuki-Miyuara coupling.

It is known that quinolone scaffolds inhibit bacterial proliferation. An efficient artificial pathway for the synthesis of *E*-styryl quinolones [87,88] was documented by Silva et al. [89]. By using the slow-release method of the Suzuki-Miyuara and Sonogashira reactions, novel 3-substituted-4-quinolones were synthesized. Mohamed et al. performed a Suzuki coupling reaction in the presence of Pd as a catalyst by using *N*-methyl-quinolone with boronic acids to obtain aza-isoflavone derivatives. All produced compounds were tested for their antibacterial properties against a variety of bacterial strains. Commercially available 2-aminoacetophenone **55** is cyclized using dimethylformamide dimethyl acetal (DMFDMA) at 95 °C for three hours by using toluene as solvent and iodine in the presence of Na_2_CO_3_ to yield 3-iodoquinolone **56**. Compound **57** is then obtained by methylation, which undergoes SMC using the slow-release methodology with commercially available boronic acids **58** to form corresponding azaisoflavone analogues **59**. By reacting terminal alkynes **60** with iodoquinolone, a set of quinolone derivatives **61** were produced. An in vitro antibacterial assessment of each quinoline and aza isoflavone derivative (**59** and **61**) was conducted compared to ciprofloxacin, norfloxacin, and ampicillin, indicating that compounds **59a** and **59b** are the most active antibacterial agents (Scheme 10) [90].

**Scheme 11:**
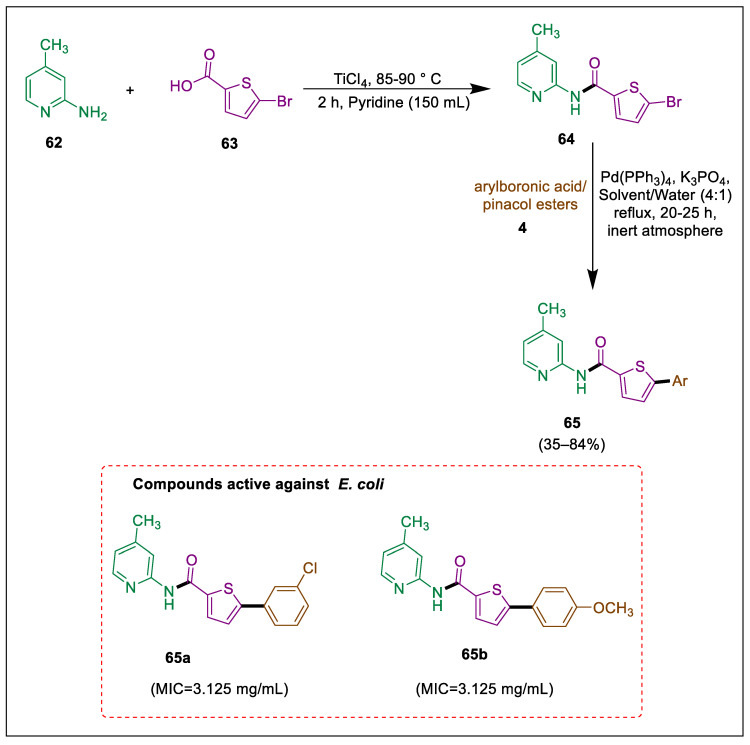
Synthesis of carboxamide derivatives by using Suzuki-Miyuara coupling.

Infections that are resistant to antibiotics are becoming more frequent and severe globally. Infections caused by extended-spectrum β-lactamase (ESBL) are classified as “High-Priority Pathogens” by the WHO [91]. Specifically, ESBL-producing *Escherichia coli* infections require treatment. Carboxamide serves as an essential scaffold due to its antibacterial attributes. Thiophene-carboxamides **65** were formed by Ahmad et al., and activity against ESBL-producing *E. coli* ST131 strains was evaluated. Compounds **65a** and **65b** are more potent inhibitors against the selected target enzymes (6N9K and 7BDS) of β-lactamase *E. coli* in comparison to the remaining carboxamide analogues. Compound **64** was formed by the reaction of carboxylic acid **62** with methylpyridine **63** in the presence of TiCl_4_ and pyridine. It then goes through a Suzuki-Miyaura reaction with different arylboronic acids **4**, using a commercially available palladium-tetrakis catalyst, resulting in thiophene-carboxamides **65**. A newly synthesized compound **65** was evaluated against ESBL-producing *E. coli* at five concentrations; compounds **65a** and **65b** exhibited the most significant zone of inhibition (13 ± 2 and 15 ± 2, respectively) in comparison to the other compounds that also demonstrated good activity (Scheme 11) [92].

**Scheme 12:**
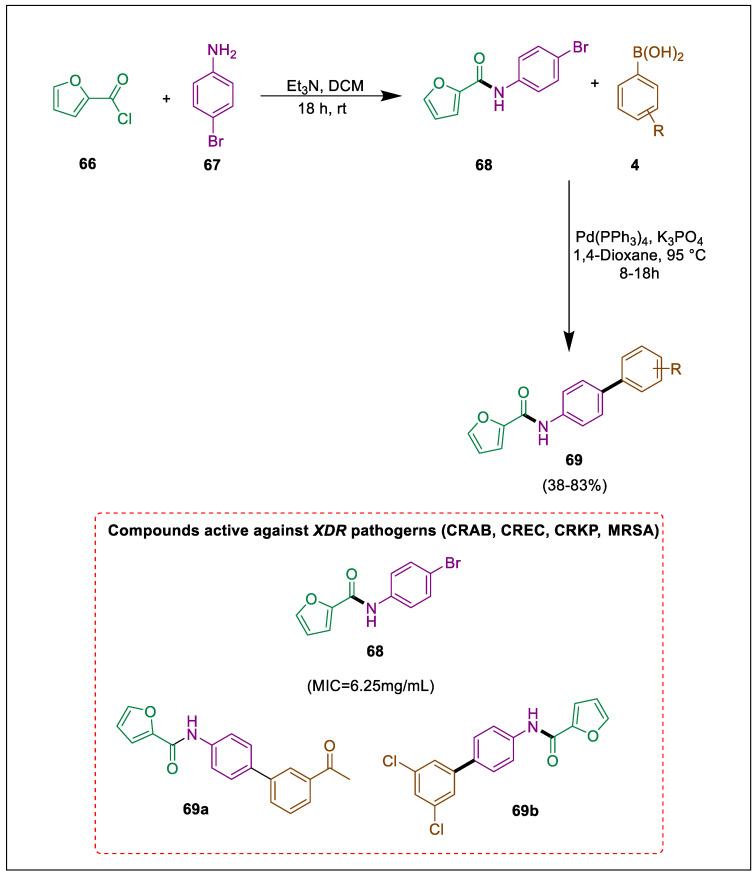
Synthesis of carboxamide derivatives by using Suzuki-Miyuara coupling.

Around the world, public healthcare systems are seriously threatened by the emergence of extensively drug-resistant (XDR) microorganisms [93]. The WHO listed infections that are resistant to carbapenem on its “Critical Pathogens List”. Numerous natural products, synthetic precursors, and pharmaceuticals contain oxygen-heterocycles [94,95,96,97]. Antibacterial properties may be exhibited by carboxyamide, an essential scaffold. By reacting furan derivative **66** with aniline **67** in the presence of Et_3_N, Siddiqa et al. synthesized carboxamide derivatives **68**. Carboxamide analogues **69** were obtained by arylating the carboxamide **68** using Pd as a catalyst and K_3_PO_4_ as a base, and then in vitro antibacterial properties were examined. Carboxamide derivatives **69** were obtained by using Pd as a catalyst and K_3_PO_4_ as a base, allowing a Suzuki coupling reaction between boronic acids **4** and carboxamide **68**, which was formed by the reaction of furan **66** and aniline **67** in triethylamine base and dry dichloromethane (DCM). At different concentrations (10, 20, 30, 40, and 50 mg/well), molecules **68** and **69** were evaluated for antibacterial activity against XDR pathogens. However, compound **68** demonstrated exceptional activity, as evidenced by its MIC value of 6.25 mg and MBC value of 12.5 mg. Antibacterial properties have also been observed in compounds **69a** and **69b** (Scheme 12) [98].

**Scheme 13:**
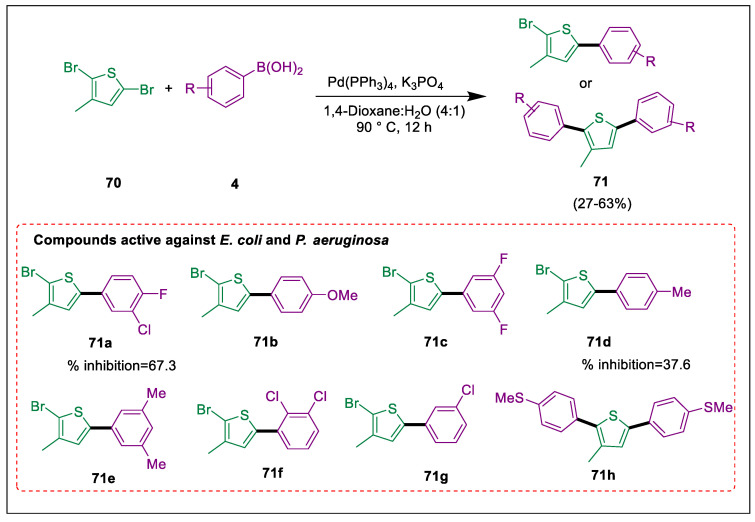
Synthesis of aryl thiophene derivatives via Pd-catalyzed Suzuki coupling reaction.

Several thiophene-based heterocycles, including biaryl thiophenes, exhibit a wide range of pharmacological actions [99,100,101,102,103,104]. It has been observed that 2,5-dibromo-3-hexylthiophene undergoes regioselective Suzuki cross-coupling; coupling ideally takes place at the C-5 position [105]. The lone pair on the heteroatom (O, S, and N) is donated to the ring in heterocycle substitution processes. By using the Suzuki coupling procedure, Rizwan et al. produced new compounds of 2,5-dibromo-3-methylthiophene **71** in moderate yields. Every examined substance exhibited encouraging biological activity. Utilizing the Suzuki reaction between 2,5-dibromo-3-methylthiophene **70** and arylboronic acid **4** in the presence of Pd catalyst, K_3_PO_4_ (2 eq), 1,4-dioxane, and 90 °C under argon, then aryl thiophenes **71** were synthesized. At 50 μg/mL, synthesized compounds **71a**, **71e**, **71f**, and **71g** displayed the highest percentage of inhibition against *P. aeruginosa* (67.3, 50.5, and 41.1%), while compounds **71b**, **71c**, **71d**, and **71h** showed moderate activity (39.2, 37.6, 34.9, and 20.8% inhibition). Compounds **71a**, **71e**, and **71g** demonstrated exceptional efficacy against *E. coli*, exhibiting 94.5, 72.5, and 70.4% inhibition, whereas compounds **71b**, **71d**, and **71h** showed a moderate inhibitory effect against the same strain of bacteria (Scheme 13) [106].

**Scheme 14:**
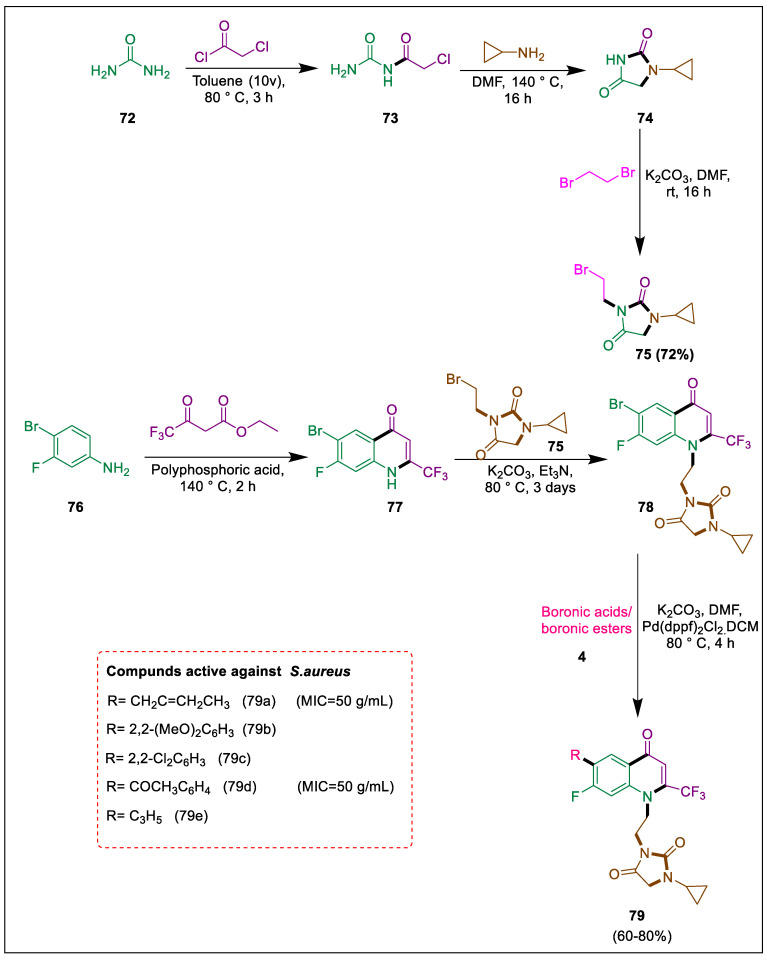
Synthesis of quinolone and hydantoin derivatives by Suzuki cross-coupling.

Drug-resistant infections are referred to as “superbugs” because they pose a serious threat to human society due to microbial resistance [107,108,109]. Nitrogen-containing heterocyclic chemicals are quinolone and its fluorine derivatives. Hydantoin, a heterocyclic chemical molecule, is recognized for its biological activities [110]. In medicine design, halogens, especially fluorine substituents, are the focus of extensive investigation. Mahajan et al. synthesized a range of novel trifluoromethyl-substituted quinolone and hydantoin hybrids and tested them against gram-positive and gram-negative bacteria. Compound **79a**, with the propene group on the quinolone ring, demonstrated comparable activity to conventional medication (chloramphenicol), with MIC values of 50 µg/mL against *P. aeruginosa* and *S. aureus*. After treating urea **72** with chloroacetyl chloride in the presence of toluene, chloroacetamide **73** was produced. This was then refluxed with cyclopropylamine in DMF for 16 h to produce dione **74**, and after that, compound **75** was produced by treating its solution with 1,2-dibromoethane in the presence of K_2_CO_3_. Subsequently, compound **77** was formed via the intermolecular condensation of aniline **76** and ethyl-4,4,4-trifluoro-3-oxobutanoate in polyphosphoric acid at 140 °C for two hours. In the presence of K_2_CO_3_, triethylamine, and DMF compound **77** reacted with **75** at 80 °C for 48 h to produce compound **78**, which in the presence of a Pd catalyst_,_ DCM, and potassium carbonate in DMF at 80 °C undergoes Suzuki coupling with boronic ester/boronic acid **4**, then quinolone and hydantoin hybrids **79** were obtained. Three compounds **79a**–**c** had equivalent inhibition zones to chloramphenicol, whereas **79d** and **79e** had comparable antibacterial activity against *S. aureus* (Scheme 14) [111].

**Scheme 15:**
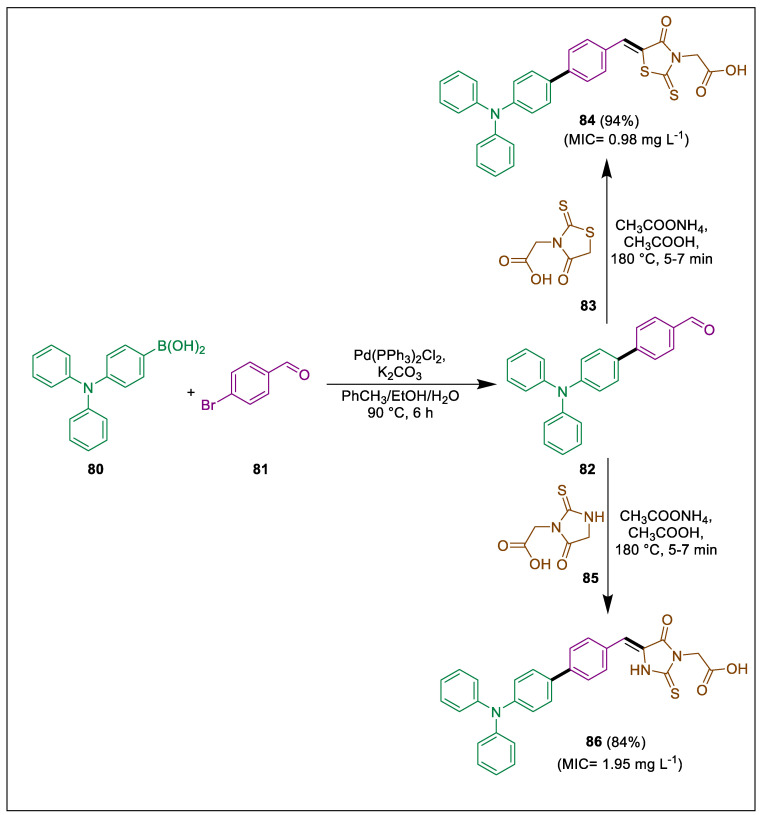
Synthesis of rhodanine and 2-thiohydantoin derivatives by using Suzuki cross-coupling.

Because of their biological action, 1,3-thiazolidine, imidazolidine, and their derivatives, including rhodanine or 2-2-thiohydantoin, have been studied [112]. Toma et al. also reported that an increase in antimicrobial activity resulted from adding a benzylidene substituent to rhodamine at position C-5 [113]. Tejchman et al. investigated the biological properties of twelve newly synthesized heterocyclic derivatives. The compounds under examination are based on 2-thiohydantoin and rhodanine cores that have carboxymethyl or hydrogen substituents. These cores are connected to a triphenylamine moiety through spacers at the C-5 position of the heterocycles, which are 1,4-phenylene, 1,9-anthracenylene, and 1,4-naphthalenylene. The compounds exhibit antibacterial properties. In the presence of PhCH_3_ and ethanol in H_2_O, an SMC Pd-catalyzed reaction occurs between 4-(diphenylamino)phenylboronic acid **80** and bromoarenecarbaldehydes **81** to form aldehydes **82**, which then go through Knoevenagel condensation with rhodanine **83** to form compound **84** and with 2-thiohydantoins **85** to form compound **86**. Rhodamine derivative **84** exhibited a greater capacity to impede bacterial growth than 2-thiohydantoin derivative **86**, which demonstrated greater bactericidal activity against *M. luteus* (Scheme 15) [114].

**Scheme 16:**
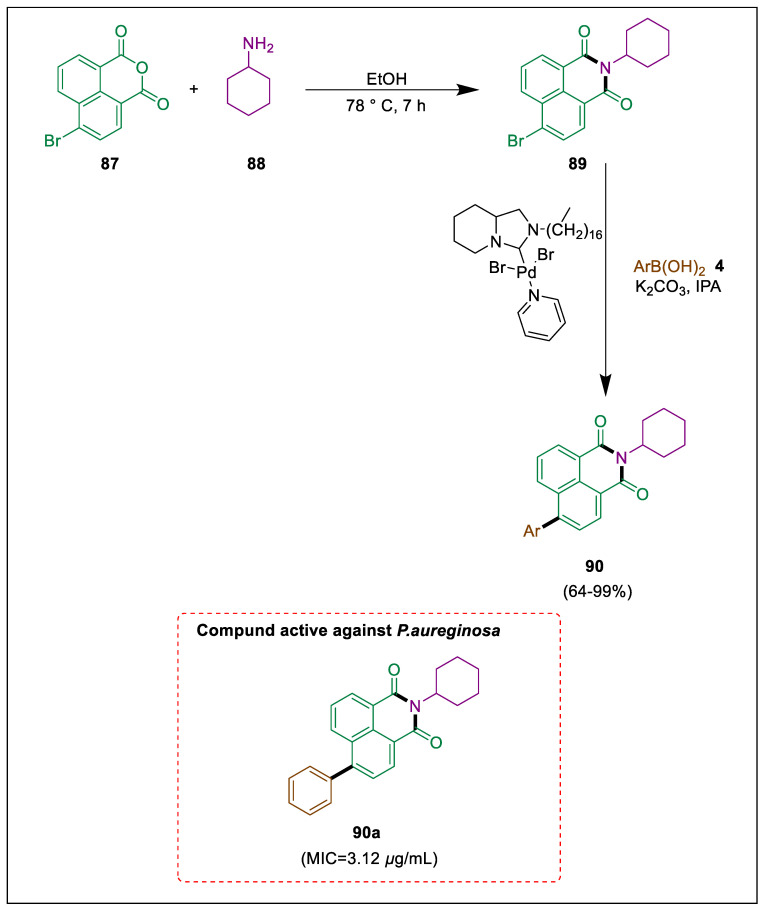
Synthesis of naphthalimide derivatives by using Suzuki-Miyaura cross-coupling.

1,8-Naphthalimide dyes are biologically active and employed as chemosensors, liquid crystal additives, and cell imaging agents [115,116,117]. A Suzuki-Miyaura (SM) cross-coupling process is one method using the naphthalene ring to form novel structures. Using an SM coupling process, a fluorescent chemosensor molecule with rhodamine and naphthalimide attached was synthesized [118]. Türkmen et al. used the catalyst NHC-Pd (II) complex and potassium carbonate in isopropyl alcohol (IPA) under modest conditions to manufacture 4-phenyl-1,8-naphthalimide derivatives from compound **89** via Suzuki coupling reactions. By calculating MIC values, the antimicrobial properties of the synthetic dyes were assessed against a set of six microorganisms. Naphthalic anhydride **87** and cyclohexylamine **88** in EtOH refluxed for seven hours, then 4-bromo-*N*-(cyclohexyl)-1,8-naphthalimide **89** was formed, which synthesized new 1,8-naphthalimide dyes **90** using a Suzuki coupling process with boronic acids **4** in the presence of a Pd catalyst. Compared to alternative dyes, **90a** shows high antibacterial activity against *P. aureginosa* with a MIC value of 0.095 μg/mL (Scheme 16) [119].

**Scheme 17:**
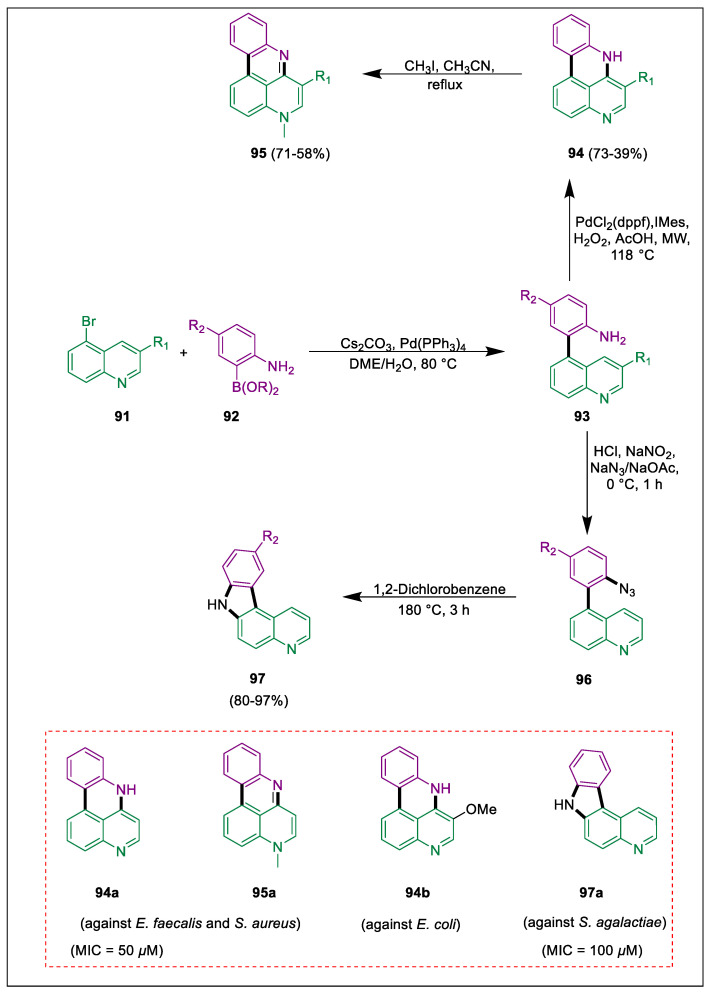
Synthesis of pyridophenanthridines and pyridocarbazole derivatives by using Suzuki cross-coupling.

Cancer and malaria are two serious health problems that claim millions of lives every year. Apart from its critical role in the synthesis of therapeutic chemicals, the quinoline core is a structural motif that finds extensive application in the fields of material science and the dye industry [120]. Indoloquinoline natural components represent an ideal class of bioactive compounds. Håheim et al. synthesized a sequence of novel quinoline-based tetracyclic ring systems and assessed them in vitro for their biological activities. The pyridophenanthridine **94** is also active against some strains of gram-positive and gram-negative bacteria, alongside the compound **94b** being active against *E. coli* (MIC = 50 µM) and *S. agalactiae* (MIC = 75 µM). Compounds quinoline **91** and boronic esters **92** cross-couple via the Suzuki-Miyaura process to generate biaryl compounds **93**. These compounds then move via pathway A to form pyridophenanthridine scaffolds **94**, which *N*-methylate using excess iodomethane in refluxing acetonitrile to form pyridophenanthridines **95**. Derivative pyridocarbazole **97** is formed when pathway B is followed. **97a** exhibited bacteriostatic characteristics against Gram-(+) bacteria and showed modest efficacy against *S. agalactiae* (MIC = 100 µM). excluding *P. aeruginosa*. While **94b** was only marginally effective against *E. coli*, **94a** and **95a** were both effective against *S. aureus* and *E. faecalis* (Scheme 17) [121].

**Scheme 18:**
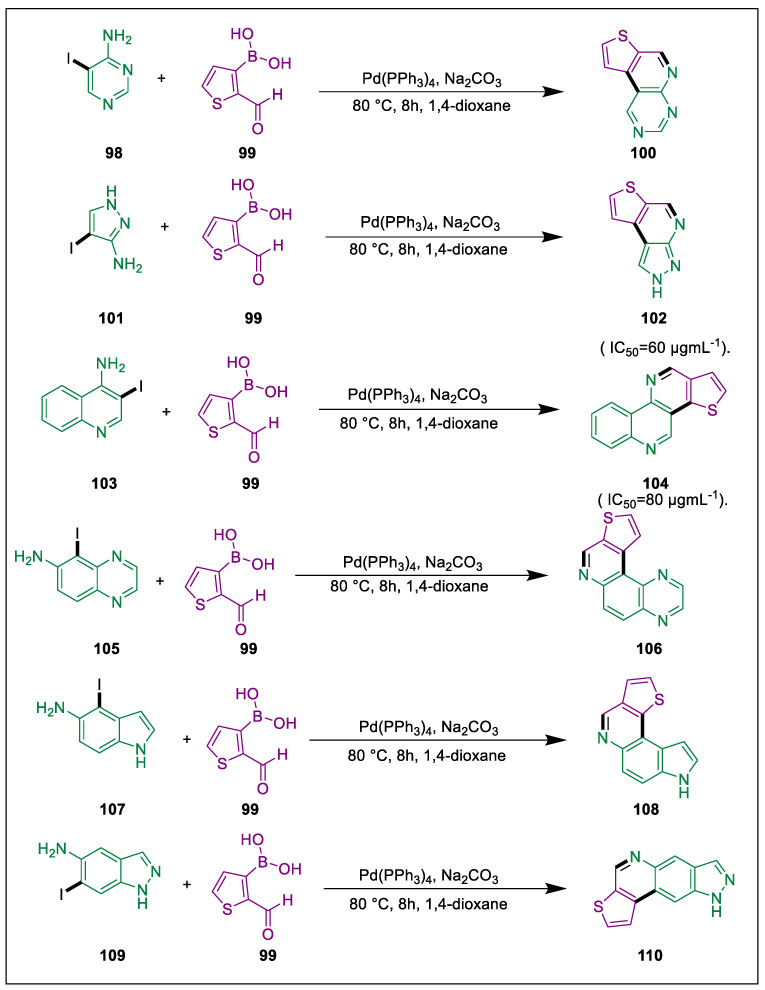
Synthesis of thieno nucleus containing hetero-aryls by using Suzuki cross-coupling.

Several heterocyclic substructures are employed in the formation of physiologically active substances [122]. A pharmacophore used in the synthesis of strong, physiologically active substances is the thieno nucleus [123,124]. A library of thieno nucleus-fused heterocyclic compounds containing two or three bioactive pharmacophores in a single molecule was developed using Suzuki coupling. Using bis(triphenylphosphine)palladium(II) dichloride, Sain et al. produced thieno nucleus-adorned trinuclear and tetranuclear nitrogen heteroaryl via the Suzuki cross-coupling reaction. In vitro antibacterial studies of synthesized compounds against gram-positive and negative strains were determined. In the presence of [Pd(PPh_3_)_2_Cl_2_], a mixture of 2-formyl-3-thienylboronic acid **99** with heteroaryl iodides (**98**, **101**, **103**, **105**, **107**, **109**) undergoes Suzuki coupling in 1,4-dioxane stirred for 30 min. After that, the mixture is degassed, Na_2_CO_3_ solution is added, the mixture refluxes under a nitrogen atmosphere, and the coupling products (**100**, **102**, **104**, **106**, **108**, **110**) are obtained by evaporation of methanol. Test compounds **102** and **104** demonstrated significant inhibitory efficacy against all targeted bacterial strains except pseudomonas (Scheme 18) [125].

**Scheme 19:**
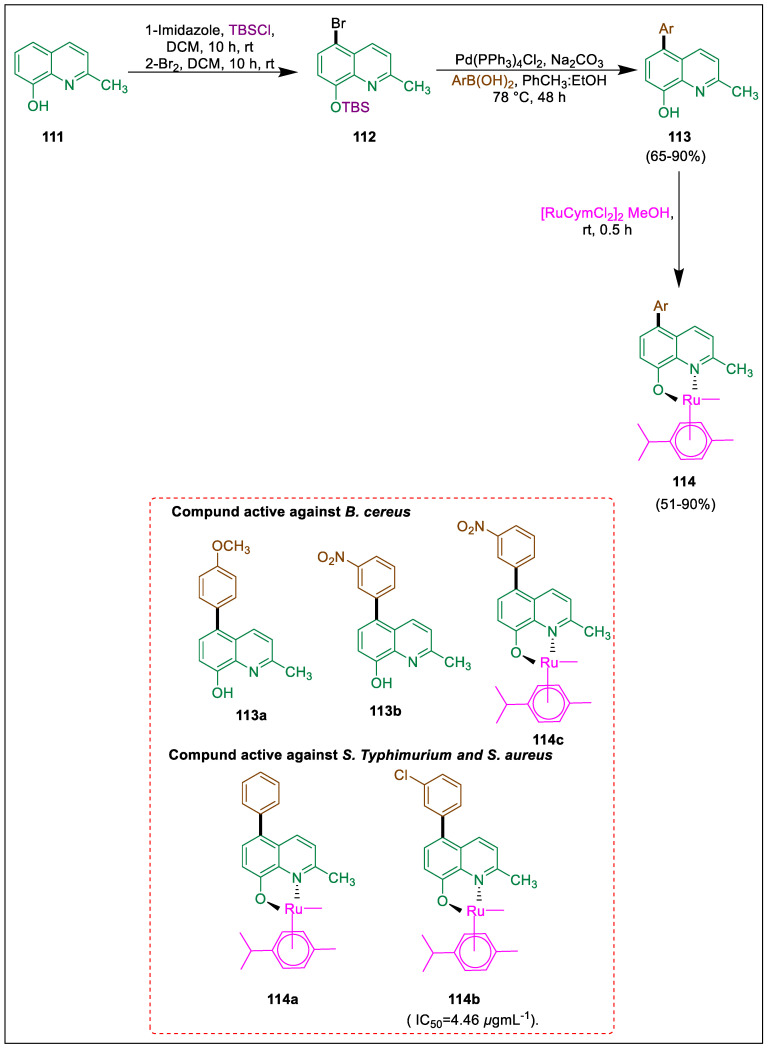
Synthesis of arylmethylquinolin ligands and their ruthenium complexes by using Suzuki cross-coupling.

Because of their biological characteristics, a novel class of ruthenium complexes has been discovered to represent intriguing pharmacological possibilities [126]. Pyrazolone ligands exhibit impressive performance with Ru-II, and when combined with 5,7-dibromo-8-hydroxyquinoline, then this complex shows increased antibacterial activity. The synthesis of 5,7-dihalogenated-2-methyl-8-quinolinol and its equipotent anticancer efficacy were documented by Meng et al. [127]. Perez et al. used a Suzuki cross-coupling reaction to synthesize eight arylmethylquinolin ligands **113** from precursors brominated at positions 5 and 7. These ligands were converted into ruthenium(II)-*p*-cymene **114** novel complexes. All compounds were examined for their cytotoxic and antibacterial properties. Compared to ruthenium complexes (EC_50_ = 5.2–7.8 μM), ligands exhibited greater cytotoxicity (EC_50_ = 3.1–4.8 μM). *Tert*-butyldimethylsilyl chloride (TBSCl) was used to treat 2-methyl-quinoline-8-ol **111** in the presence of imidazole. The next step was adding bromine to form arylbromide **112**, which then produced eight arylquinoline intermediates when it interacted with different aryl boronic acids in the presence of a Pd(PPh_3_)_4_ catalyst. After treating these intermediates with acid to eliminate the TBS group, the required product **113** was obtained. Handling of [RuCymCl_2_]Cl_2_ (ruthenium dimer) along with each derivative **113** of 2-methylquinolin-8-ol [6] produced the required complexes **114**. Compound **122a** was capable of inhibiting *S. aureus*. Complexes **114** exhibit IC_50_ values ranging from 4.64 to 146.15 μgmL^−1^, indicating more activity against *S. Typhimurium*. The most effective compounds against *S. Typhimurium* and *S. aureus* are **114a** and **114b**, while compounds **113a**, **113b**, and **114c** are active against *B. cereus* (Scheme 19) [128].

**Scheme 20:**
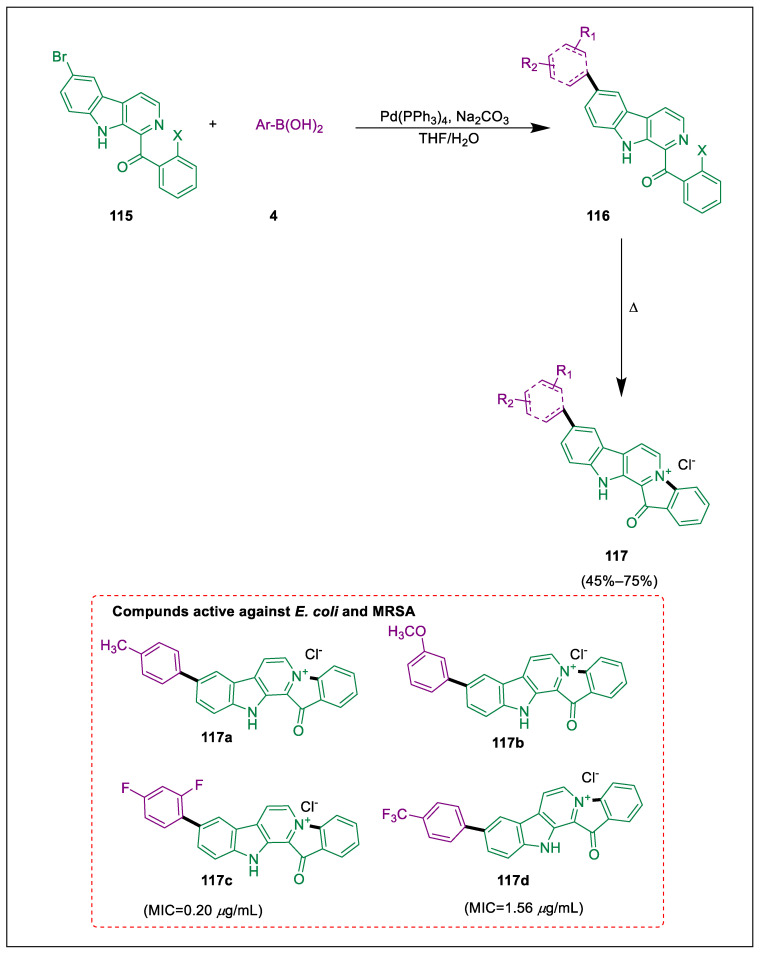
Synthesis of fascaplysin structural hybrids by using Suzuki-Miyaura cross coupling.

Marine natural materials are employed to produce lead compounds in medication development because of their uniqueness and diversity [129]. A marine *β*-carboline alkaloid called facsaplysin was taken out of a sponge Fijian. Numerous biological actions of fascaplysin have been documented, including the potency of amide-modified derivatives of fascaplysin against MRSA [130]. Suzuki-Miyaura coupling forms carbon-carbon bonds by using palladium as a catalyst [131]. Jiang et al. developed a novel synthesis process for fascaplysin derivatives, which is produced using Suzuki-Miyaura coupling that is regioselective and then quaternized. Particularly against the gram-negative bacterium *E. coli*, certain synthetic fascaplysin derivatives showed stronger antibacterial activity in comparison to pristine fascaplysin. By using regioselective Suzuki-Miyaura cross-coupling, bromo-chloro *β*-carboline **115** couples with other boronic acids **4** in the presence of Na_2_CO_3_, Pd-catalyst, and THF/H_2_O to synthesize fascaplysin precursors **116**. The fascaplysin A-ring derivatives **117** were synthesized by employing a few compounds that have typical characteristic functional groups. Compound **117c**, in combination with 2,4-difluorophenyl, demonstrated the greatest antibacterial activity against MRSA with MIC values of 0.20 μg/mL, whereas compounds **117a**–**d** showed increased anti-MRSA activity. With a MIC value of 1.56 μg/mL, compounds **117c** and **117d** had the strongest antibacterial activity against *E. coli* (Scheme 20) [132].

**Scheme 21:**
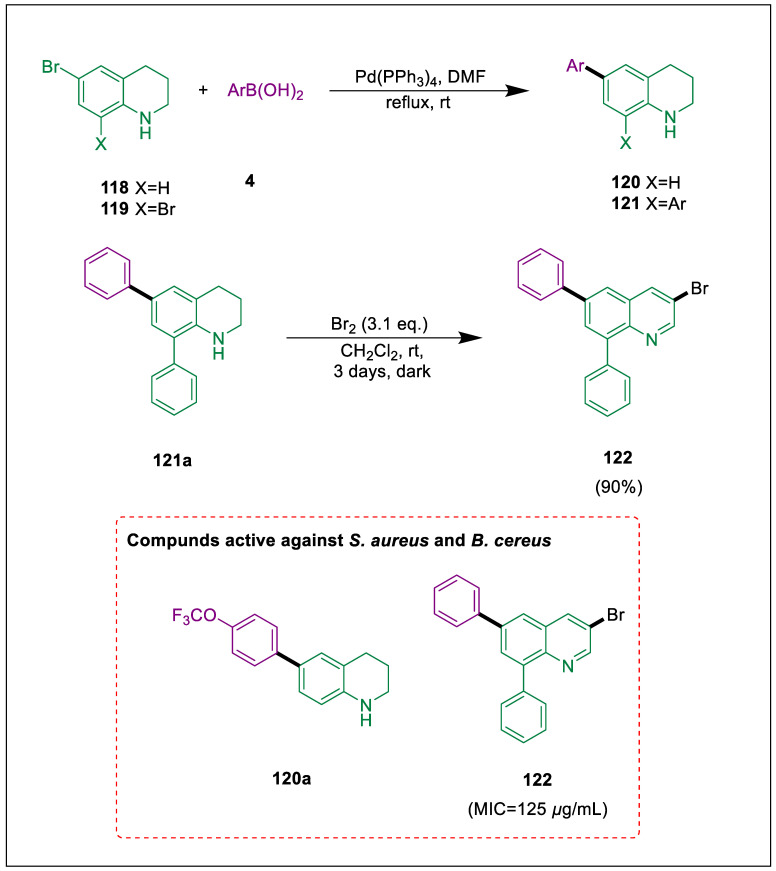
Synthesis of tetrahydroquinolines derivatives by using Suzuki-Miyaura cross-coupling.

Since quinoline derivatives have well-established therapeutic benefits, they have been designated as synthetic intermediates in drug research [133,134], and the quinoline moiety can be found in several scaffolds that are both pharmaceutically and physiologically significant. The derivatives of halogenated tetrahydroquinoline are significant intermediates [135,136,137,138]. The Suzuki-Miyaura cross-coupling process works well for making arylated quinolones. By treating bromo tetrahydroquinoline with boronic acids in the presence of a Pd catalyst, Koçyiğit et al. produced phenyl tetrahydroquinolines **120a** and **121a** in high yields. Next, a new 8-bromo-6-pheyltetrahydroquinoline was produced by bromination of diphenyl tetrahydroquinolines **121a**. Bromo tetrahydroquinolines **118** and **119** undergo Suzuki-Miyaura cross-coupling with boronic acids **4** synthesized 6-aryl substituted **120** and 6,8-diaryl substituted tetrahydroquinolines **121**. By brominating 6,8-diphenyl-1,2,3,4-tetrahydroquinoline **121a**, compound **122** (3-bromo-6,8-diphenylquinoline) was synthesized. With MIC values ranging from 125–250 μg/mL, two compounds **120a** and **122** have moderately reduced the proliferation of all gram-positive bacteria (Scheme 21) [139].

**Scheme 22:**
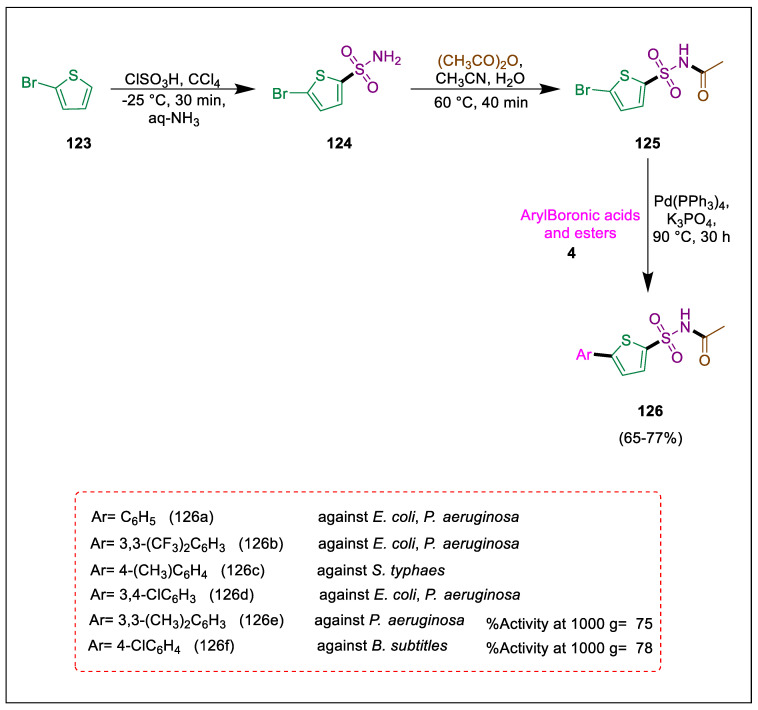
Synthesis of sulphonyl acetamide derivatives by using Suzuki-Miyaura cross-coupling.

*N*-acyl sulfonamide (sulfacetamide) is a common basic structural motif [140]. This is found as a functional group in many different therapies [141]. According to several pharmaceutical patents, aryl sulfonamides have a broad spectrum of biological properties and could be used as medicinal agents [142] and have huge significance in drug chemistry because of their several biological activities. Noreen et al. using Pd-catalyzed Suzuki cross-coupling processes, produced a range of new 5-arylthiophenes **126** with sulphonylacetamide (sulfacetamide) groups in significant yields. The agar-well diffusion method is used to assess the antibacterial activities of these compounds, whereas the indophenol method is used to determine their anti-urease activities. After 2-bromo-thiophene **123** reacts with PCl_5_ and chlorosulfonic acid, it forms 5-bromothiophene-2-sulfonamide **124**. This compound then reacts with ethanoic anhydride in acetonitrile with a few drops of H_2_SO_4_ to form acetamide **125**, which then undergoes the Suzuki reaction with various arylboronic acids **4** and boronic esters to produce compound **126**. Compounds **126c** displayed a high activity against *S. typhaes*, **126f** showed the highest activity against *Bacillus subtitles*, and **126e** demonstrated activity against *Pseudomonas aeruginosa*. Compounds **126a**,**b** and **126d** show the highest activity against *E. coli* and also demonstrated the highest activity against *P. aeruginosa* (Scheme 22) [143].

**Scheme 23:**
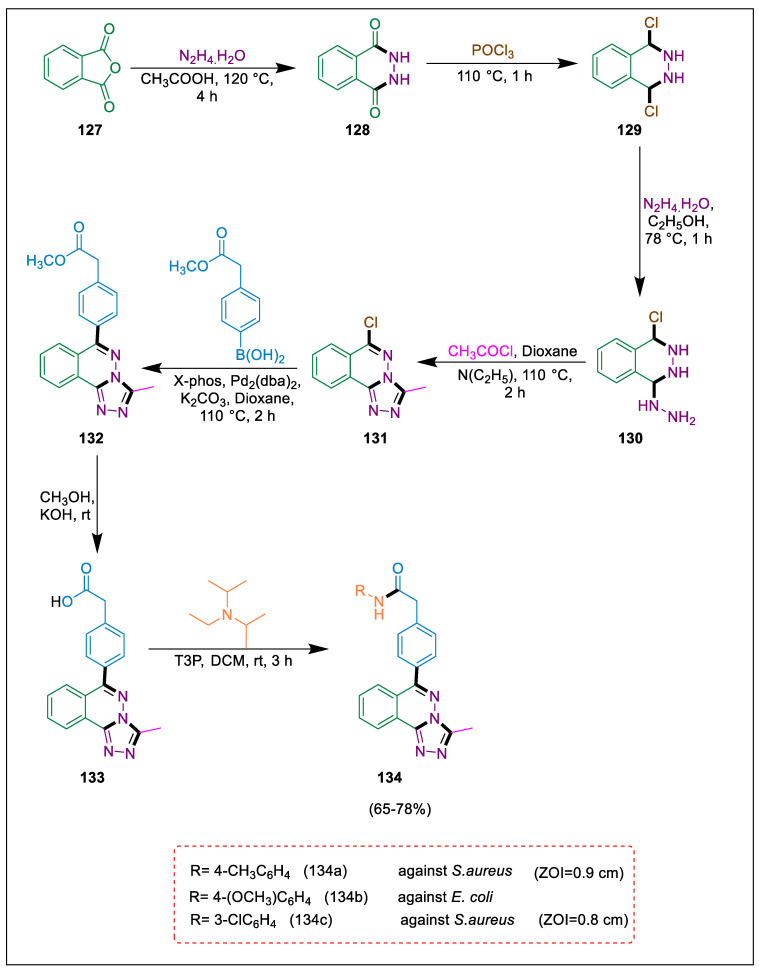
Synthesis of triazolo phthalazine derivatives by using Suzuki cross-coupling.

Heterocycles exhibit biological activity and are important pharmacophoric components in both synthetic and natural medicinal compounds. Heterocyclic compounds that include nitrogen, such as phthalazine derivatives, provide the structural framework for biologically active molecules [144,145,146,147]. Derivatives of 1,2,4-trizole also exhibit a wide range of biological functions. HCT116 is one of the most extensively researched in vitro colorectal cancer cell lines. Using Suzuki coupling, Kumar et al. synthesized several novel *N*-aryl substituted phenyl acetamide analogues phthalazines. The process began with readily accessible, reasonably priced phthalic anhydride. These substances were tested for their capacity to inhibit the HCT116 cancer cell line using the MIT assay. Additionally, compounds were examined for antibacterial properties. Under reflux conditions, commercially available phthalic anhydride **127** reacted with hydrazine hydrate in methane carboxylic acid to yield phthalazine **128**, which was then treated with phosphorus oxychloride to produce 1,4-dichlorophthalazine **129**. This compound was then treated with hydrazine hydrate to produce 1-choro-4-hydrazinophthalazine **130**, which dissolved in dioxane, triethylamine, and acetyl chloride to obtain the compound **131**, and it was then treated with Boronic acid in the presence of (Pd_2_(dba)_2_) to obtain ester derivatives **132** through the Suzuki reaction, which undergo hydrolysis by using KOH in methanol to form acid derivative **133**, which reacts with various aromatic amines and propyl phosphonic anhydride (T_3_P) reagent to yield phthalazine derivatives **134**. Compared to tetracycline, compounds **134a** and **134c** were shown to be moderately active against *S. aureus*, whereas compounds **133** and **134b** were also moderately active against *E. coli* (Scheme 23) [148].

**Scheme 24:**
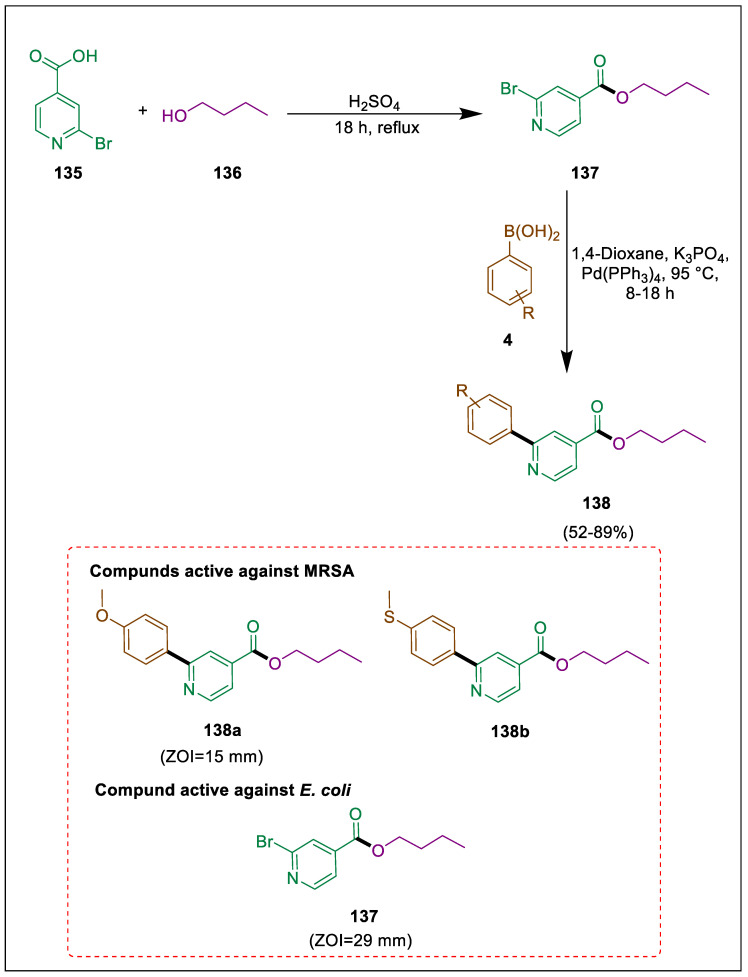
Synthesis of arylated butyl bromoisonicotinate derivatives by using Suzuki-Miyaura cross-coupling.

Reducing the emergence of microbial resistance and developing new antibacterial medications are vital because antimicrobial drugs make it more difficult to treat evolving MDR infections using currently available drugs [149]. They can be treated only with antibiotics such as colistin, which is very harmful to the human body [150]. Nicotinic acid derivatives show anti-tubular [151], anti-lipolytic [152], and anti-viral activities [153]. Fischer esterification was utilized by Naheed et al. to synthesize butyl-2-bromoisonicotinate, which was subsequently arylated to produce arylated butyl 2-bromoisonicotinates using a palladium catalyst. In vitro antibacterial activities of molecules (**137**, **138**) were screened against clinically isolated ESBL *E. coli* ST405 and MRSA. Using a catalytic amount of sulphuric acid (H_2_SO_4_), commercially available isonicotinic acid **135** reacts with butanol **136** to form butyl 2-bromoisonicotinate **137**. This compound then reacts with various boronic acids **4** in the presence of K_3_PO_4_ and palladium catalyst to form arylated butyl 2-bromoisonicotinate **138** via SMC. The results showed that, with a 29 mm zone of inhibition against the ESBL-producing *E. coli* ST405, molecule **137** was the most effective, while molecules **138a** and **138b** were the most effective against the MRSA strain, exhibiting 24 mm and 21 mm zones of inhibition, respectively (Scheme 24) [154].

**Scheme 25:**
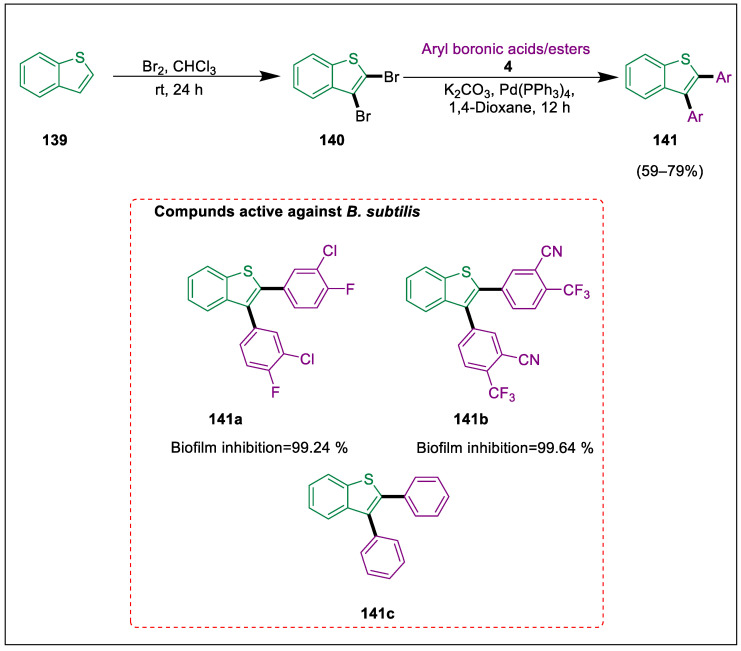
Synthesis of benzo-thiophene derivatives by using Suzuki-Miyaura cross-coupling.

Heterocyclic compounds are synthesized in labs to be used as therapeutic agents and play a vital role in medicinal chemistry [155]. Heterocyclic ring systems, alongside particular substitutions, behave like strong scaffolds for several biological activities [156]. Benzo[*b*]thiophene is a special type of heterocyclic based on sulfur that is found in bioactive molecules; its diaryl derivatives are not as well documented as they could be. Using Suzuki coupling processes, Sial et al. produced a range of 2,3-diaryldibenzo[*b*]thiophene derivatives in moderate to good yields. The hemolytic potential, biofilm inhibition, and antithrombolytic properties of the produced compounds were assessed. Every compound had considerable biological potential. Benzo[*b*]thiophene **139** was brominated, and 2,3-dibromobenzo[*b*]thiophene **140** was the intermediate chemical that was produced. This compound reacted with different boronic acids/esters **4** in the presence of a palladium catalyst, resulting in the synthesis of the benzothiophene derivatives **141** via the Suzuki reaction. All compounds are active to varied degrees, ranging from moderate **141c** to very high **141a**, **141b**. Compound **141b**, which is most active with 99.64% biofilm suppression of *B. subtilis*, with cyano and trifluoromethyl groups (Scheme 25) [156].

**Scheme 26:**
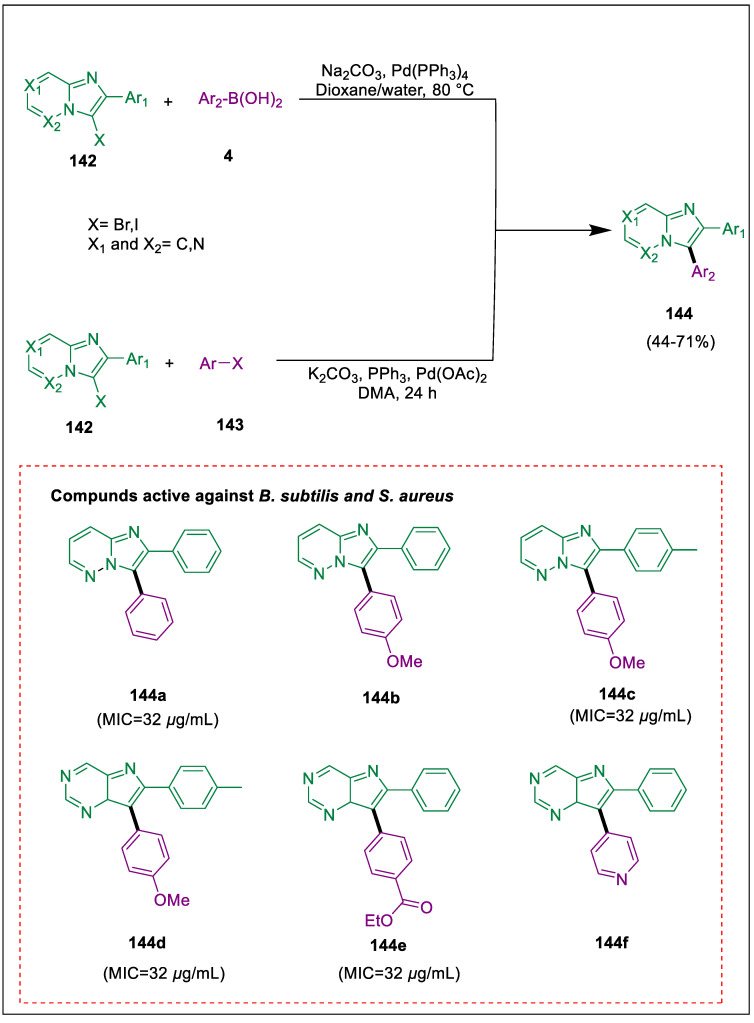
Synthesis of diarylimidazo pyrazine derivatives by using Suzuki-Miyaura cross-coupling.

Because of their possible biological activity, imidazo pyrazines, a family of nitrogen-containing bridgehead moieties, have drawn a lot of attention [157]. 2,3-Diarylimidazo pyridazine is a well-known molecule with a variety of biological characteristics. The most popular techniques for arylating these scaffolds are palladium-catalyzed direct arylations and Suzuki cross-couplings [158,159]. Two effective synthetic methods for the synthesis of diarylimidazo pyrazines have been synthesized by Soltani et al. The steps include a direct arylation at position 3 catalyzed by a palladium Suzuki cross-coupling reaction. The produced compounds have moderate to good antibacterial properties, according to measurements. Direct arylation and Suzuki cross-coupling of imidazo-pyrazines/pyridazines **142** with a range of boronic acids **4** and aryl halides **143** to obtain the required products diarylimidazo pyrazines **144**. Compounds **144a**–**f** showed high effectiveness against *B. subtilis* and *S. aureus* (Scheme 26) [160].

**Scheme 27:**
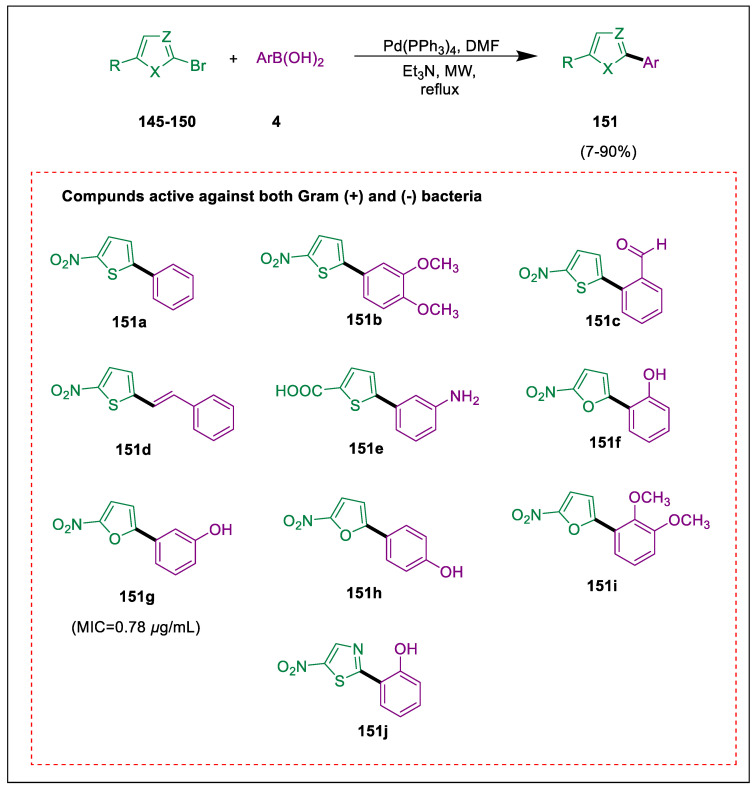
Synthesis of thiophene, furan, and thiazole derivatives by using Suzuki cross-coupling.

Antibiotics have revolutionized medicine, and once-fatal infections may now be treated easily. Antibacterial resistance is induced by antibiotics, even at trace levels [161,162,163]. Thiophenes, which are heterocyclic chemicals, are significant because of their pharmacological characteristics and ease of synthesis [164,165,166]. Additionally, furan-containing compounds are used in many different therapeutic applications [167]. Hurtado et al. used SMC and microwave irradiation to synthesize thiophene, furan, and thiazole derivatives. Numerous substances exhibit widespread antibacterial action. Significant antibacterial activity was demonstrated by **151g** against *Salmonella enterica* having a MIC value of 0.78 µg/mL and *Streptococcus pyogenes* having a MIC value of 0.097 µg/mL. Compounds **151** are synthesized using the SMC process and microwave irradiation in the presence of palladium catalyst, base, and solvent from bromo-heterocycles **145**–**150** and boronic acids **4**. Nine compounds, such as **151a**–**j**, displayed growth inhibition, but only compounds **151d** and **151f**–**i** showed inhibition rings like ampicillin (10 µg/dis) control. **151g** is most active against both gram-positive and gram-negative bacteria (Scheme 27) [168].

**Scheme 28:**
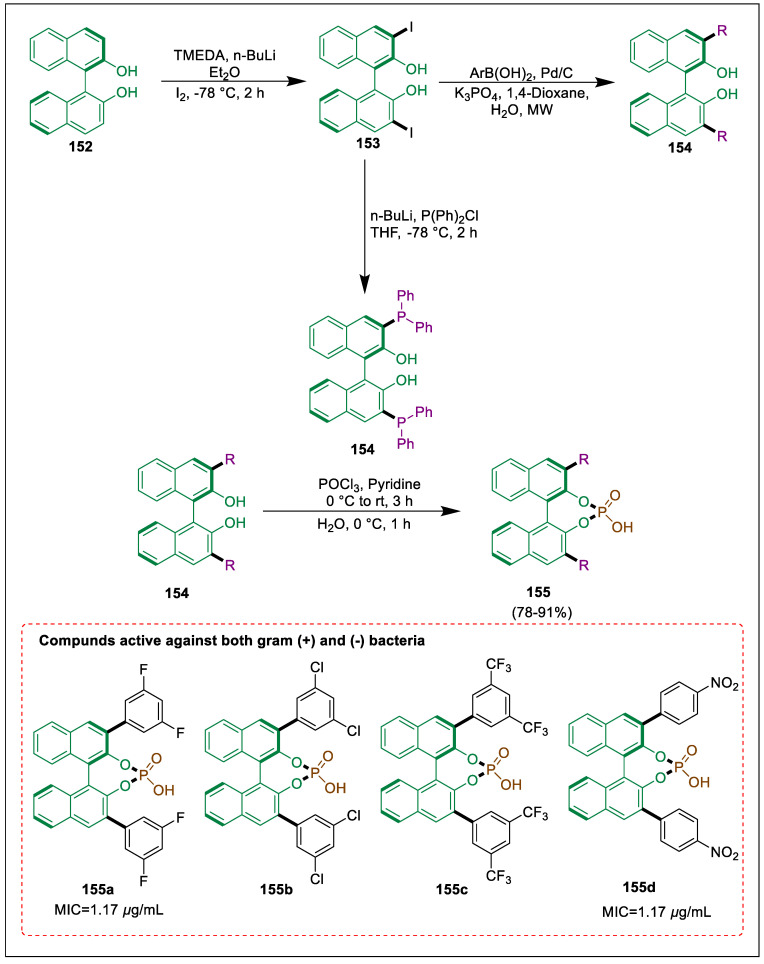
Synthesis of BINOL-derived phosphoric acid derivatives by using SMC.

Axial chiral phosphoric acids, such as those formed from BINOL, have demonstrated their usefulness as catalysts in a variety of processes. Similarly, 3,3′-disubstituted phosphoric acids derived from BINOL are employed in highly enantioselective reactions. As a result, organocatalysis is of great interest for the synthesis of new Bronsted catalysts having chirality and for improving their manufacturing process [169,170]. Using the BINOL derivative, which is not protected Konda et al. synthesized a new class of 3,3′-disubstituted chiral (*R*)-BINOL-derived phosphoric acid derivatives and optimized them by catalyzing with Pd/C in the SMC. The antibacterial and *α*-glucosidase inhibitory properties of the target compounds have been evaluated. *ortho*-lithiation of (*R*)-binaphthalene diol **152** in the presence of tetramethylethylenediamine followed by reaction with iodine produced (*R*)-diiodo binaphthalene diol **153**, which underwent Suzuki coupling with aryl boronic acids to yield 3,3′-disubstituted chiral (*R*)-BINOL derivatives **154**. One of the derivatives **154** was produced via the double *ortho*-lithiation of two in the presence of tetramethylethylenediamine and the subsequent reaction with diphenylphosphinous chloride. **154** were phosphorylated with POCl_3_ in pyridine, which formed 3,3′-disubstituted (*R*)-BINOL-derived phosphoric acids **155** having chirality. Compound **155d** showed the highest level of activity, with MIC values ranging from 1.17 to 2.34 μg/mL. Among the compounds, **155a**–**d** showed strong antibacterial activity against both gram-positive and gram-negative pathogens (Scheme 28) [171].

**Scheme 29:**
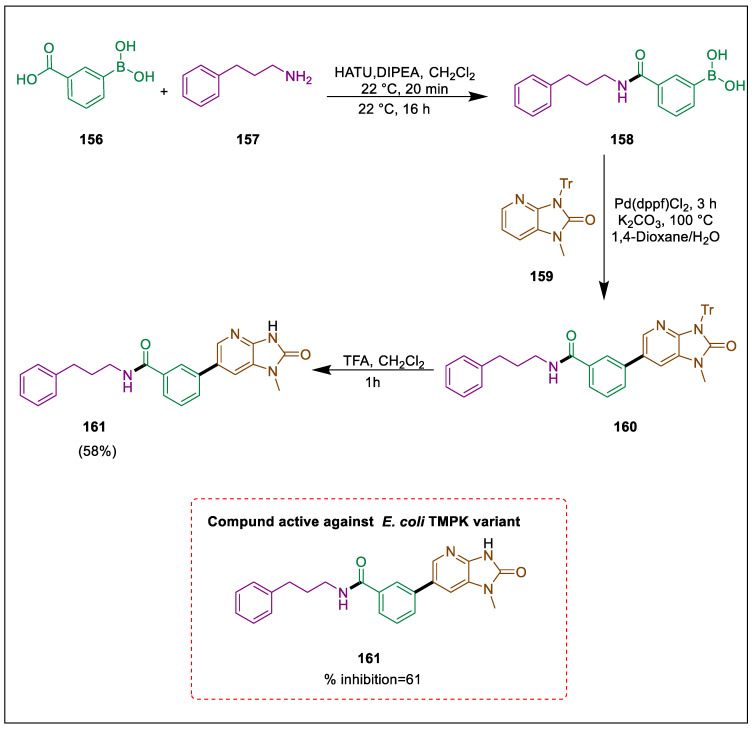
Synthesis of benzamide derivative by using Suzuki cross-coupling.

The development of novel antimicrobial agents has become imperative due to the proliferation of antibiotic resistance [172]. An essential enzyme, thymidylate monophosphate kinase (TMPK), could be a potential target for novel antibiotics [173]. Selective inhibition is possible because the bacterial and human TMPKs differ sufficiently in their sequences. In recent years, the emergence of other classes of inhibitors, such as the imidazopyridinone developed by Choi et al. [174], evaluated for biological activity. Imidazopyridinones are an important class of heterocyclic compounds synthesized by Blindheim et al. The inhibitory effects of the imidazopyridine on the *E. coli* thymidylate monophosphate kinase were significant. Using an amide coupling between bromobenzoic acid **156** and 3-phenyl-1-propylamine **157** were assembled by using HATU and DIPEA to form boronic acid **158** following a Suzuki coupling with Pd(dppf)Cl_2_, and analog **159** produced the protected analog **160**, and the target compound benzamide derivative imidazopyridine **161** was obtained in a 58% yield after deprotection with TFA in CH_2_Cl_2_. [175] Compound **161** has been identified as an inhibitor of TMPK in *E. coli*. At 8.3 μM, single-point inhibition measurements determined that imidazopyridinones **161** exhibited a good effect (Scheme 29) [175].

**Scheme 30:**
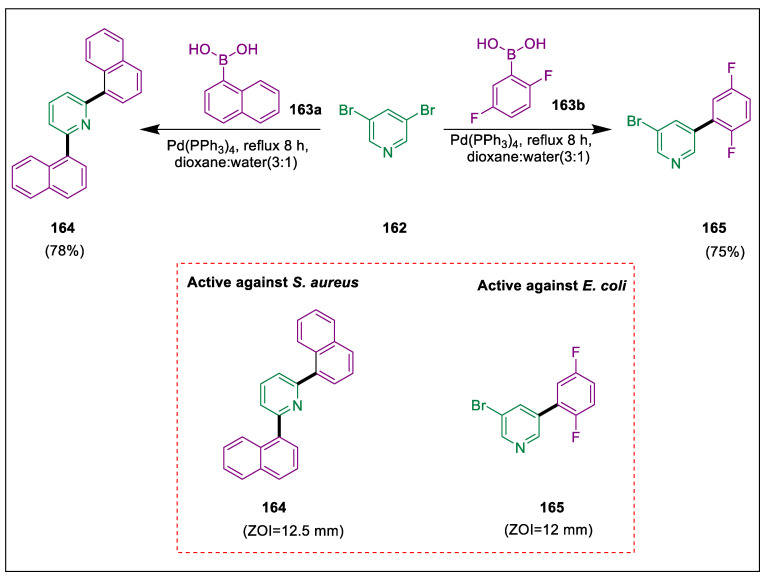
Synthesis of pyridine derivatives by using Suzuki cross-coupling.

Pd catalyst was employed in Heck, Stille, Suzuki, Sonogashira, and Buchwald-Hartwig couplings to generate carbon-carbon and carbon-heteroatom bonds. These reactions are significant in the production of medicinal agrochemicals. The research on pyridine and its derivatives shows that several of these substances are used for the treatment of many illnesses [176,177,178]. Through carbon-carbon coupling, Ghiasuddin et al. synthesized two novel pyridine derivatives: **164** and **165**. The experimental activity of compounds **164** and **165** in terms of zones of inhibition against fungus and bacteria has validated their bioactivity. In the presence of K_3_PO_4_, Pd catalyst, and dioxane, a one-pot reaction combining 3,5-dibromopyridine **162** and 1-naphthyleneboronic acid **163a** yields pyridine derivative **164**, whereas the use of 2,5-difluorophenylboronic acid **163b** yields pyridine derivative **165**. Compound **165** demonstrated maximum zone of inhibition of 12 mm against *S. aureus*. Likewise, compound **164** showed the highest value of inhibition of 12.5 mm against *E. coli* (Scheme 30) [179].

**Scheme 31:**
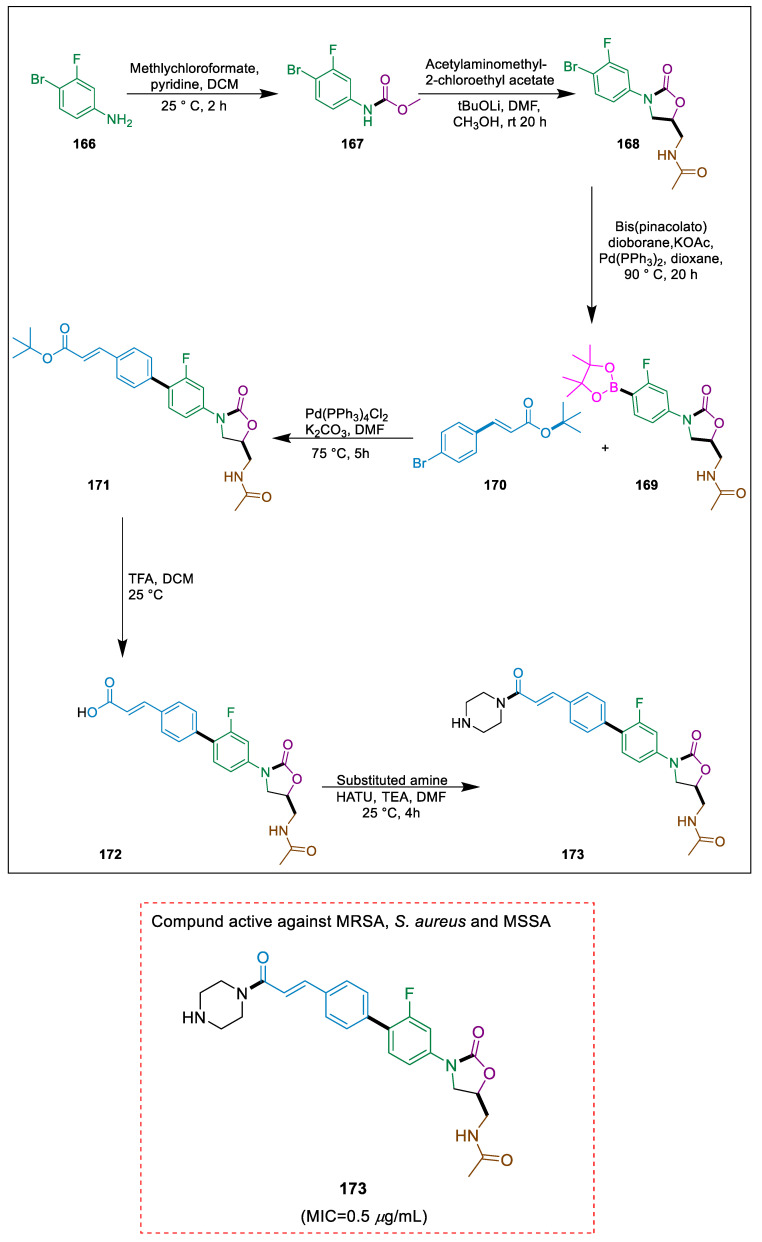
Synthesis of acetamide derivatives by using Suzuki cross-coupling.

Morbidity and death are significantly increased by infectious diseases [180]. Comparing oxazolidinones to other antibacterial medications, they have a distinct mechanism and low cross-resistance. Analogs of biarylloxazolidinone having a hydrazone moiety exhibited remarkable antibacterial activity [181]. To assess their antibacterial efficacy, Xu et al. synthesized several new biaryloxazolidinone derivatives with amide and acrylamide structures. Whereas most compounds showed strong antibacterial activity overall. Using 4-bromobenzaldehyde **166** as the starting material, a two-step reaction yields intermediate **169**, which is then Suzuki coupled with compound **170** to produce intermediate **171**. This intermediate is then treated with TFA to produce **172** and its condensation with different amines to make the target product acetamide derivative **173**. Compound **173** demonstrated good antibacterial activity, with MIC values of 0.5 μg/mL against MRSA, *S. aureus,* and MSSA (Scheme 31) [182].

### 2.2. Sonogashira Cross-Coupling

**Scheme 32:**
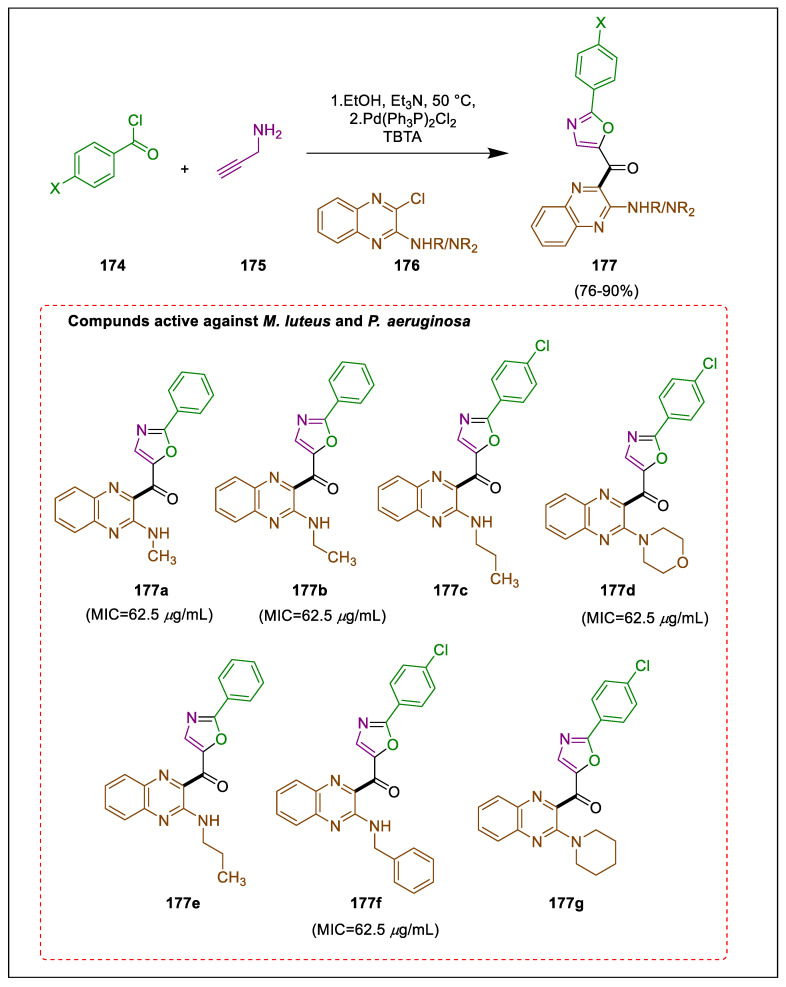
Synthesis of quinoxaline amine derivatives via Sonogashira cross-coupling.

A palladium complex conventionally facilitates Sonogashira coupling, while copper iodide serves as the co-catalyst when an amine is present [183,184]. Numerous coupling reactions devoid of copper have been documented [185]. Oxazoles of 2-aryl are the most important heterocycles because of their wide range of physical and biological properties [186]. Quinoxaline derivatives hold significant importance as heterocyclic molecules due to their utility as intermediates in organic chemistry and their manifestation of natural properties. In their study, Keivanloo et al. employed a copper-free Sonogashira coupling reaction to produce oxazole-quinoxaline amine hybrids by using 2-amine substituted chloroquinoxalines, prop-2-yn-1-amine, and benzoyl chloride compounds that catalyzed this reaction by using a Pd catalyst. The ligand utilized was TBTA, an effective ligand in ethanol. All prepared compounds underwent testing against *Micrococcus luteus* and *Pseudomonas aeruginosa*, two bacterial strains. Sonogashira coupling without copper is utilized in the synthesis of oxazole-quinoxaline amine hybrids. The desired product was produced by combining benzene carbonyl chloride **174** and propargylamine **175** in the presence of trimethylamine in ethanol at ambient temperature for two hours, followed by the addition of 2-amine substituted chloroquinoxalines **176** and Pd catalyst. The mixture was stirred for an additional eight hours to obtain oxazole-quinoxaline amine hybrids **177**. The antibacterial activities of compounds **177a**–**g** are superior against *Micrococcus luteus* and *Pseudomonas aeruginosa* to those of the other compounds. Furthermore, the antibacterial properties of **177a**, **177b**, **177d**, and **177f** exhibited a suppression level equivalent to amoxicillin (Scheme 32) [187].

**Scheme 33:**
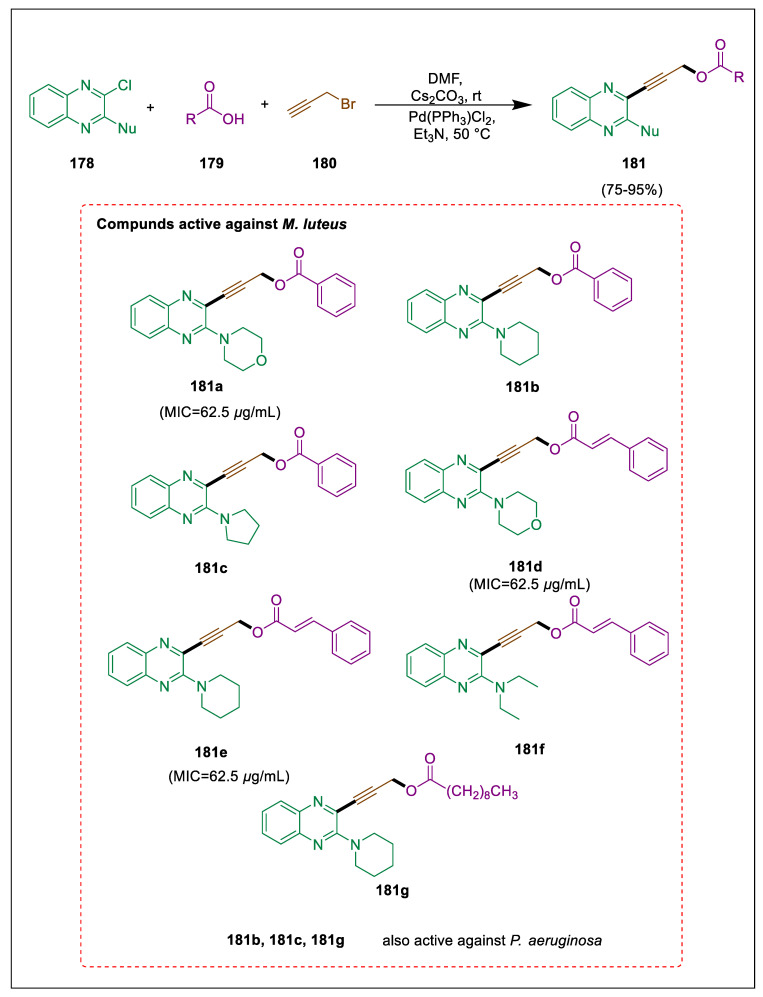
Synthesis of carboxylate derivatives by using Sonogashira cross-coupling.

Quinoxalines are an essential class of heterocyclic compounds having nitrogen atoms because of their important biological activities alongside pharmacological activities [188]. The Sonogashira coupling is a significant reaction utilized for carbon-carbon bond formation [189,190]. However, multi-component reactions (MCRs) are strong and prevailing chemical ways for the formation of important organic compounds [191,192,193]. To synthesize new 3-(3-(aminoquinoxalin-2-yl) prop-2-yn-1-yl carboxylates, Abbaspour et al. used a multi-component, copper-free Sonogashira coupling. In this reaction, carboxylic acids react with propargyl bromide and various amine-substituted chloroquinoxalines while using a Pd catalyst. The antibacterial properties of each newly synthesized compound were examined. 3-(3-(diethylamino)quinoxalin-2-yl)prop-2-yn-1-yl carboxylates **181** were generated via the multicomponent reaction of chloroquinoxalines amines **178**, carboxylic acids **179**, and propargylbromide **180**, which was catalyzed by Pd(PPh_3_)_2_Cl_2_ in DMF in the presence of Et_3_N and Cs_2_CO_3_. Compounds **181a**–**g** against *M. luteus* while **181b**, **181c**, and **181g** against *P. aeruginosa* showed superior antibacterial activity when compared to the other derivatives, according to the MIC and MBC values (Scheme 33) [194].

**Scheme 34:**
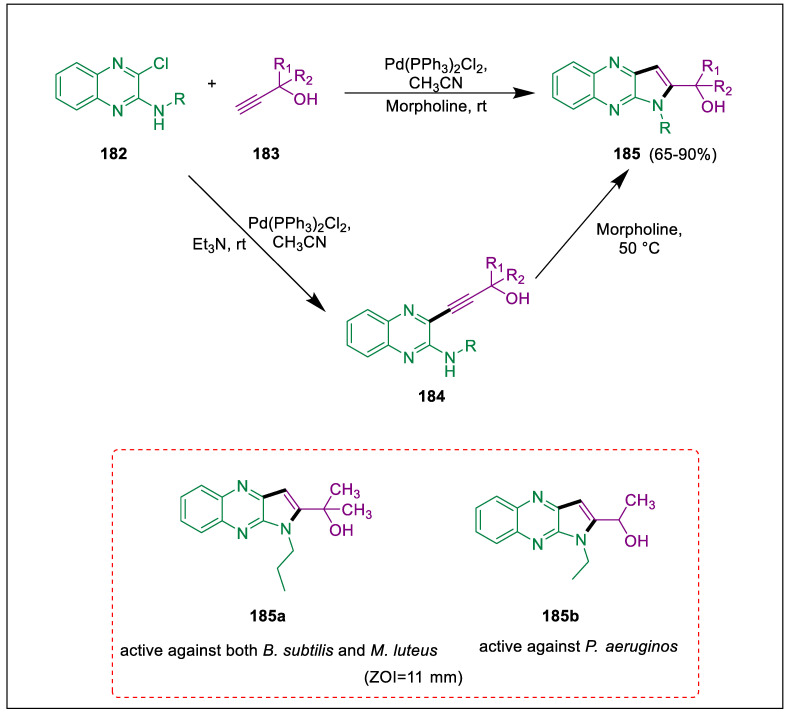
Synthesis of pyrrolo quinoxaline derivatives by using Sonogashira cross-coupling.

Products from the Sonogashira coupling are widely employed in the production of heterocyclic compounds, agrochemicals, and medicines [195]. The benzoheterocycle quinoxaline and its derivatives constitute an important class with a broad spectrum of biological action. Pyrroloquinoxalines act as bioactive compounds [196]. The synthesis of disubstituted pyrrolo quinoxalines has been described by Cacchi and colleagues. By reacting *N*-alkyl chloroquinoxaline amines with propargylic alcohols in the absence of copper but in the presence of Pd(PPh_3_)_2_Cl_2_, Fakharian et al. produced alkanol-substituted pyrrolo quinoxalines. Additionally, the three bacterial strains were tested against the produced pyrroloquinoxaline derivatives [197]. *N*-alkyl-3-chloroquinoxaline-4-amines **182** synthesized [198,199]. They were followed by a reaction at room temperature with propargylic alcohols **183**, catalytic quantities of Pd(PPh_3_)_2_Cl_2_, morpholine, and MeCN to generate 2-alkanol substituted pyrrolo[2,3-*b*]quinoxalines **185**. However, when morpholine was not employed in the first step, intermediate **184** was produced, which in the presence of morpholine and CH_3_CN synthesized derivatives **185**. While compounds **185** were assessed for their antibacterial activity at a concentration of 1000 μg/mL in methyl sulfoxide. Compound **185a** demonstrated antibacterial properties against both *B. subtilis* and *M. luteus,* and compound **185b** was active against *P. aeruginos* better than penicillin (Scheme 34) [197].

**Scheme 35:**
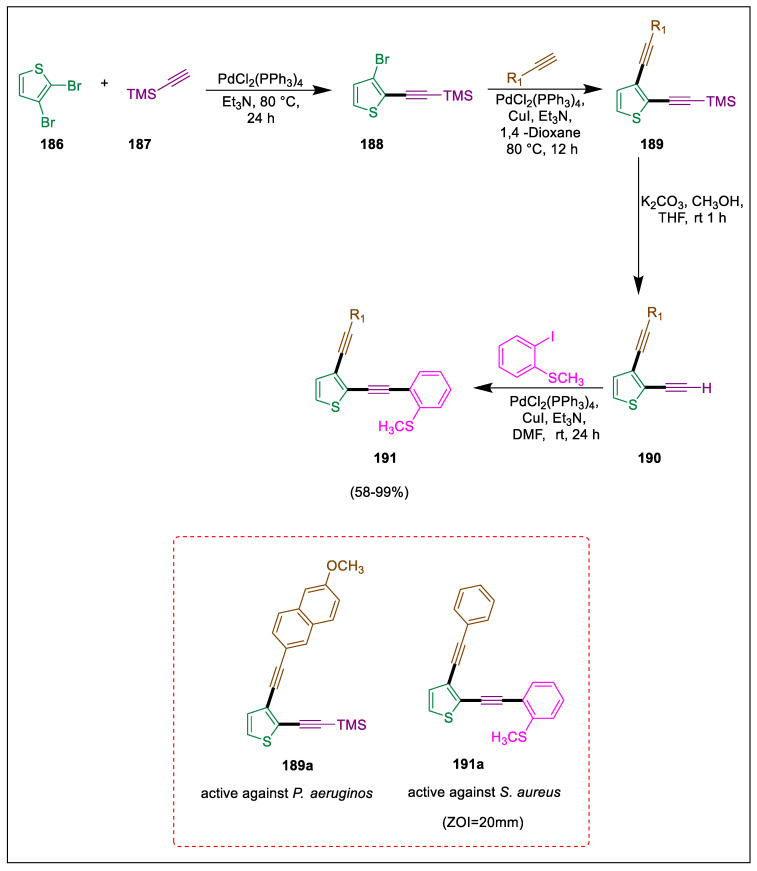
Synthesis of thiophene derivatives by using Sonogashira cross-coupling.

Because thiophene and its derivatives have so many different biological actions, they have been studied as an essential class of heterocyclic chemicals [200]. Thieno[*a*]dibenzothiophene derivatives are normally synthesized by Pd as a catalyst in cycloaddition reactions between alkynes and halo-benzothiophenes [201]. A range of highly substituted thieno dibenzothiophenes were produced by Konus et al. via a cascade electrophilic cyclization procedure. The reaction allowed a range of molecules to be examined for antimicrobial and antifungal activities. The reaction between bromothiophene **186** and ethynyl(trimethyl)silane **187** by using Sonogashira coupling to prepare ((3-bromothiophen-2-yl)ethynyl)trimethylsilane **188**, which again undergoes Sonogashira coupling to obtain the desired compound silane **189**, which undergoes desilation for the synthesize of 2-ethynyl-3-(aryl/alkyl ethynyl)thiophenes **190**, which react with 2-iodothioanisol by Sonogashira reaction to form thiophene derivatives **191**. Compound **191a** demonstrated antibacterial activity against *S. aureus*, while compound **189a** was the only one to show some degree of activity against *P. aeruginosa* (Scheme 35) [202].

**Scheme 36:**
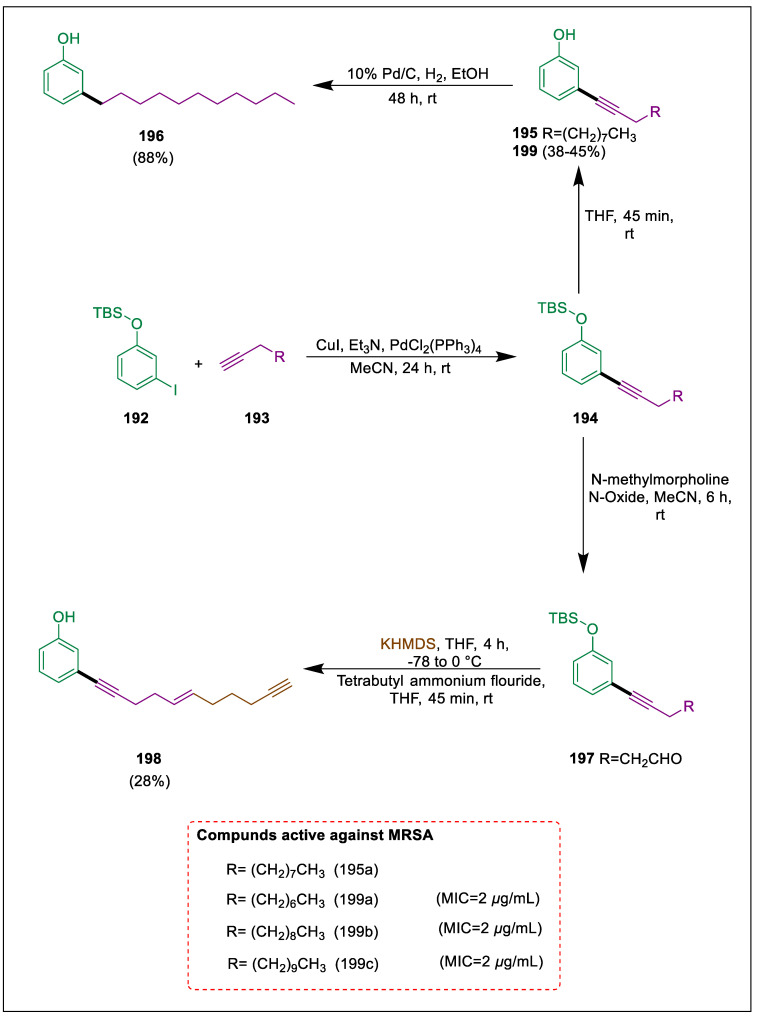
Synthesis of anaephene derivatives by using Sonogashira cross-coupling.

Multidrug-resistant bacteria pose a serious risk, necessitating the synthesis of new antibiotics [203]. Natural antibacterial substances provide great starting points for the development of innovative treatments to identify drug-resistant microorganisms. Overall Synthesis of molecules that occur naturally offers a chance to overcome these problems [204]. Kukla et al. produced and studied eighteen analogs of the natural product anaephene. It was discovered that the antibacterial activity of these analogs against MRSA and MSSA varied. Compound **199** discovered that, in comparison to anaphase B, an internal alkyne with no extra unsaturations in the alkyl chain increases antibacterial efficacy against MRSA. Compounds **194** are obtained by Sonogashira coupling of *tert*-butyl-(3-iodanylphenoxy)-dimethyl-silane **192** with either 1-undecyne or pent-4-yn-1-ol **193**. After treating compound **194a** with THF to eliminate the TBS-protecting group, alcohol **195** was produced. Under hydrogenation conditions, compound **195** yields alcohol derivatives **196**, and other derivatives, such as **199**, were also synthesized but do not undergo hydrogenation. Alcohol **194b** oxidized to aldehyde **197**, then underwent sulfone-mediated Julia-Kocienski olefination, and finally, TBAF deprotection produced compound **198**. Compound **195a** is more potent than compound **193** with MIC values of 2 μg/mL, although compound **198a** is equivalent to the natural product anaphase B. Compounds **199a**–**c** exhibit action against *S. aureus* that is resistant to methicillin (Scheme 36) [205].

**Scheme 37:**
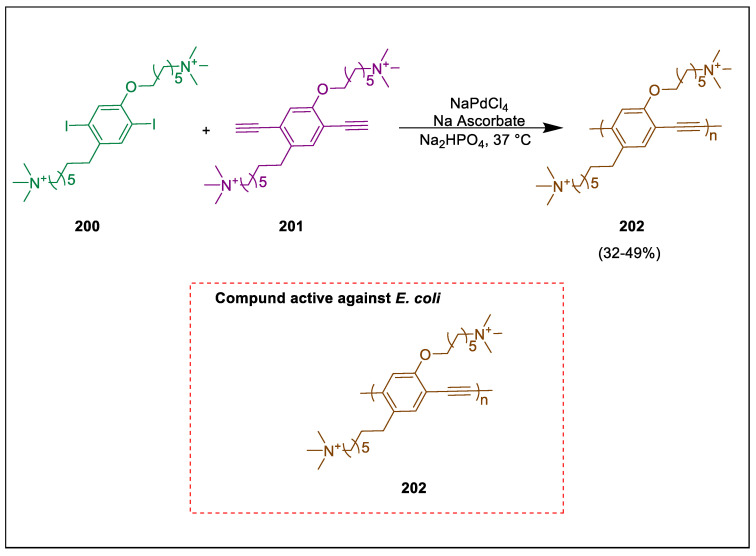
Synthesis of photoactive polyphenylene ethylene (PPE) derivatives by using Sonogashira polymerization.

Engineering the surface of cells is crucial for controlling how cells behave, including shielding them from hostile surroundings [206,207] and improving biosynthesis efficiency [208,209]. Numerous functional materials are used as coating materials on the surface of cells, including metal/semiconductor nanoparticles [210,211], polymers [212,213], and metal complexes [214]. Live cells synthesized by Pd-mediated reactions have recently been subjected to the in-situ polymerization technique. Using a cell-generated bio-palladium catalyst and a Sonogashira polymerization process, Qi et al. formed photoactive polyphenylene ethylene (PPE) on the cell surface. The in-situ generated PPE has excellent light-harvest capacity and blue fluorescence on the surfaces of *E. coli*. Additionally, PPE shows increased antibacterial activity against *E. coli*. Cationic 1,4-bis(oxyhexamethylene-trimethylammonium bromide)-2,5-diiodobenzene **200** and 1,4-bis(oxyhexamethylene-trimethylammonium bromide)-2,5-diethynylbenzene **201** choose as monomers and Sonogashira polymerizations take place in aqueous solution, catalyzed by the in-situ produced surface bio-palladium catalysts to form photoactive polyphenyleneethynylene (PPE) **202**. In-situ synthesized photoactive polyphenyleneethynylene (PPE) **202** exhibits good antibacterial capacity (Scheme 37) [215].

**Scheme 38:**
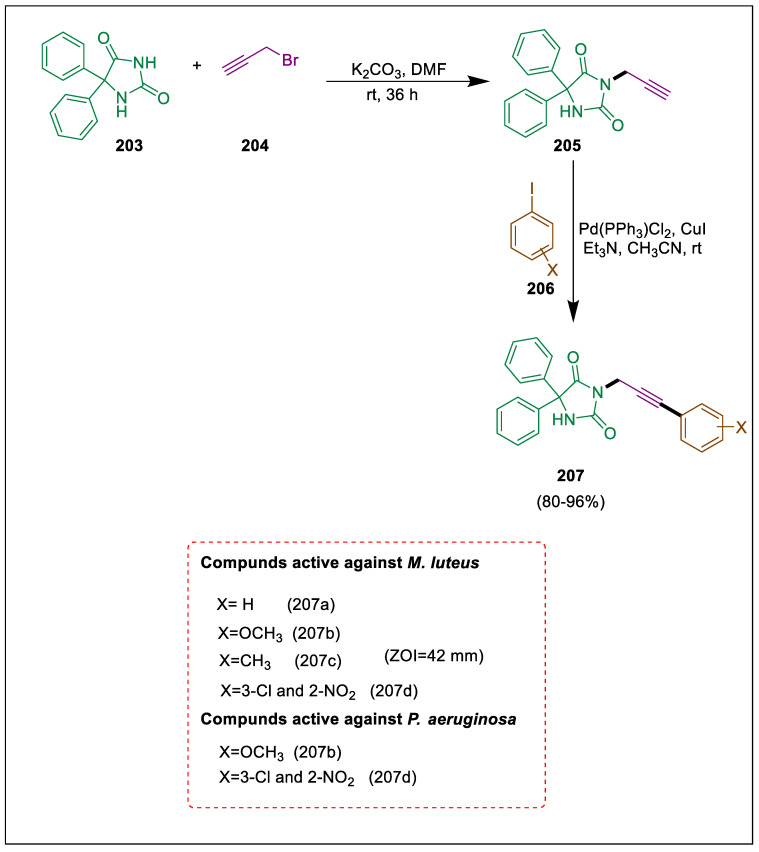
Synthesis of dione derivatives by using Sonogashira cross-coupling.

The powerful and essential approach to forming C-C bonds and aryl-alkynes is known as Sonogashira coupling [216,217], and it is used to make biologically active compounds in the pharmaceutical industry [218]. Important nitrogen heterocyclic compounds in the organic synthesis and pharmaceutical industry are hydantoins. Important nitrogen heterocyclic molecules in organic synthesis and medicinal chemistry are hydantoins. Compounds **207** were synthesized by Keivanloo et al. by reacting diphenyl imidazolidine 2,4-dione with ArI_2_ in CH_3_CN at normal temperature with a palladium catalyst. *Pseudomonas aeruginosa* and *Micrococcus luteus* were the two bacterial strains against which all produced compounds were tested. Diphenylimidazolidine-2,4-dione **203** was produced as a starting material by the reaction of urea and benzil in alkaline EtOH. This compound then reacted with 3-bromo-prop-1-yne **204** in the presence of potassium carbonate in DMF as a solvent to yield 5,5-diphenyl-3-(prop-2-yn-1-yl) imidazolidine-2,4-dione **205**, which was coupled with ArI_2_ **206** in the presence of a palladium catalyst and CuI in CH_3_CN at room temperature to synthesize derivatives **207**. Compounds **207a**–**d** exhibit superior inhibitory activity against *M. luteus*, while compounds **207b** and **207d** demonstrate superior inhibitory activity against *P. aeruginosa* (Scheme 38) [219].

### 2.3. Stille Cross-Coupling

**Scheme 39:**
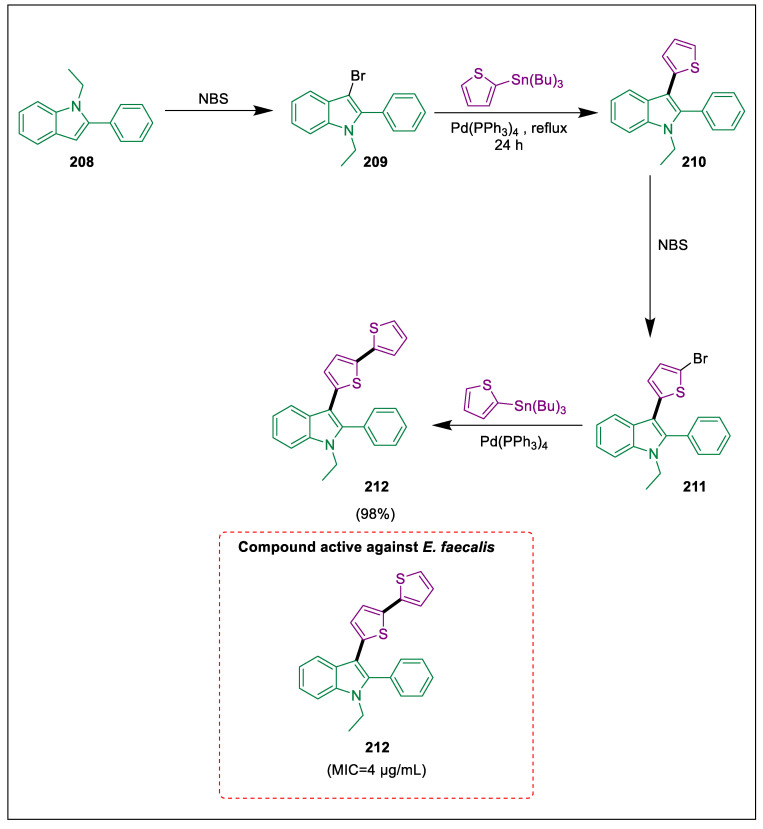
Synthesis of indole derivatives via Pd-catalyzed Stille cross-coupling.

The indole and patterns are frequently observed in most compounds with biological activity, including medications and alkaloids [220]. Numerous significant medical properties have been attributed to indole derivatives, including anti-inflammatory [221], antiallergic, anti-viral [222], anti-tumor [223], antimicrobial [224], antihypertensive [225], and antioxidant activities [226]. Konus et al. conducted electrophilic substitution reactions and Pd-catalyzed Stille coupling to produce novel indole derivatives containing mono- and di-thiophene groups **211** and **212**. Compound **211** exhibited no notable cytotoxic, antioxidant, or antimicrobial properties. In contrast, compound **212** demonstrated substantial reducing and exceptionally potent antibacterial activity. Compound **212** is also a potentially effective malignancy therapeutic agent. By treating 1-ethyl-2-phenylindole1 **208** with NBS to form 3-bromo-1-ethyl-1*H*-indole **209**, which undergoes an electrophilic aromatic substitution reaction with 2-(tributylstannyl) thiophene in the presence of a Pd catalyst to form compound **210**. Following the characterization process, a second electrophilic aromatic substitution reaction was conducted to produce 1-phenyl-1*H*-indole **211**, which had a 92% yield. In the end, 3-([2,2′-bithiophen]-5-yl)-1-ethyl-2-phenyl-1*H*-indole **212** was synthesized using a Stille coupling reaction between 2-(tributylstannyl) thiophene and 1-(5-bromothiophen-2-yl)-1-ethyl-2-phenyl-1*H*-indole **211** in the presence of a Pd catalyst while under prolonged reflux. The antimicrobial activity of compound **212** (250 μg) towards three gram-positive bacteria was found. However, it exhibited exceptionally potent antibacterial activity against *E. faecalis* ATCC 29212, with a MIC of 4 μg/mL (Scheme 39) [227].

**Scheme 40:**
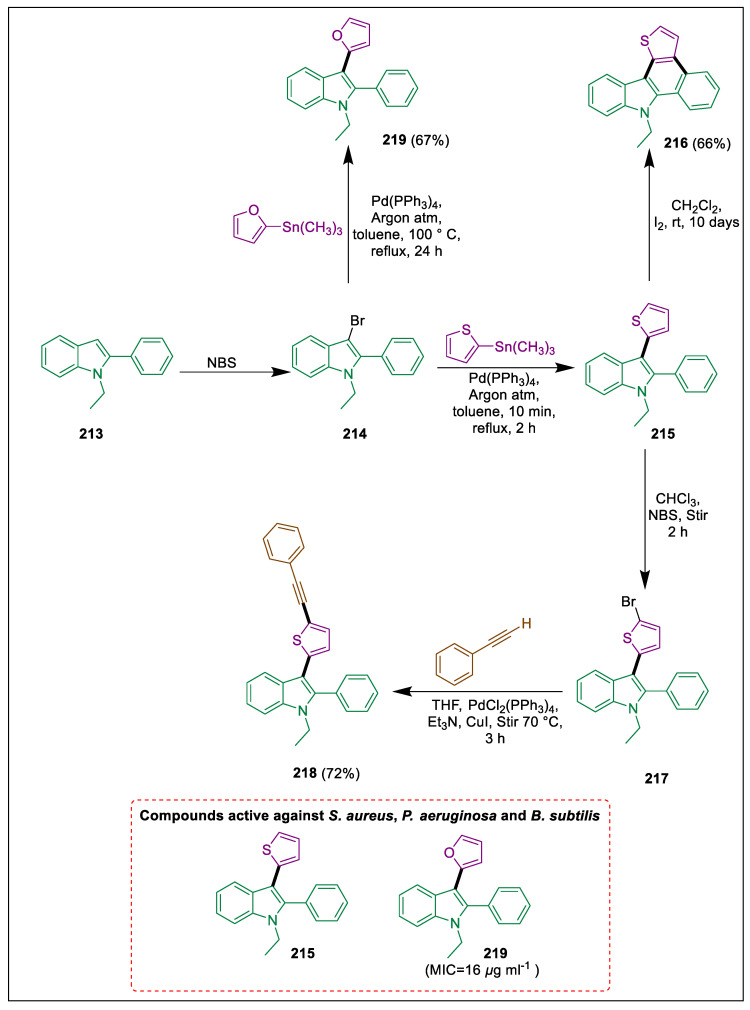
Synthesis of indole derivatives by using Stille and Sonogashira cross-coupling.

Important biological actions are displayed by indole and indole-based compounds [228,229]. Indoles have the potential to function as a crucial structural class in synthetic medications, including sumatriptan, indomethacin, pindolol, indolmycin, and reserpine [230]. The prevalence of multidrug-resistant microbial infections has increased substantially, posing an additional risk to human health. Konus et al. used a Pd catalyst in cross-coupling reactions and iodocyclization reactions to synthesize different derivatives (**215**–**219**). Moreover, compounds **215** and **219** had antifungal and antibacterial action, suggesting they were superior medications. After undergoing an electrophilic substitution reaction, indole **213** was converted to 3-bromo-1-ethyl-2-phenyl-1*H*-indole **214**. This allowed it to react with tributylthiophen-2-ylstannane in the presence of palladium as a catalyst to form compound **215**, which with bromide undergoes Stille coupling, resulting in 3-(5-bromothiophen-2-yl)-1-ethyl-2-phenyl-1*H*-indole **216**. Conversely, a novel synthetic method was used to synthesize polyheteroaromatic compound **217** in the presence of I_2_ by using compound **215**. After that, compound **218** was formed through a Sonogashira coupling reaction with terminal alkyne. Next, compound **214** underwent the Stille coupling procedure, and furanyl-substituted indole derivative **219** was formed. In terms of inhibitory zones against *S. aureus* ATCC 25,923 (25 mm), *P. aeruginosa* ATCC 27,853 (25 mm), *B. subtilis* ATCC 6633 (19.5 mm), and compound **219** was the most powerful. Subsequently, compound **215** also showed strong antimicrobial activity (Scheme 40) [231].

### 2.4. Buchwald-Hartwig Coupling

**Scheme 41:**
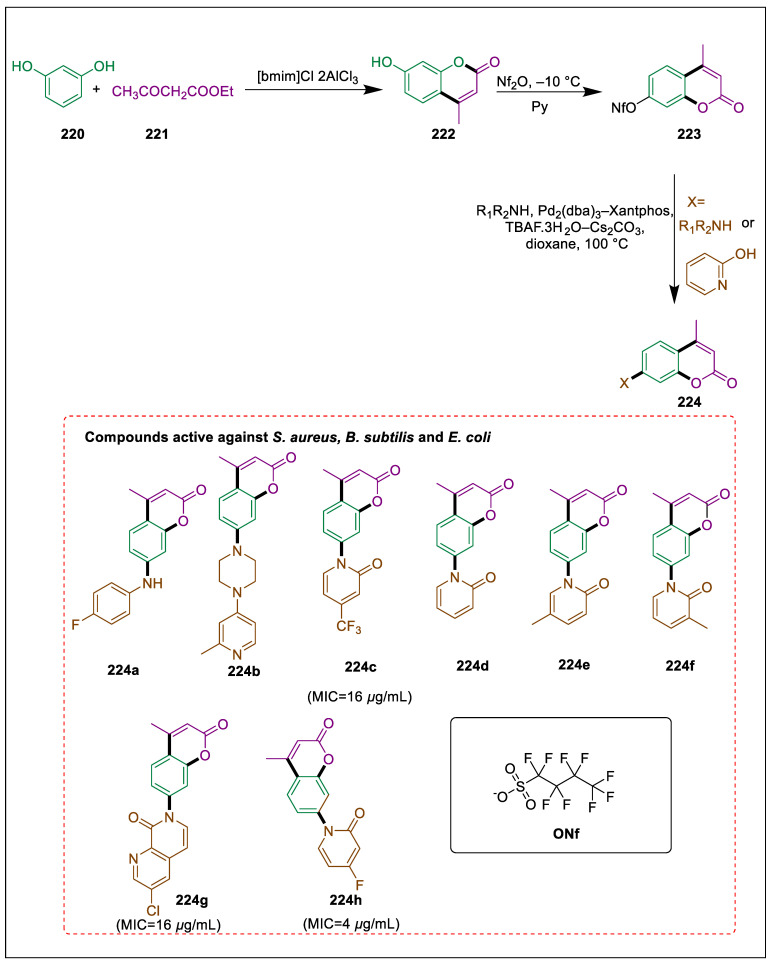
Synthesis of amino/amido coumarins by using Buchwald-Hartwig coupling.

Numerous uses in therapy have been attributed to natural and synthetic varieties of coumarin-containing amines and amides, which are reportedly potent antibacterial [232], anti-inflammatory [233], and antiviral agents [234], and show various applications in medicinal chemistry. Certain pharmaceuticals available for purchase contain the coumarin moiety [235]. Our investigation into the pharmacological potential of coumarins coupled with various amides and amines was motivated by the preceding findings of coumarins and amines. Joy et al. synthesized methyl amido/amino coumarins by coupling a variety of coumarins with amides and amines via a palladium-catalyzed Buchwald crossing reaction. There are intentions to investigate the antioxidant and antimicrobial characteristics of the synthesized methyl amino/amido coumarins. The antibacterial capacity of molecule **224h** was like that of the standard ciprofloxacin. The hydroxy coumarin **222** was produced through an adapted Pechmann cyclization of resorcinol **220** with ethyl acetoacetate **221**. This coumarin was subsequently converted to the nonaflate **223**, which underwent a Buchwald-Hartwig cross-coupling reaction with different amines and pyridines at –10 °C to synthesize methyl amino/amido coumarins **224**. The catalyst for this coupling was Pd_2_(dba)_3_, and the ligands consisted of Xanthos. Compounds **224a**–**h** exhibited comparable and favorable activity to ciprofloxacin. In contrast, compounds **224g** and **224h** demonstrated potent activity towards every bacterial strain (*S. aureus*, *B. subtilis*, and *E. coli*). The antibacterial effect of compounds **224o**–**u** (amides) was superior to that of **224a**–**t** (amines) (Scheme 41) [236].

**Scheme 42:**
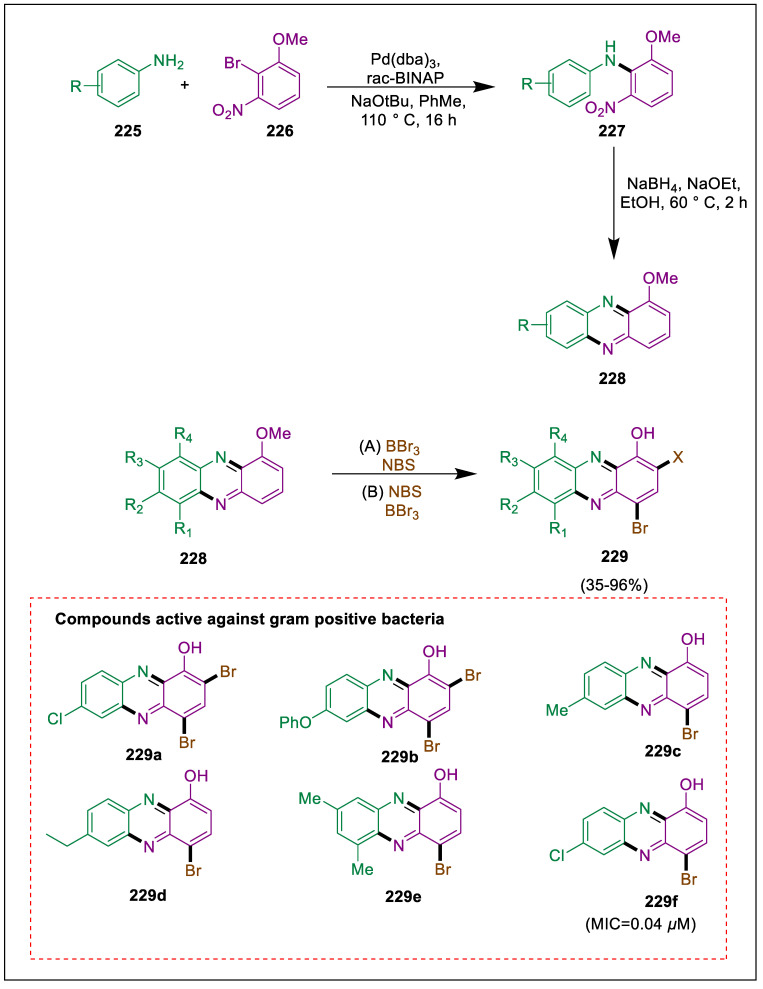
Synthesis of phenazine derivatives by using Buchwald-Hartwig reaction.

To stop the proliferation of bacteria that are resistant to antibiotics, new antibacterial medications with unique modes of action must be developed [237]. Unfortunately, because there are currently no biofilm-eradicating medicines available, biofilm-associated illnesses remain an unsolved clinical concern. Phenazine 2-bromo-1-hydroxyphenazine, one analog that has been halogenated (HP), exhibits significant antibacterial and biofilm-destructive properties. Halogenated phenazine (HP) analogs that exhibit biofilm-eradicating activity against priority pathogens were identified by Garrison et al. The synthesis of 1-methylphenazine scaffolds via Buchwald-Hartwig cross-coupling and reductive cyclization facilitated the rapid identification of strong HPs that destroy biofilms. By employing 6 mol% Pd_2_(dba_3_) and 18 mol% (±)rac-BINAP as a ligand, anilines **225** were coupled with **226** to produce 2-series diarylamines **227** with an average yield of 71% and inverted 3-series with an average yield of 25%. The 2-series diarylamines underwent a smooth two-step Buchwald-Hartwig/reductive cyclization, yielding 1-methoxyphenazines **228**. However, the 3-series diarylamines did not yield any desired 1-1-methoxyphenazine products. Thirteen of these intermediates were then demethylated using boron tribromide to yield 1-hydroxyphenazine, and its dibromination produced HP target structures analogs **229**, while bromination at position 4 also produced other derivatives **229**. For instance, halogenated phenazines **229a** and **229b** report MIC activity of less than 0.1μM. The MICs of 6.25–50 μM for monohalogenated HPs **229c**–**f** were merely fair to moderately active against gram-positive bacteria (Scheme 42) [238].

**Scheme 43:**
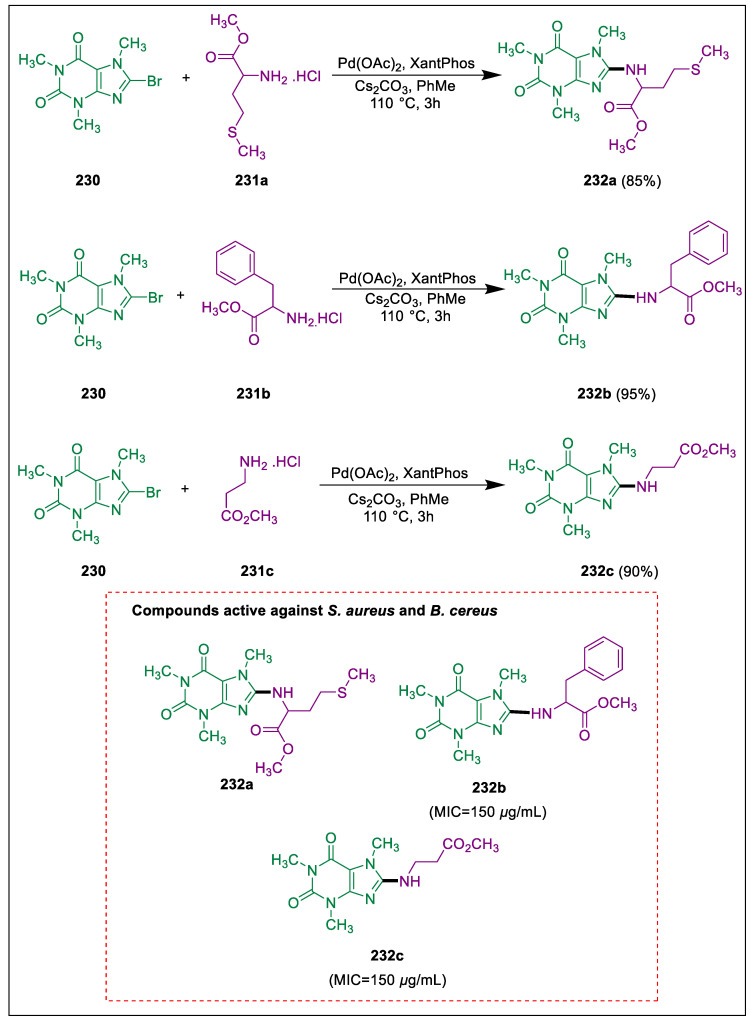
Synthesis of xanthine derivatives by using Buchwald-Hartwig reaction.

Caffeine, a naturally occurring 1,3,7-trimethyl xanthine, has biological action and is employed as a biostimulant in food and medicine [239,240]. As bactericidal agents, caffeine and its derivatives that have been 8-alkoxy substitution are recognized [241,242,243]. Utilizing cross-coupling reactions to introduce substituents into the xanthine skeleton at the C-8 position is a crucial technique. Reshetnikov et al. cross-coupled bromocaffeine with *α*-, *β*-, or *ω*-amino-acid of methyl or *t*-butyl ester hydrochlorides in toluene in the presence of a Pd catalyst and cesium carbonate with microwave activation, which results in the synthesis of xanthine derivatives containing amino-acid fragments in the C-8 position and study their antibacterial activities. Bromo caffeine **230** reacts with methyl or *t*-butyl ester hydrochlorides of *α*, *β*, or *ω* amino acids **231a**–**c** in dry toluene with microwave heating to synthesize synthetic xanthine derivatives **232a**–**c**, including amino-acid fragments, in the presence of a Pd catalyst and Cs_2_CO_3_. Compounds **232a**–**c** demonstrated decreased minimum inhibitory concentrations (MIC) for *B. cereus* growth suppression and were active at 150 ± 25 μg/mL. These compounds inhibited the growth of *S. aureus* at concentrations greater than 500 μg/mL (Scheme 43) [244].

### 2.5. Heck Reaction

**Scheme 44:**
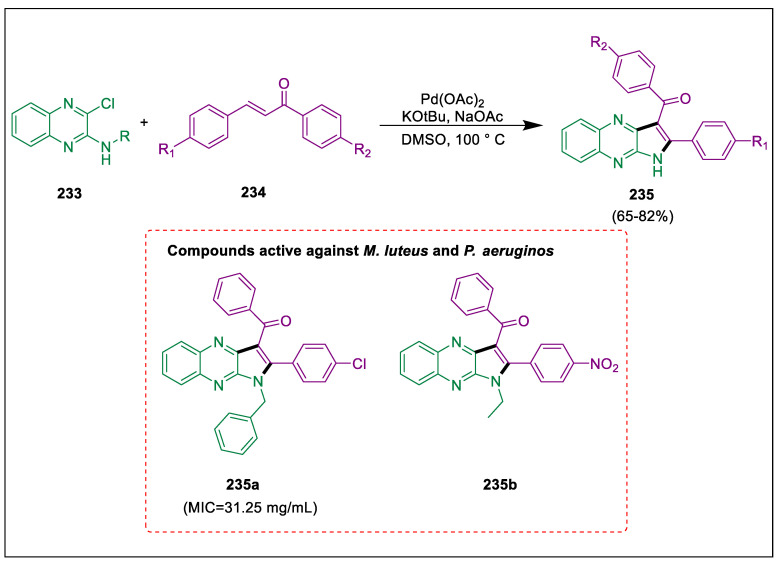
Synthesis of quinoxaline derivatives via Heck reaction catalyzed by palladium.

Quinoxaline derivatives have highly intriguing biological characteristics [245,246]. Cross-coupling reactions of aryl halides with organometallic reagents catalyzed by palladium to produce C-C bonds, which are utilized to synthesize medicinal and agrochemical compounds [247]. Because of the biological and pharmacological characteristics of pyrroloquinoxalines, several straightforward techniques have been established for their production. Seidani et al. synthesized quinoxaline derivatives by reacting *N*-alkyl/benzyl-3-chloroquinoxaline-2-amines with chalcones catalyzed by palladium with the addition of potassium *tert*-butoxide as the base in DMSO. The MIC and MBC values indicated these compounds might be employed in future research for additional antibiotic synthesis. The intermolecular Heck reactions were employed to synthesize trisubstituted pyrrole quinoxalines **235** from *N*-alkyl/benzyl-3-chloroquinoxaline-2-amines **233** and chalcones **234** in the presence of Pd catalyst, KOtBu, NaOAc, and DMSO. Compounds **235a** and **235b** showed stronger anti-bacterial activities against *M. luteus* and *P. aeruginos* than the other derivatives when both the MIC and MBC values were taken into consideration. Anti-bacterial activity of **235a** was comparable to tetracycline with strong inhibition (Scheme 44) [248].

**Scheme 45:**
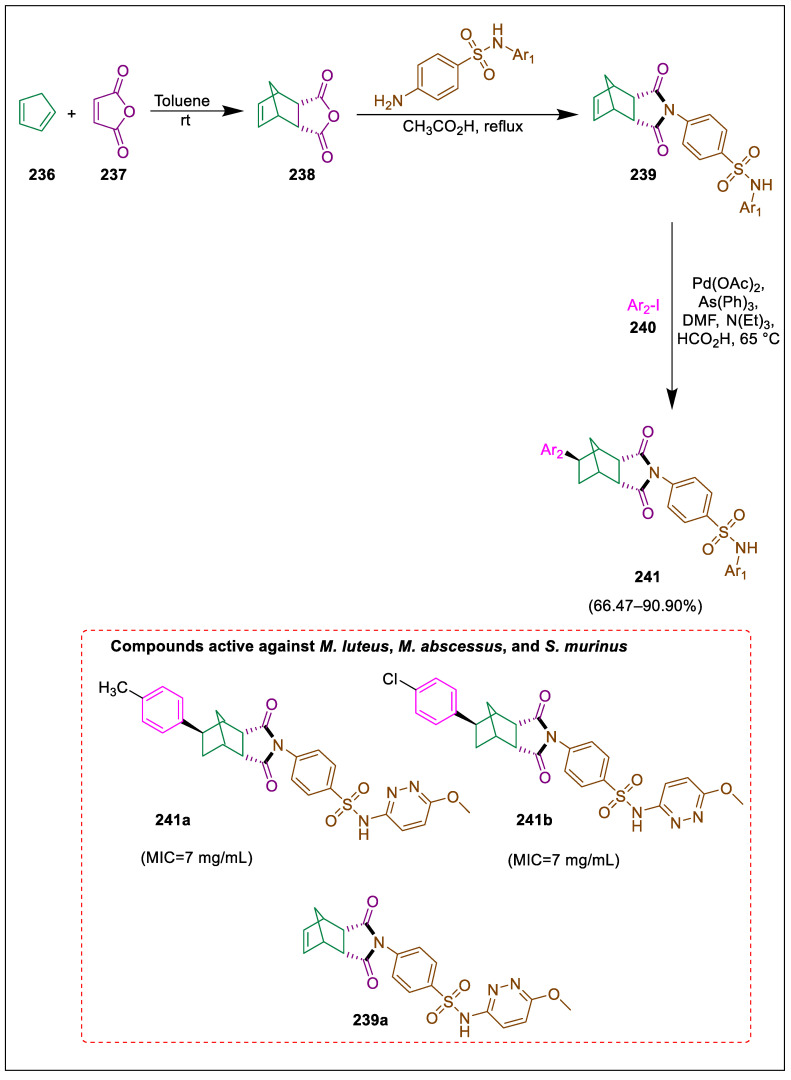
Synthesis of sulfa drug-based norbornenylimide derivatives by using reductive Heck reaction.

Numerous pharmacologically active substances have sulfonamide moiety. Important pharmacophores with a broad spectrum of bioactivity are cyclic imides. The bicyclic structure of norbornane [bicyclo(2.2.1) heptane] is bridged, and its derivatives show some biological characteristics [249,250]. Synthesis of medicines and agrochemicals is possible by using the Heck reaction. By reacting endo-endic anhydride with sulfa medications and then reductive Heck reactions of these products, Bagdatli and Cil produced novel sulfa drug-substituted norbornyl imides as potential bioactive scaffolds. The antibacterial activity of all eight produced compounds, aryl aryl-substituted norbornyl imides, and two sulfa drug-based norbornenyl imides was assessed against nine different pathogens. Furan-2,5-dione **236** and freshly distilled cyclopenta-1,3-diene **237** react to form endogenic anhydride **238** in the presence of dry toluene. This endogenous anhydride then reacted with sulfa drugs, specifically sulfamethoxypyridazine and pyridazine in acetic acid, to form sulfa drug-based norbornenylimides **239**, which undergo reductive Heck reactions with aryl iodide **240**, yielding sulfa-based norbornenylimide derivatives **241**. For *M. luteus, M. abscessus*, and *S. murinus*, MIC results from samples **241a** and **241b** were superior to those from other investigated samples. Among the actinobacteria under study, **239a** had a MIC of 7.0–14.0 mg/mL and produced better results than other compounds against bacterial strains (Scheme 45) [251].

### 2.6. 1,4-Addition to Dienes

**Scheme 46:**
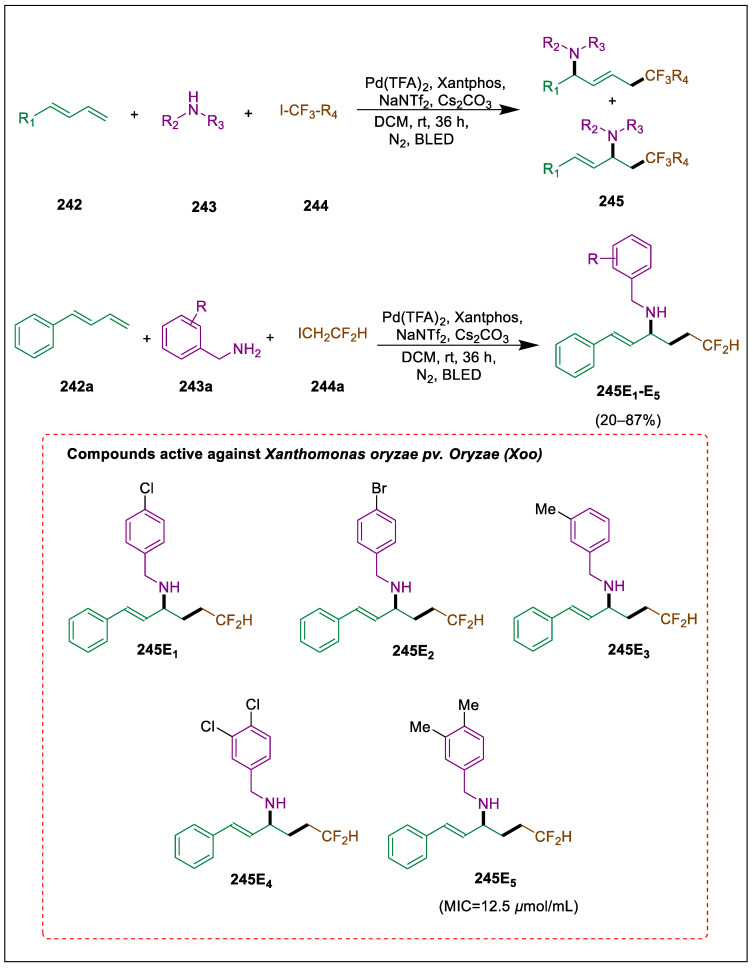
Synthesis of amine derivatives by using 1,4-addition to dienes.

Bacterial leaf blight (BLB) is a serious global disease caused by the bacterium *Xanthomonas oryzae pv. Oryzae (Xoo)* that severely decreases rice productivity. Implementing pharmacological or biological agents is of the utmost importance to mitigate the detrimental effects of the disease [252,253,254,255]. It is extremely advantageous to incorporate fluoroalkyl motifs into organic frameworks and their application in pharmaceutical compounds. Through the coupling of 1,3-dienes, amines, and fluoroalkyl iodides, Shi et al. produced a series of photoinduced selective 1,3-diene fluoroalkyl amination derivatives. The bactericidal activity of all the synthesized compounds against Xoo was assessed. Compound E14 among them exhibited notable efficacy against *Xanthomonas oryzae pv. Oryzae (Xoo)*. In the presence of a palladium catalyst with xantphos as ligand, Cs_2_CO_3_ as a base, and DCM, 1,3-dienes **242**, amines **243**, and fluoroalkyl iodides **244** combine to form 1,3-diene-selective fluoroalkyl amination derivatives **245**. By using fluoroalkyl iodide **244a**, 1,3-diene **242a**, and amines **243a**, we synthesized the compounds listed in the title, which are amine derivatives, **245E_1_**–**E_5_**. Compounds **245E_1_**–**E_5_** displayed outstanding antibacterial activity against Xoo, with **245E_5_** having a MIC of 12.5 μmol/mL (Scheme 46) [256].

## 3. Copper-Catalyzed Synthesis

Cross-coupling reactions are effective methods for forming carbon-carbon (C-C) bonds and are frequently employed in the synthesis of a variety of chemicals [257,258,259]. Pd complexes are usually the catalysts for these reactions. Much work has recently been put into developing Cu as a Pd substitute for these kinds of processes [260]. Concerns over the long-term sustainability of cross-couplings, mainly related to the high cost and poor natural availability of Pd, are the driving force behind efforts to develop Cu-based catalytic procedures.

Even though it can be challenging to accomplish Cu-catalyzed cross-couplings, Cu-salts are, however, quite effective in forming C-C bonds in a variety of procedures. Excellent yields and milder reaction conditions are two benefits of using copper catalysts. Among other transition-metal-mediated reactions to form carbon-carbon and carbon-heteroatom bonds, copper catalysis is used in Ullmann reactions, Diels-Alder reactions, ring expansions, and a notable variation of the Huisgen 1,3-dipolar cycloaddition that uses a Cu(I) catalyst that was independently developed by Meldal and Sharpless. Copper(I)-catalyzed Azide-Alkyne Cycloaddition (CuAAC) is a reaction that forms a triazole by combining an azide and a terminal alkyne. The production of biological molecules and medicinal materials depends extensively on copper’s capability to catalyze carbon-carbon and carbon-heteroatom bonds [261].

### 3.1. Click Reaction

**Scheme 47:**
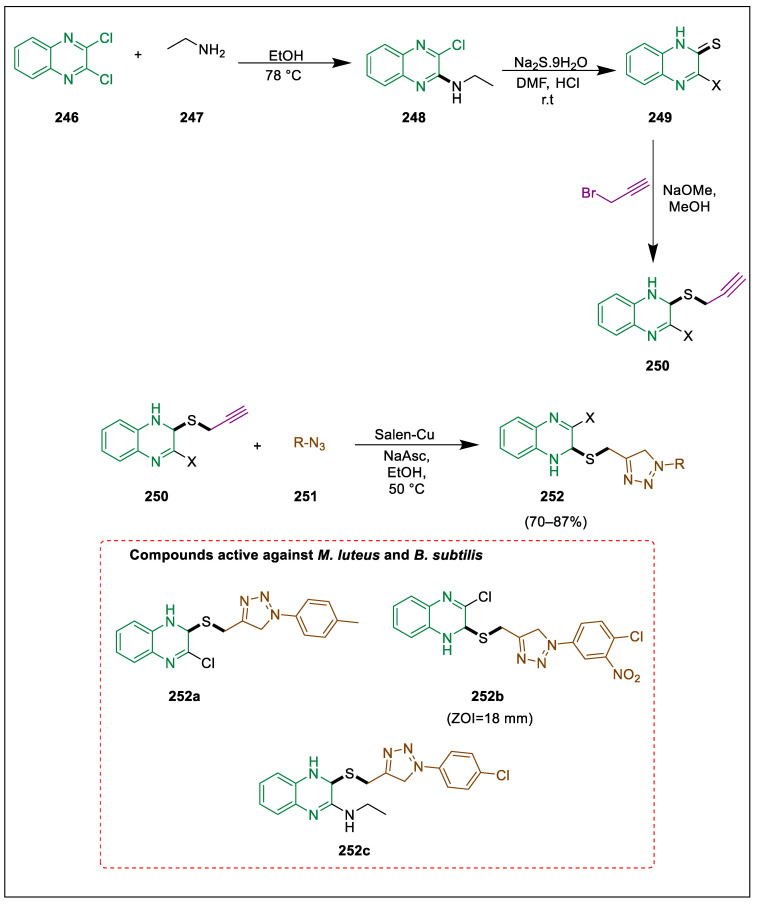
Synthesis of triazole-linked thioquinoxaline derivatives via Cu salen complex-catalyzed click reaction.

Varieties of groups comprise a quinoxaline, which shows biological activity [262]. Significant heterocyclic compounds, triazoles, have applications in organic chemistry, medicinal chemistry, and drug discovery [263,264,265,266]. Transition metal complexes derived from Schiff bases have valuable catalytic transformations [267,268]. Therefore, triazoles-based 3-substituted thioquinoxalines were synthesized using Cu(II) salen complex. The Cu(II) salen complex catalyzed a click reaction between 2-propargythioquinoxalines and aryl azides; the resulting triazole-linked thioquinoxaline derivatives were formed by Keivanloo et al. The in vitro antibacterial activities of these compounds indicated that *Bacillus subtilis* and *Micrococcus luteus* bacteria are susceptible to the effects of compounds **252a**, **252b**, and **252c**. By using the salen Cu (II) complex as a catalyst, the loading of the hazardous copper species was decreased, and the reactivity rate was increased. The reaction between dichloroquinoxaline **246** and morpholine **247** produced chloroquinoxaline amines **248** when combined with Na_2_S.9H_2_O in dimethyl sulfoxide (DMF) at ambient temperature. Subsequently, substituted thioquinoxalines **249** were made, which reacted with propargyl bromide in the presence of sodium methoxide in methanol, resulting in the formation of propargyl thioquinoxalines **250** in a quantitative yield, which reacted with azide **251** in ethanol, then added the salen-Cu(II) complex and sodium ascorbate, and stirred the mixture at 50 °C for 3h to obtain the pure product **252** obtained by crystallization from ethanol. By using a good diffusion method, the antibacterial activities of the newly synthesized molecules against *M. bacteria* (DSM 6887), *P. aeruginosa* (ATCC 27853), and *M. luteus* (ATCC 4698) were assessed. Antibacterial properties have been observed in molecules **252a**–**c** against *M. luteus* and *B. subtilis* (Scheme 47) [269].

### 3.2. Ullman Cross-Coupling

**Scheme 48:**
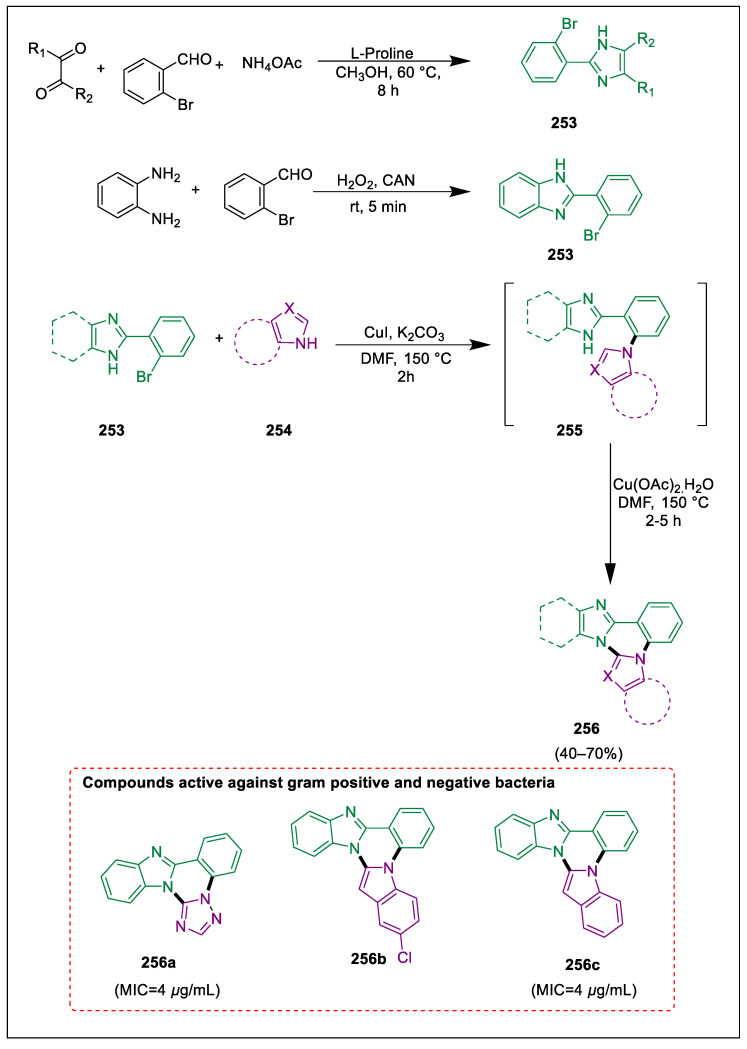
Synthesis of imidazo/benzimidazo quinazolines by using Ullman cross coupling.

Because there are no effective antibiotics for microbial diseases, they have become a global health concern. Heterocyclic compounds nitrogen-containing are the most prevalent and exhibit both biochemical and economic uses [270]. Hoemann et al. discovered that 2-(1*H*-indol-3-yl) quinolines were efficacious towards *S. aureus*, which resists methicillin [271]. Kung et al. found that compounds having quinazoline possess potent antibacterial properties [272]. Nandwana et al. performed a Cu-catalyzed Ullmann-type C-N coupling to synthesize fused quinazolines, followed by an intramolecular cross-dehydrogenative coupled process. The in vitro antibacterial activity of these compounds was evaluated against three gram-negative bacteria and two gram-positive bacteria. Compared to the beneficial control ciprofloxacin, the compounds with the lowest inhibitory concentrations against all bacterial strains examined were **256a**, **256b**, and **256c**. A series of bromoaryl imidazoles **253** were produced by reacting benzyl and a related dicarbonyl compound, 2-bromoaryl aldehyde, and NH_4_OAc in the presence of L-proline/MeOH. Similarly, one of the derivatives 2-(2-bromophenyl)-1*H*-benzo[*d*]imidazole **253** was produced by reacting a bromoaryl aldehyde with diaminobenzene in the presence of H_2_O_2_/CAN. Aryl modification at the C-4 and C-5 positions of compound **253** when reacted with various azoles **254** in the presence of CuI and potassium carbonate in DMF at 150 °C and produced intermediates **255** followed by Ullmann-type coupling catalyzed by Cu(OAc)_2_·H_2_O resulted in the fused imidazo/benzimidazo quinazolines **256**. Using ciprofloxacin as a standard drug, an antibacterial evaluation of imidazole/benzimidazole quinazoline derivatives was conducted against a panel of three gram-negative and two gram-positive strains. Compounds **256a**, **256b**, and **256c** were determined to exhibit the highest efficacy and antibacterial activity (Scheme 48) [273].

### 3.3. Chan-Lam Cross-Coupling

**Scheme 49:**
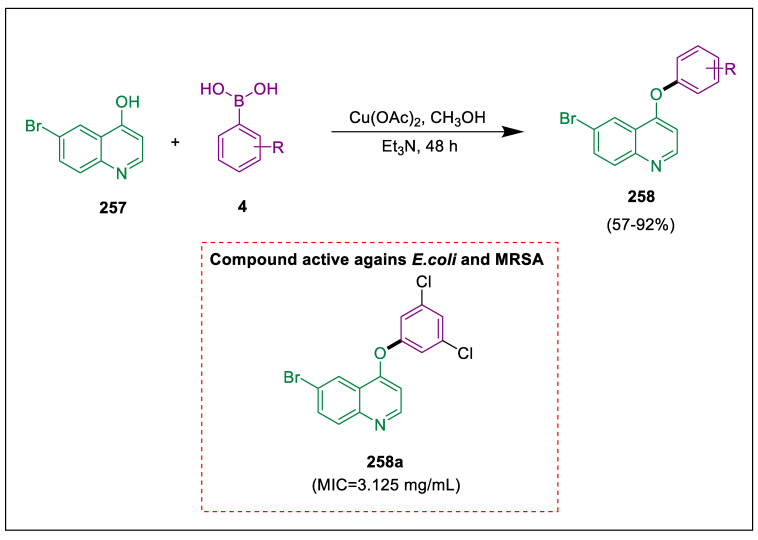
Synthesis of bromoquinolin-4-ol derivatives by using Chan-Evans-lam coupling.

ESBL *E. coli* and methicillin-resistant *Staphylococcus aureus* become resistant to *β*-lactam inhibitors and *β*-lactamase quinoline due to the accumulation of numerous antimicrobial-resistant genes [68]. Quinoline is a biologically active heterocyclic molecule that is present in pharmacological dynamics [274]. The synthesis of a novel 4-aryl-3,4-dihydro-2*H*-[1,3]oxazino[5,6-*h*]quinolin-2-one, exhibiting antibacterial action, was reported by Rbaa and colleagues. Using Chan-Evans-Lam coupling, Arshad et al. synthesized novel 6-Bromoquinolin-4-ol derivatives **258** by using 6-bromoquinolin-4-ol, which react with different boronic acids in the presence of a copper catalyst and various protic, aprotic, and mixed solvents. These derivatives show antibacterial activity against MRSA and ESBL *E. coli*. By using the Chan-Lam coupling process, between 6-bromoquinolin-4-ol **257** and with arylboronic acid **4** in the presence of Et_3_N, Cu catalyst, and molecular sieves of 4 Å in the aerobic atmosphere yields derivatives of 6-bromoquinolin-4-ol **258**. Compound **258a** possesses the largest zone of inhibition, with MIC and MBC values of 6.25 and 12.5 mg against ESBL-producing *E. coli* and 3.125 and 6.5 mg against MRSA, respectively (Scheme 49) [275].

**Scheme 50:**
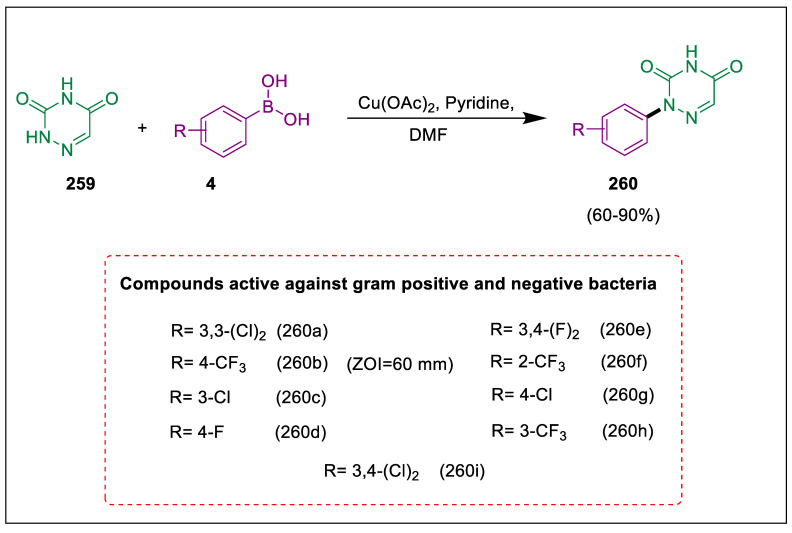
Synthesis of azauracil derivatives via Cu-catalyzed Chan-Lam cross-coupling.

Much study has been conducted on the medical uses of azauracil. The potency of 6-azauracil is enhanced by attaching a phenyl side chain at position *N*-1; thus, derivative products of azauracil consisting of *N*-aryl groups are used to form biologically active compounds [276,277,278,279]. Historically, synthesizing *N*-aryl 6-azauracil derivatives [280] involved four stages. A viable approach for synthesizing *N*-aryl 6-azauracil derivatives via Chan-Lam interaction mediated through copper. To synthesize *N*-arylated-6-azauracil derivatives, Gulipalli et al. conducted Chan-Lam coupling between azauracil and haloboronic acids in the presence of copper catalyst and pyridine. The synthesized compounds’ antimicrobial capacity was evaluated through the effective diffusion of agar. A monoarylated compound is produced when 6-azauracil **259** is treated with arylboronic acid **4** in the presence of diverse copper catalysts and base pyridine used to synthesize azauracil derivatives **260**. Compounds bearing trifluoromethyl groups, namely **260b**, **260f**, and **260h**, exhibited noteworthy antibiotic activity. The activity of substances with a chloro group attached to the phenyl rings **260a**, **260c**, **260g**, and **260i** was greater than that of products possessing fluorine **260d** and **260e** against gram-positive and gram-negative strains. In contrast, the activity of the remaining compounds was found to be moderate when compared with the drug chloramphenicol (Scheme 50) [281].

**Scheme 51:**
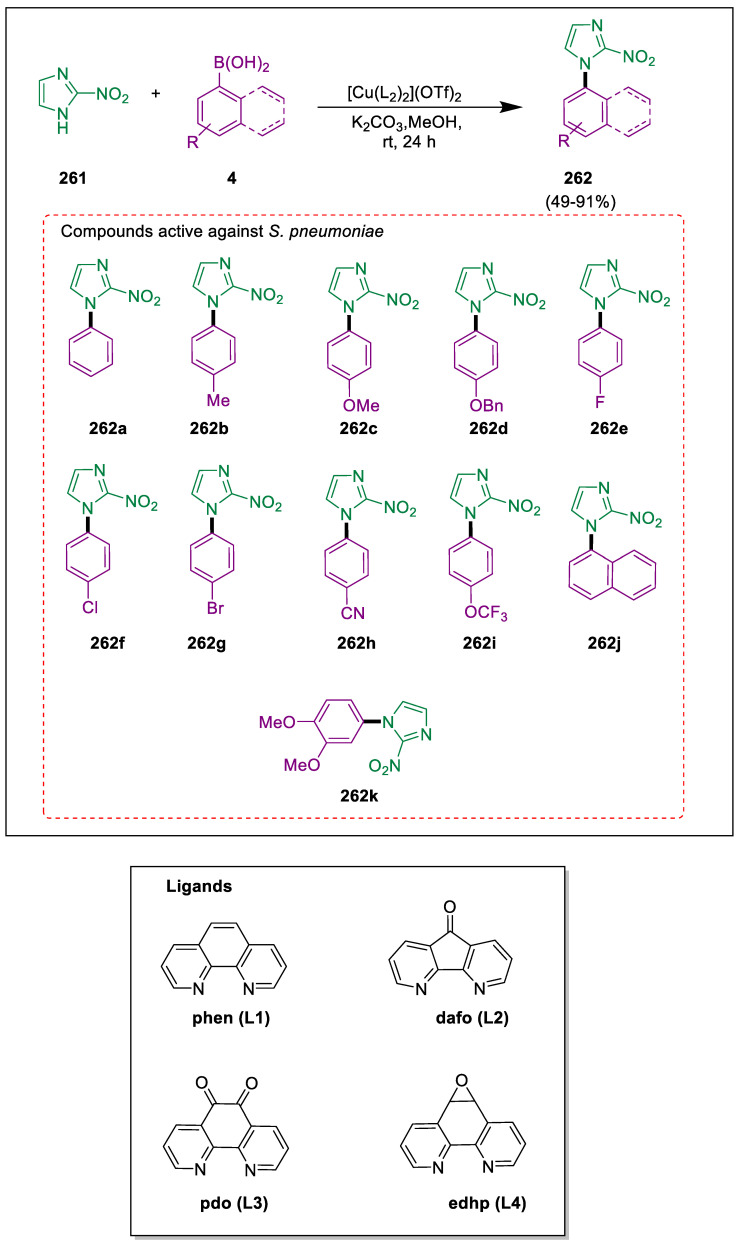
Synthesis of azomycin derivatives by using Chan-Evans-Lam cross-coupling.

Significant precursors for the synthesis of physiologically active aromatic compounds are imidazoles, nitroimidazoles, and benzimidazoles. Heteroatoms can be coupled to arenes via the Chan-Evans-Lam (CEL) coupling technique [282]. Cu(II)-catalyzed or copper(II)/cobalt(II) co-catalyzed carbon-nitrogen cross-coupling of pyrrole and boronic acid has been reported by Batey [283], Ghanbari [284], Aberi [285], and Allahresani [286]. Raju et al. used Cu-catalyzed CEL coupling to a couple of boronic acids with poorly activated imidazoles to produce targeted C-N bonds. Azomycin and many recently arylated derivatives were tested against *S. pneumoniae*; the lowest minimum inhibitory concentration (MIC) was shown by **262d**. Arylboronic acid **4** and imidazole **261** are coupled by ligands L1–L4 and a few other classical bidentate ligand systems to support Cu (II) to generate C-N coupling product **262**. Reactant demonstrates antibacterial action against several species. At 500 μM, all compounds **261**, **262a**–**k** showed an impact on the growth of *S. pneumoniae* cultures (Scheme 51) [287].

### 3.4. Knoevenagel and Michael’s Addition Reaction

**Scheme 52:**
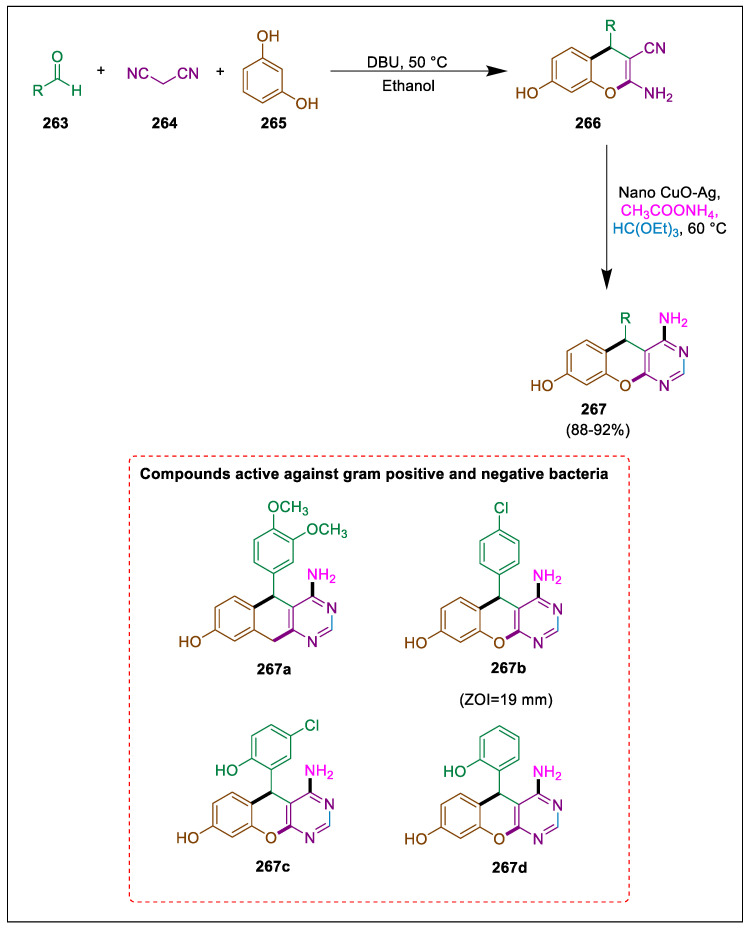
Synthesis of pyranopyrimidine derivatives by using Knoevenagel and Michael addition reactions.

A significant component of many types of naturally occurring compounds, benzopyran (also known as chromene) has biological activity [288,289,290]. Pyrimidine and its analogs have a wide range of biological and medicinal uses [291]. The synthesis of pyrano-pyrimidine derivatives in the presence of bases has been reported using several techniques; however, the yield was low. Using Knoevenagel and Michael’s addition by using nano CuO-Ag as a catalyst, Poola et al. devised a highly effective and environmentally friendly process for the synthesis of pyranopyrimidine derivatives. The antibacterial and antifungal properties of the produced target compounds **267** were examined. Using substituted aldehydes **263**, malononitrile **264**, and resorcinol **265** in the presence of ethanol and DBU, perform a one-pot, three-component domino Knoevenagel–Michael addition reaction to obtain pyran derivatives **266**. These derivatives were then cyclized in the presence of (Diethoxymethoxy)ethane, ammonium ethanoate, and a nano CuO–Ag catalyst to yield pyrano [2,3–*d*] pyrimidine derivatives **267**. Compared to the normal tetracycline, compounds **267a**–**c** showed greater activity on Gram(+ve) bacteria and **267a**–**d** on Gram(-ve) bacteria (Scheme 52) [292].

### 3.5. 1,3-Dipolar Cycloaddition Reaction

**Scheme 53:**
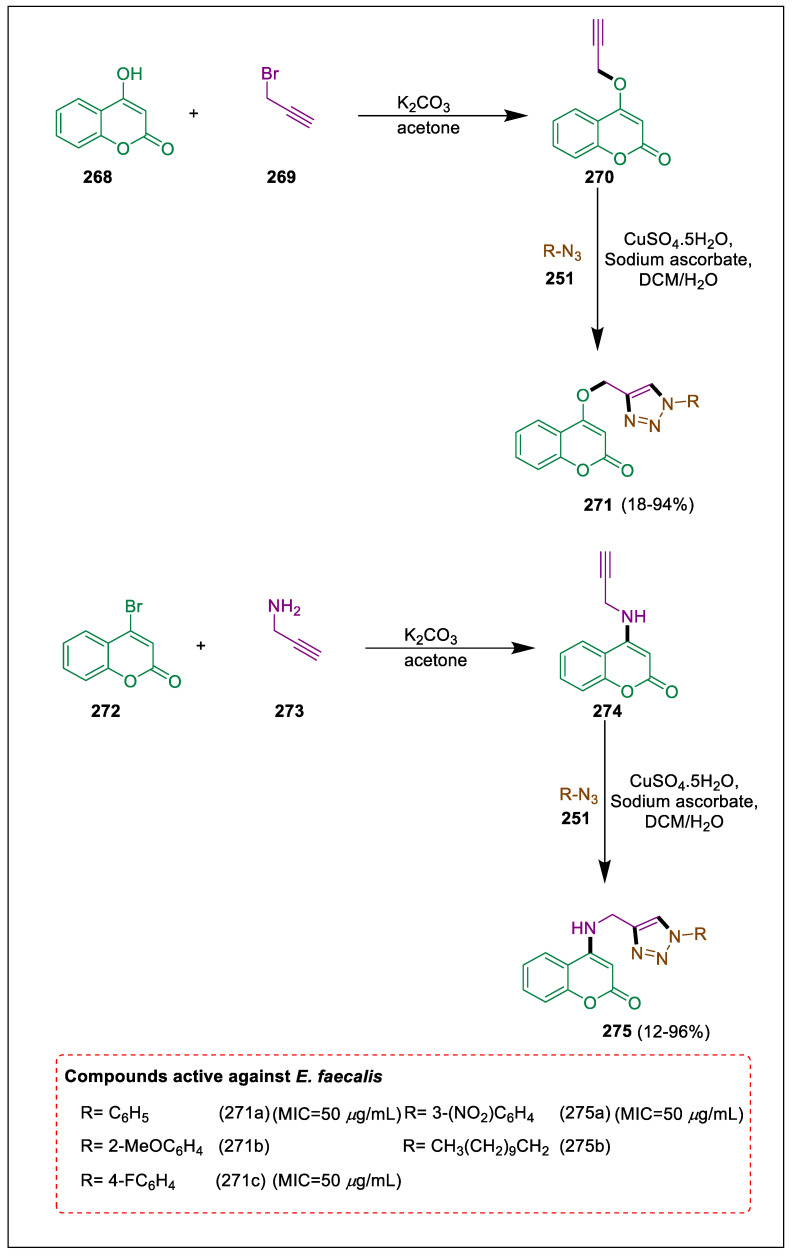
Synthesis of triazole-coumarin derivatives by using Huisgen 1,3-dipolar cycloaddition reaction.

Several extremely resilient bacterial infections have developed innovative defense mechanisms to counteract the effects of several medicines [293]. The third most often isolated nosocomial pathogen, Enterococcus, exhibits resistance to a wide range of antibiotics [294]. Because of their extensive spectrum of biological properties, coumarins and 1,2,3-triazoles are significant structures. Using a Cu(I)-catalyzed Huisgen 1,3-dipolar cycloaddition process of corresponding *O*-propargylated coumarin **271** or *N*-propargylated coumarin **275** with azides, Rojas et al. synthesized a new family of coumarin-triazole conjugates. Six out of twenty-six compounds exhibited notable antibacterial activity against *E. faecalis*. After 4-hydroxy-coumarin **268** was treated with propargyl bromide **269** using K_2_CO_3_ in anhydrous acetone, *O*-propargylated coumarin **270** was obtained, and *N*-propargylated coumarin **274** was obtained when 4-bromo-coumarin **272** underwent nucleophilic replacement with 2-propyn-1-amine **273** in DMF. The corresponding coumarins **270** and **274** undergo copper(I)-catalyzed Huisgen 1,3-dipolar cycloaddition reactions with azides **251** to provide 4-substituted 1,2,3-triazole-coumarin derivatives **271** or **275**. At MICs ranging from 12.5 to 50.0 µg/mL, compounds **271a**–**c** and **275a**,**b** showed encouraging action against *Enterococcus faecalis* (Scheme 53) [295].

**Scheme 54:**
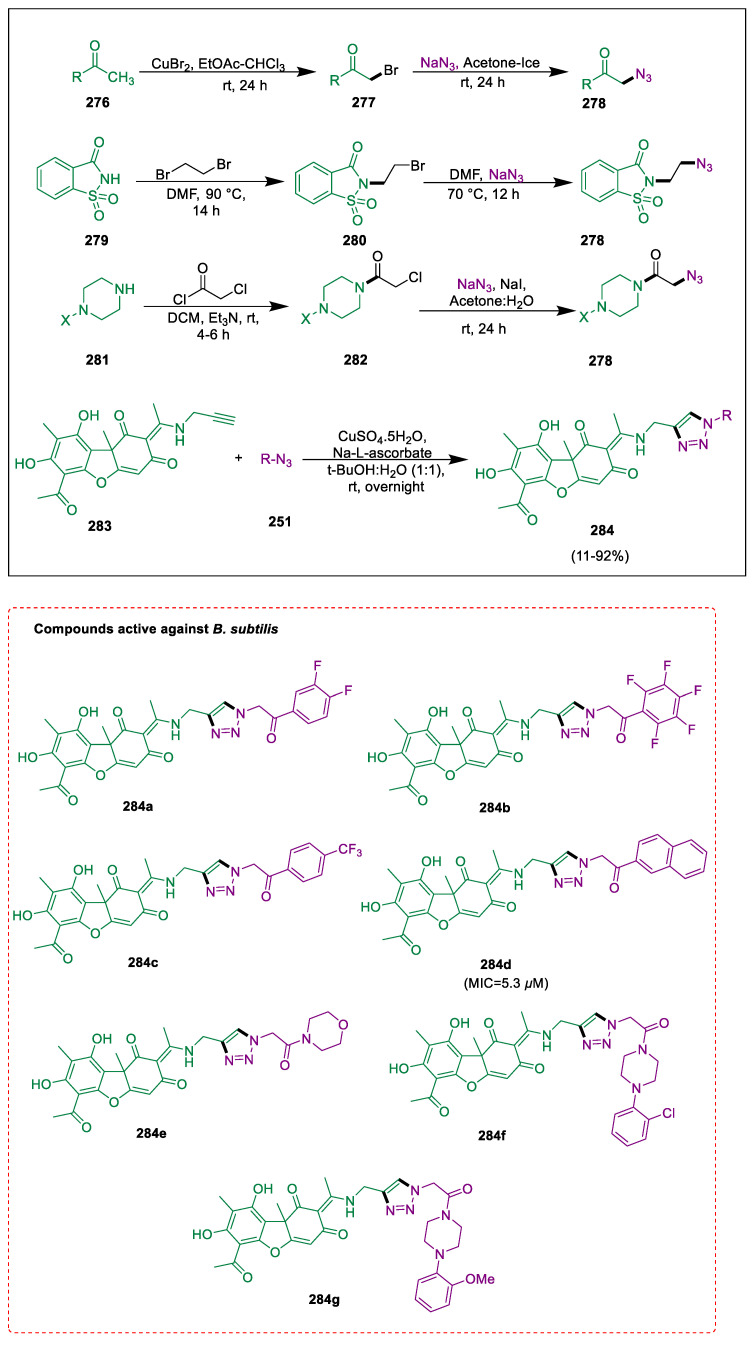
Synthesis of triazole derivatives by using 1,3-dipolar cycloaddition reaction.

Drug resistance to *Mycobacterium tuberculosis* (Mtb) evolved with the evolution of drugs. The synthesis of novel and highly effective therapeutic compounds has faced difficult challenges due to the rise of XDR and MDR tuberculosis strains [296,297] (+)-usnic acid shows biological characteristics. The usage of 1,2,3-triazoles in medical chemistry is common. Bangalore et al. synthesized novel 1,2,3-triazoles (303B_1–_B_35_) conjugated with enaminone and used them as antimycobacterial drugs. Usnic acid enaminone 303B_1_ with a terminal ethynyl moiety was obtained by condensing usnic acid with propargyl amine. Triazoles **284** were produced via a subsequent reaction with different azides A_1_–A_35_ that was catalyzed by copper. Aryl methyl ketones **276** were dissolved in either methanol or a CHCl_3_-EtOAc combination and then α-brominated to form synthetic acyl bromides **277**. Sodium azide was then added, and the mixture was agitated for a whole night and obtained azide **278**. To get one of azide **278**, stepwise reactions in the presence of dibromoethane in DMF and NaN_3_ were carried out with saccharin sodium salt **279**. To produce other azides **278**, *N*-substituted piperazines **281** reacted with chloroacetyl chloride in the presence of triethyl amine using CH_2_Cl_2_ to form intermediate **282**, then this mixture was dissolved in acetone-water and catalytic NaI and sodium azide were added and was agitated overnight. By interacting **283** with the produced azides **251** in the *t*-BuOH-H_2_O system in the presence of a copper catalyst and sodium-*L*-ascorbate, compounds **284** were synthesized using the 1,3-dipolar cycloaddition method. **284f** and **284g**, two compounds, exhibited a moderate level of antibacterial activity. Compound **284e** demonstrated a 13 mm diametrical inhibitory zone on *B. subtilis* in addition to its antitubercular activity, and compound **284c** demonstrated the highest antibacterial potency with moderate antitubercular activity. Additionally, it was discovered that active antitubercular compounds **284a**, **284b**, and **284d** had an antibacterial impact on *B. subtilis* (Scheme 54) [252].

**Scheme 55:**
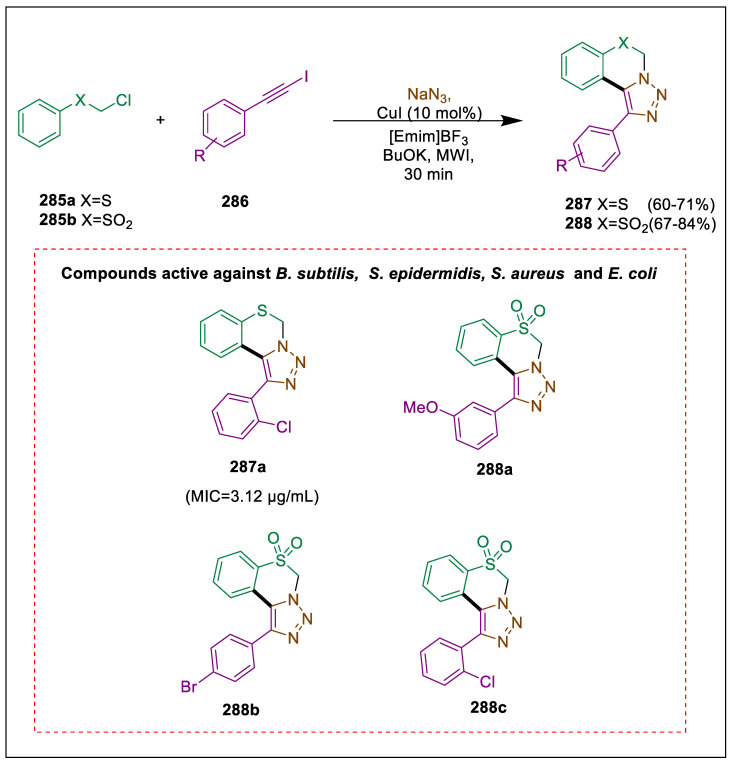
Synthesis of triazole derivatives by using Cu-catalyzed 1,3-dipolar cycloaddition (CuAAC).

1,3-Thiazine and benzothiazine heterocycles are noteworthy because of their diverse biological properties [298,299,300]. Fused triazoles and their hybrids have pharmacological characteristics [301]. We synthesized several completely decorated 1,2,3-triazoles employing a cheap Cu catalyst and sequential 1,3-dipolar cycloaddition followed by intramolecular arylation under microwave irradiation. Sucharitha et al. performed a one-pot reaction of three components that included copper-catalyzed arylation after Cu-catalyzed 1,3-dipolar cycloaddition (CuAAC) of the resultant triazole under microwave circumstances in an atmosphere of an ionizing liquid BF_4_. The novel manufactured compounds were tested against bacterial strains. Fused triazole and its derivatives **287** or **288** are produced by reacting either chloromethyl phenyl sulfur **285a** or chloromethyl phenyl sulfone **285b** with 1-(iodoethynyl)-4-substituted benzene **286** in the presence of sodium azide, CuI with 2 equivalents of *t*-BuOK in [Emim]BF_4_ under MWI at 200 W. Compounds **287a**, **288a**–**c** exhibited considerable bacterial inhibitory action against all tested positive strains and *E. coli* with MICs ranging from 3.12 to 12.5 µg/mL, and compound **288c** also demonstrated much more inhibitory activity than conventional ciprofloxacin (Scheme 55) [302].

### 3.6. Copper-Catalyzed Azide-Alkyne Cycloaddition

**Scheme 56:**
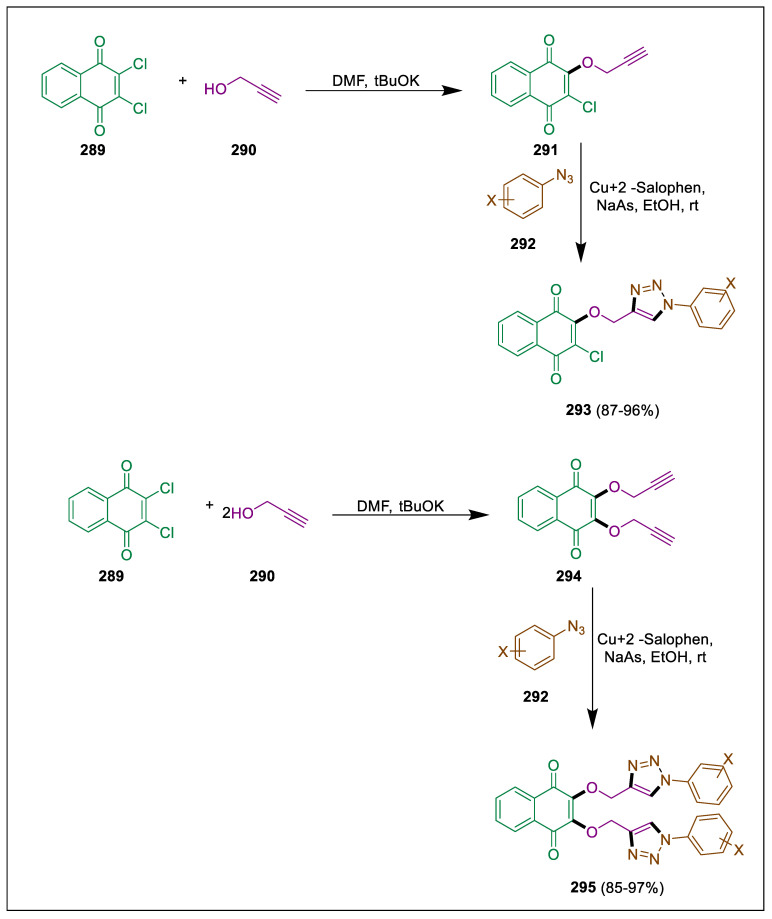

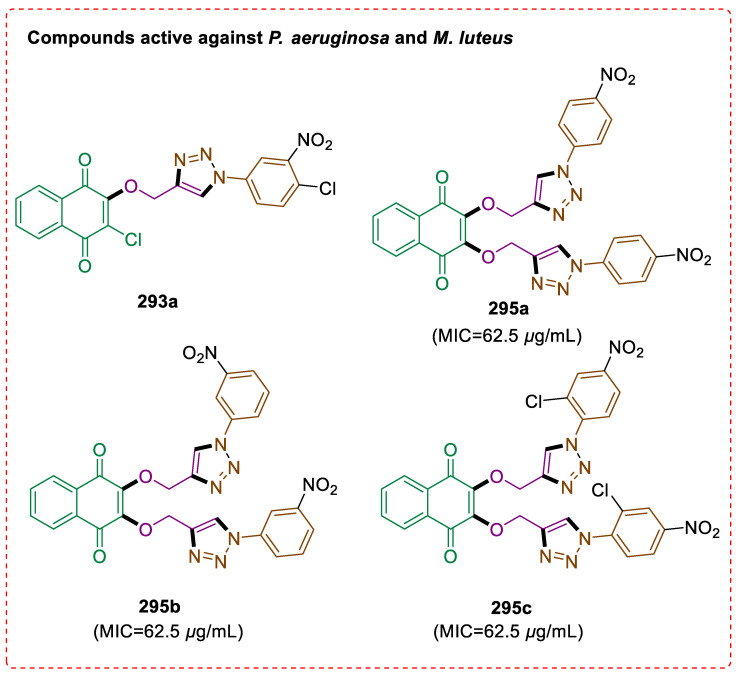
Synthesis of naphthoquinone derivatives by using copper-catalyzed azide-alkyne cycloaddition.

In organic and pharmaceutical chemistry, transition metal-catalyzed coupling reactions are crucial. Triazoles have a huge variety of applications in synthetic, medicinal, and material chemistry [303,304]. Cu-catalyzed azide-alkyne cycloaddition (CuAAC) reactions are considered the most suitable method for the preparation of 1,4-disubstituted triazoles. Naphthoquinone, alongside its derivatives, shows broad biological activities. Abbaspour et al. performed the reaction of naphthoquinone with the aromatic azides in the presence of a lower Cu catalyst loading, which provided phenyl naphthoquinone, individually and screened for antibacterial activity. The reaction of dichloronaphthalene-1,4-dione **289** with propynol **290** in the presence of *t*-BuOK and DMF afforded 2-chloro-3-(prop-2-yn-1-yloxy)-1,4-naphthoquinone **291** when two equiv. of propynol **290** were employed, 2,3-bis(prop-2-yn-1-yloxy)-1,4-naphthoquinone **294** was produced. Subsequently, compound **291** and compound **294** react with various aromatic azides **292** in ethanol (EtOH) with salophen copper (II) complex and sodium ascorbate to produce the intended aryl substituted 1,4-naphthoquinone **293** and **295**. Compounds **295a**–**c** showed advanced anti-bacterial activities as compared to the other derivatives (MIC, MBC = 62.5 μg/mL). The anti-bacterial activity of **293a** was approximate to that of penicillin with a strong inhibition (MIC = 500 μg/mL); however, other compounds show more enhanced activity than penicillin (Scheme 56) [1].

**Scheme 57:**
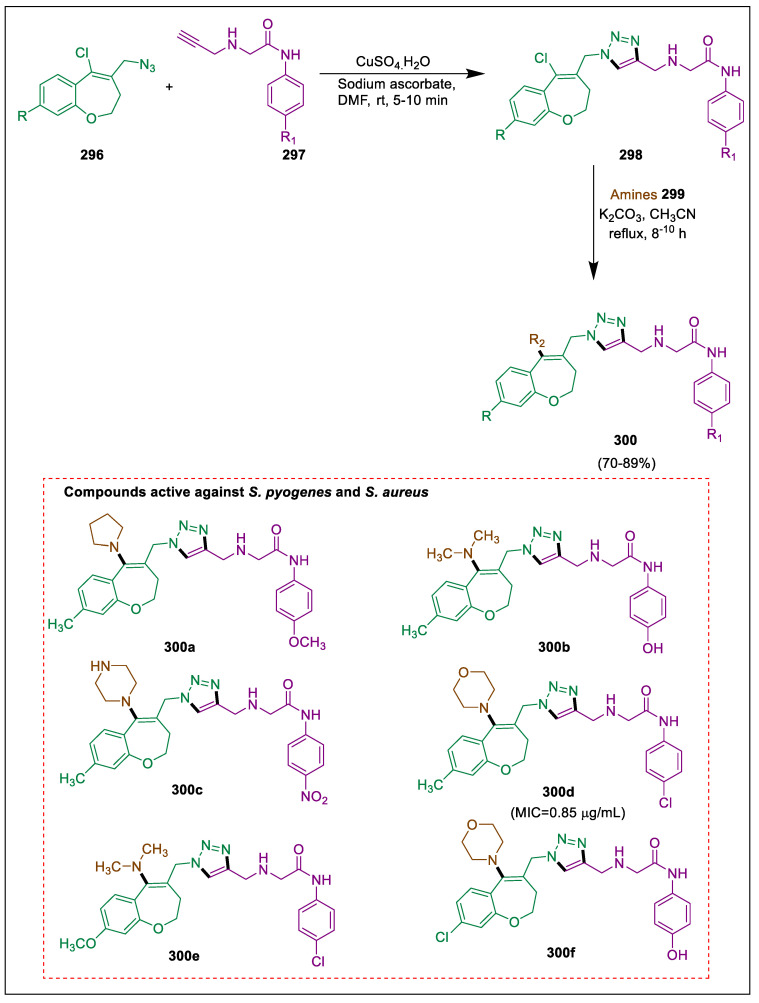
Synthesis of dihydrobenzoxepin connected to triazole derivatives by using CuAA.

Presently accessible antimicrobial drugs come with several disadvantages. By using click chemistry, heterocyclic compounds with triazole moiety can be synthesized. The click reaction known as the 1,3-dipolar cycloaddition of alkynes and azides, which is catalyzed by Cu, provides a productive approach to synthesizing 1,2,3-triazoles [305]. These compounds are utilized in drug production and exhibit a wide range of biological activity. An essential role played by benzoxazepines in medicinal chemistry [306]. A unique series of dihydrobenzoxepin connected to triazole scaffolds (**300a**–**f**) was synthesized by Gandham et al. along with their pharmacological activity against various strains of fungi, bacteria Gram-(+ve) and Gram-(–ve). According to biological investigations, compounds **300a-f** have zones of inhibition that range from 33–30 mm to 32–28 mm, making them good antibacterial and antifungal agents. *N*-phenyl-2-(prop-2-yn-1-ylamino)acetamide **296** and liquid terminal alkyne **297** undergo CuAAC reaction in the presence of pre-catalyst CuSO_4_⋅5H_2_O and sodium-ascorbate in DMF to yield **298**, which then reacts with corresponding cyclic, alicyclic amines **299** to yield triazole derivatives **300**. Triazoles **300a**, **300b**, **300e**, and **300f** showed moderate to better antibacterial activity against *S. pyogenes* and *S. aureus*, whereas compounds **300c**, **300d**, **300e**, and **300f** showed greater activity with MIC values of 1.2, 0.85, 2.5, and 2.9 μg/mL (Scheme 57) [307].

### 3.7. Oxidative Cross-Dehydrogenative Coupling

**Scheme 58:**
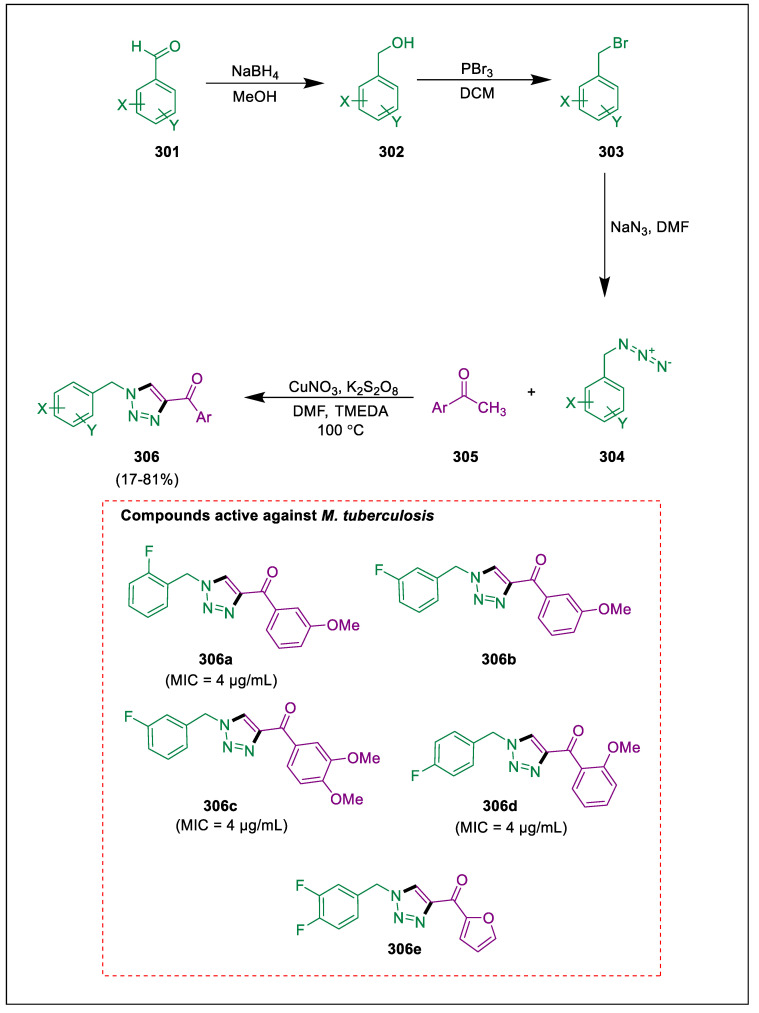
Synthesis of arylmethanone derivatives by using oxidative cross-dehydrogenative coupling.

The overuse of antibiotics is contributing to antibiotic resistance, which is a serious problem that is endangering public health [308]. To solve these problems, it may be essential to find novel antibiotics made from heterocyclic compounds, particularly those that include nitrogen that are well-known for their pharmacological characteristics. The use of triazoles in pharmaceuticals has increased. Turkmen et al. employed a one-pot Cu-Catalyzed Oxidative Cross-Dehydrogenative Coupling/Oxidative Cycloaddition technique to manufacture a novel series of triazoyl arylmethanone derivatives. The most effective compounds with lower minimum inhibitory concentrations (**306a**, **306c**, and **306d**) demonstrated high antimycobacterial activity of the aryl methanone derivatives against *Mycobacterium tuberculosis*. The synthesis of organic azides **304** involves three phases, which undergo a one-pot copper-catalyzed [3 + 2] cycloaddition reaction using ketone derivatives **305** in the presence of a copper catalyst to synthesize 4-(1,2,3-triazoyl)arylmethanone derivatives **306**. Comparing the compounds **306a**, **306c**, and **306d** (MIC = 4 µg/mL [12.8, 11.7, and 12.8 µM, respectively]) to the standard medication, they showed good inhibition; in contrast, compounds **306b** and **306e** (MIC = 8 µg/mL [25.7 and 27.7 µM, respectively]) demonstrated moderate inhibition (Scheme 58) [309].

### 3.8. Cyclization of Thioureas

**Scheme 59:**
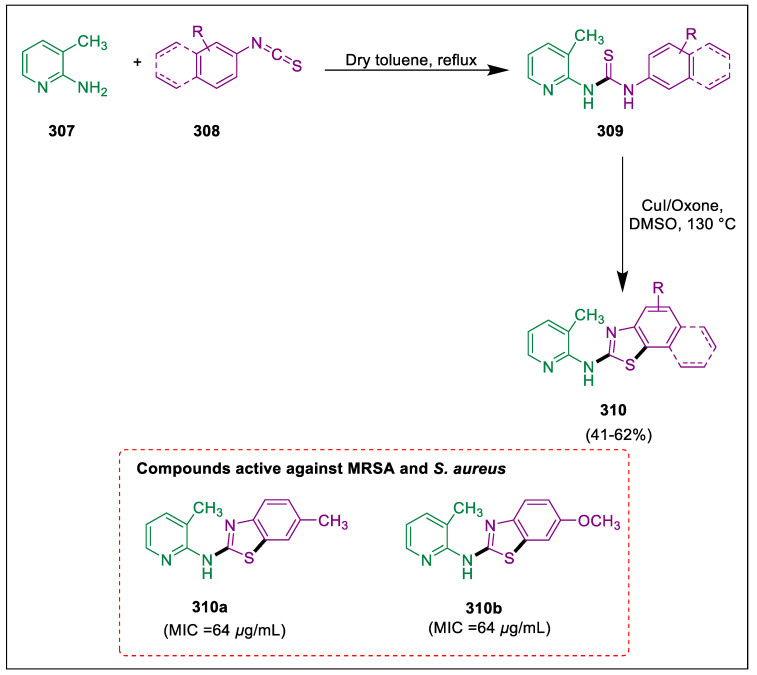
Synthesis of aminobenzothiazole derivatives by using CuI/oxone as a catalyst for cyclization of thioureas.

Novel antibacterial drugs are synthesized using derivatives of benzothiazoles. The condensation reactions of 2-aminothiophenol with derivatives of carboxylic acids are part of the conventional pathway for BTA scaffolding [310]. Although palladium [311], copper(II) [312], ruthenium [313], and iron [312] catalysts are used for the synthesis of 2-aminobenzothiazoles by intramolecular cyclization of thioureas. Using copper iodide/oxone as a catalyst, Doğan et al. devised a practical, quick, and effective approach for the ring closure reaction of thioureas to generate 2-aminobenzothiazole derivatives. The antimicrobial properties of compounds were evaluated using a range of microbial strains. The thiourea-based necessary cyclization precursors **309** were synthesized by reacting 3-methylpyridin-2-amine **307** with an equivalent quantity of different isothiocyanate derivatives **308** in toluene at 80 °C. CuI/oxone was then used to catalyze the cyclization of these precursors **309** to produce 2-aminobenzothiazole derivatives **310**. Apart from compounds **310a** and **310b**, all compounds exhibited antibacterial activity against all tested bacteria, with MIC values ranging from 32 to 1024 μg/mL. While **310b** had a MIC value of 32 μg/mL against three gram-positive bacteria, making it more effective against bacteria. Moreover, **310a**,**b** attracts interest with a MIC value of 64 μg/mL against MRSA and *S. aureus* (Scheme 59) [314].

### 3.9. Copper-Catalyzed 1,2-Addition

**Scheme 60:**
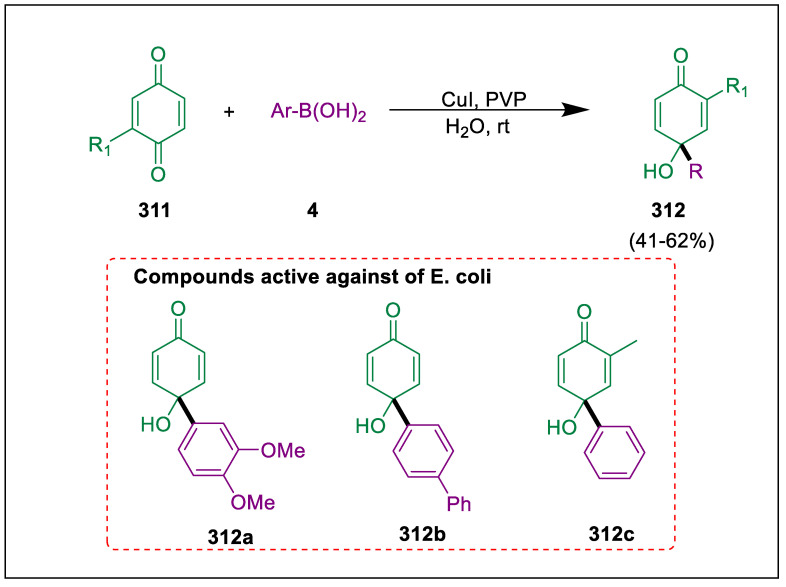
Synthesis of *p*-quinol derivatives by using copper-catalyzed 1,2-addition.

Many bioactive natural products have skeletons of *p*-quinol [315,316,317,318], and their glycosides have been shown to have analgesic properties [319]. An effective technique for separating *p*-quinol derivatives containing aryl groups and testing them against pathogenic strains of *E. coli*. And overcome the limitations where *p*-quinols are obtained on the basis of dearomatization [320]. *p*-quinols were synthesized in an aqueous medium by Koszelewski et al. The antibacterial properties of the *p*-quinols were evaluated against *E. coli*, and the additives played an important role in the copper-catalyzed addition of aryl and heteroaryl boronic acids to produce benzoquinones. To synthesize *p*-quinols **312**, a model addition reaction involving 1 mmol of arylboronic acid **4** and 1 mmol of benzoquinone **311** was carried out in distilled H_2_O at 20 °C with atm using a CuI-PVP catalytic system. Based on the MIC and MBC values, synthesized compounds **312a**–**c** showed high antimicrobial activity (Scheme 60) [321].

## 4. Nickel-Catalyzed Synthesis

The focus of academia and industry has recently shifted to efficient chemistry, which uses inexpensive and environmentally friendly reagents in transformations designed with high atom economy, starting from feedstocks sourced from biofuels and going beyond small molecules in strategies to produce supramolecular combinations [322,323] and designed macromolecular materials [324]. Numerous procedures have been studied for the synthesis of relatively big organic compounds and polymers, but often the linking of prefabricated components together is more expeditious [325,326,327]. Early transition metals such as Ni were discovered to be helpful reagents and catalysts early in the development of homocoupling, the combining of chemical fragments, and cross-coupling.

For cross-coupling processes, inexpensive and readily available nickel (Ni) catalysts are a good substitute for precious metal catalysts such as Pd. Furthermore, Ni demonstrates distinct reactivity, resulting in various pathways. In 1972, a nickel catalyst was used to perform the cross-coupling of organic halides with Grignard reagents; this reaction is now referred to as the Kumada-Tamao-Corriu process. Ni catalysts perform better in some reactions if the substrates are heteroaryl [328]. Therefore, one of the most effective synthetic techniques is Ni-catalyzed cross-coupling, which is widely utilized to generate complex compounds, active medicinal components, and precursors for materials chemistry [329,330].

### 4.1. Kumada Cross-Coupling

**Scheme 61:**
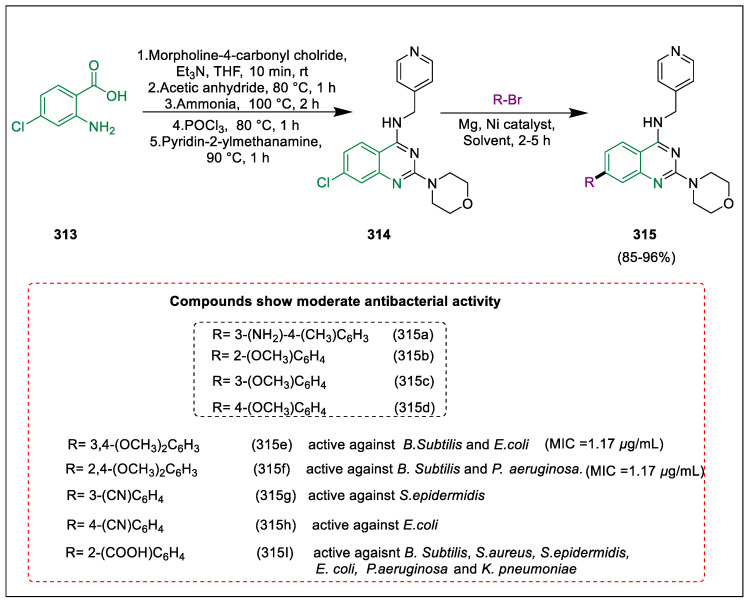
Ni-catalyzed Kumada cross-coupling to synthesize quinazoline amine and its derivatives.

The incidence of antimicrobial resistance (AMR) persistently increases, creating opportunities for developing novel antibacterial agents [272,331,332]. Quinazoline derivatives are utilized in many medicinal contexts [333,334,335,336,337,338,339,340,341,342,343,344,345]. The synthetic routes for the quinazoline derivatives, which yield the desired compounds in five steps requiring column extraction for each step’s product, have been previously documented by a group [346]. Considering this, the two-step synthesis of quinazoline derivatives is described in this article. In their investigation, Ankireddy et al. utilized a nickel-catalyzed Kumada cross-coupling process to produce substituted-2-morpholino-*N*-(pyridin-2-ylmethyl)quinazoline-4-amine derivatives **315**. Antibacterial activity tests were conducted on all the target compounds against gram-positive and gram-negative bacteria. The findings showed compounds **315e**–**i** demonstrated significant antibacterial efficacy. 4-Chloroanthranilic acid **313** treated with morpholine-4-carbonyl chloride in tetrahydrofuran stirred at room temperature for 1 h. Acetic anhydride was added and stirred at 80 °C for 1 h, add NH_3_ solution was stirred at 100 °C for 2 h, then phosphoryl trichloride was stirred at 80 °C for 1 h, then pyridine-2-yl-methanamine was added and stirred at 90 °C for 1 h to obtain compound **314**, which undergoes Kumada cross-coupling reaction with various aryl bromides by using five Ni catalysts to obtain quinazoline derivatives **315**. **315e**–**i** exhibited comparable antibacterial activity against susceptible bacteria but were noticeably more effective against resistant ones, whereas **315a**–**d**, which has an electronegative atom attached to the aromatic group, demonstrates moderate antibacterial effectiveness (Scheme 61) [346].

**Scheme 62:**
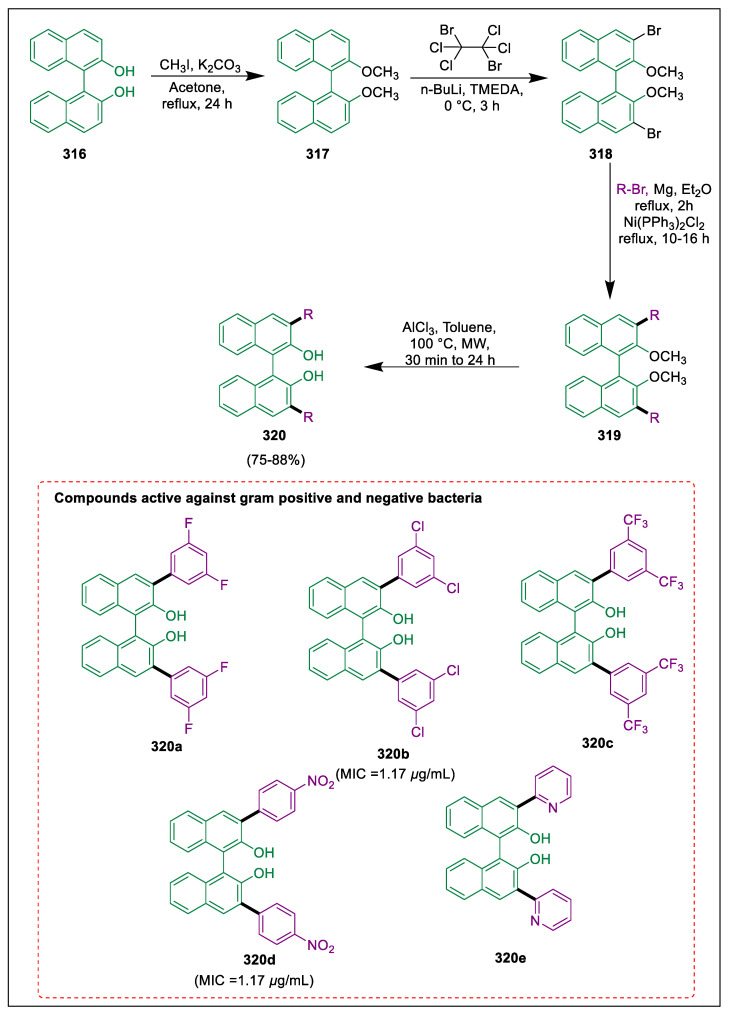
Synthesis of BINOL derivatives by using Suzuki and Kumada cross-coupling.

The medical management of diseases induced by numerous gram-positive and gram-negative bacteria has proven difficult due to their resistance to microorganisms [346,347]. Given these factors, there is an ongoing demand for novel chemical entities that can effectively tackle the problem of multidrug resistance. A variety of chiral macrocycles and antibacterial substances were synthesized through the utilization of BINOL derivatives. In their study, Ankireddy et al. utilized the Kumada and SMC reactions to produce a novel series of disubstituted chiral (*S*)-BINOL derivatives **320**. Additionally, the authors assessed the antibacterial activity of these compounds against gram-positive and gram-negative bacteria. Compounds **320a**–**d** have been located to possess the most potent antibacterial activity. The Kumada coupling yield and reaction time are more favorable than the Suzuki coupling [348]. After BINOL **316** was treated with potassium carbonate and MeI, (*S*)-2,2′-dimethoxy-1,1-binaphthalene **317** was produced. This compound was then treated with *n*-butyllithium, *N*, *N*, *N*′, *N*′-tetramethylethylenediamine, and dibromo tetrachloroethane, resulting in the formation of (*S*)-dibromodimethoxy binaphthalene **318** [349], and it undergoes the Kumada coupling reaction [350], giving the corresponding products **319** [351]. Then subjected to demethylation under the action of BBr_3_ at room temperature [352] or AlCl_3_ upon heating and MW irradiation synthesized compound **320**. All the synthesized compounds were subjected to in vitro antibacterial efficacy testing [353] and were found to be exceedingly potent. The compounds that exhibited the highest level of antibacterial capability against every type of bacteria were identified as **320a**–**e**, with MIC values ranging from 1.17 to 4.68 μg/mL (Scheme 62) [348].

### 4.2. Buchwald-Hartwig Reaction

**Scheme 63:**
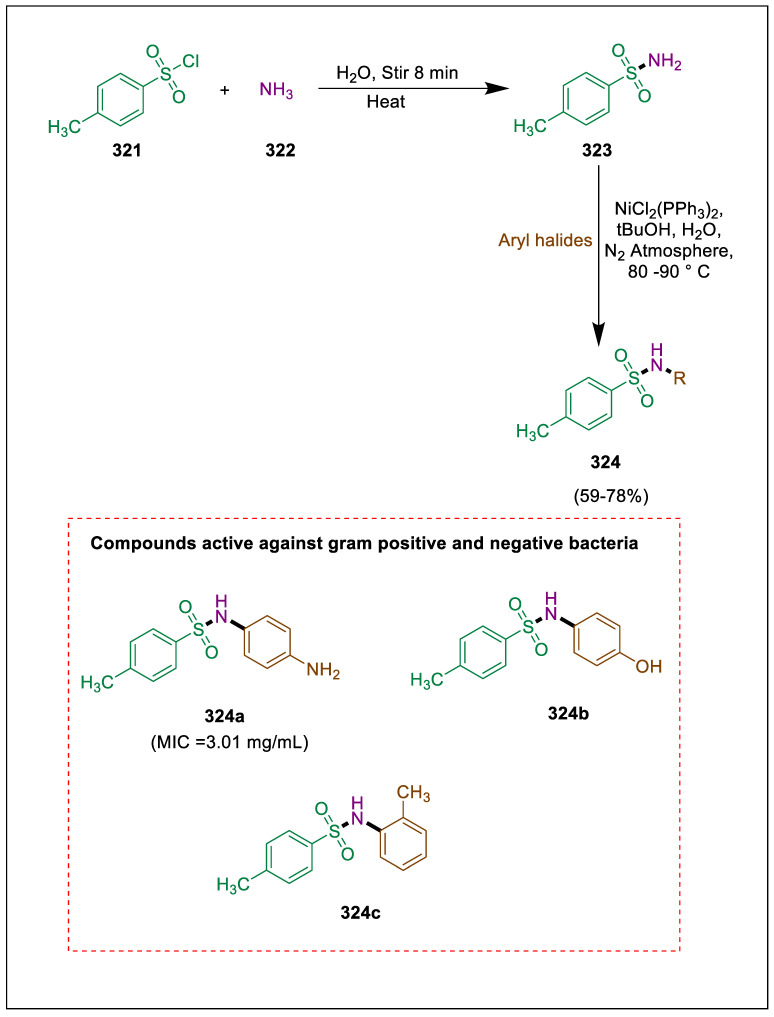
Synthesis of sulphonamide derivatives by using Buchwald-Hartwig reaction.

The synthesis of sulphonamides is an interesting topic in medicinal chemistry [354]. Sulphonamides are commonly employed in medicinal chemistry due to their high biological activity. Nassir et al. described a variety of bis-imidazole and bis-benzimidazole sulphonamides that were proven effective against bacteria. The synthesis of the C-N moiety in compounds by tandem reactions catalyzed by nickel is not well documented. Izuchukwu et al. effectively synthesized methylbenzensulphonamide and its Ni-catalyzed transformation to *N*-aryl substituted *p*-toluene sulphonamides **324** using the Buchwald-Hartwig tandem amidation technique. These compounds were tested for antibacterial and antifungal activity. Some of the substances showed greater efficacy when compared to the standard drugs (ketoconazole and ciprofloxacin). After 8 min of stirring 4-methylbenzene sulphonyl chloride **321** with ammonium hydroxide **322** in water and 5 min of heating, 4-methylbenzenesulphonamide **323** was obtained. This reacted with the appropriate aryl halides in the presence of Ni catalyst, and a solvent of *t*-butanol and water was pre-activated. After 5 min of stirring and heating at 80 °C for 90 min, K_2_CO_3_ was added along with additional *t*-butanol, and after refluxing with stirring for 1 h at 110 °C, corresponding *N*-aryl substituted methyl benzenesulfonamide **324** was obtained. The antibacterial activity of these compounds was compared to the standard medication ciprofloxacin. The MIC of most of the sulphonamides was found to be higher than the standard. **324a**–**c** are the most effective antibacterial compounds (Scheme 63) [355].

### 4.3. Photocatalytic Cross-Coupling

**Scheme 64:**
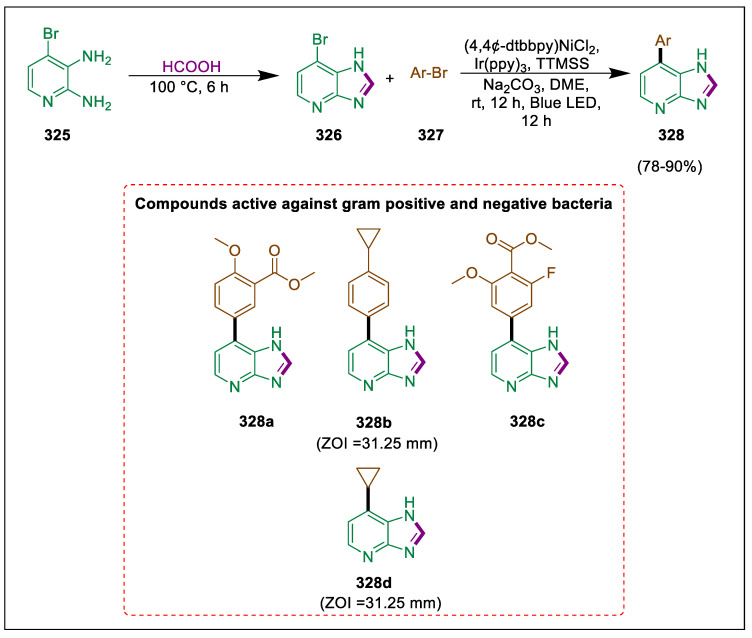
Synthesis of imidazo pyridine derivatives by using photocatalytic cross-coupling.

Because of their possible pharmacological properties, medicinal chemistry researchers are paying more attention to heterocyclic compounds that include imidazole pyridines [356,357,358]. A multitude of synthetic techniques have been established for the synthesis of imidazopyridines [359], which find application in pharmaceuticals. It has been demonstrated that imidazo [4,5-*b*] pyridine, one of the isomers, and its derivatives show a variety of biological functions. Under mild conditions, Jebamani et al. reported an effective approach for photocatalytically producing novel 7-aryl-1*H*-imidazo[4,5-*b*]pyridines **350** using the C-C cross-coupling process. Some compounds showed strong antimicrobial activity against both antibacterial and antifungal strains when compounds **370** were tested. After refluxing 4-bromopyridine-2,3-diamine **347** with triethyl-*ortho*-formate, the scaffold 7-bromo-1*H*-imidazo[4,5-*b*]pyridine **348** was produced. This scaffold then underwent a photocatalytic cross-coupling approach with the use of dual catalytic and single electron-transfer mechanisms with heteroaryl bromides **349** to produce 7-aryl-1*H*-imidazo[4,5-*b*] pyridines **350**. Compounds **350a** and **350c** were identified as effective bactericides towards *S. faecalis* and *B. subtilis*, respectively, and consequently moderate responses towards *K. pneumonia*, *E. coli*, and *P. aeruginosa*. Compounds **350b** and **350d** demonstrated remarkable anti-bacterial responses against gram-positive and gram-negative bacteria (Scheme 64) [360].

## 5. Conclusions

Transition metal-catalyzed reactions are employed in synthetic and pharmaceutical sectors. These reactions have enabled the synthesis of complicated pharmaceutical scaffolds, while catalysis with transition metals such as Pd, Ni, and Cu has brought efficiency to the synthesis of approved drug candidates that are now being developed in labs all over the world [361,362,363,364,365]. The last five years’ research on the synthesis of antibacterial molecules via Pd, Cu, and Ni-catalyzed reactions is provided in a comprehensible manner, keeping in mind that these synthetic approaches may inspire pharmaceutical researchers. Since it is quite challenging to design a drug without knowing its chemical structures, which in return provide a gateway of compatibility with the target protein inside the human body. Thus, it is essential to have knowledge about some bioactive molecules that may prove to be the next most effective drug. Scheme numbers with their methods and against which these compounds show activity summarized in Table 1.

## Figures and Tables

**Table 1:** Key synthetic methods and antibacterial compounds.

Scheme Number	Synthetic Method	Compounds	Activity Against
Pd-Catalyzed Synthesis			
Scheme 1	Suzuki-Miyaura cross-coupling produces moderate to acceptable yields	**5**, **6**, **3**	*E. coli*
Scheme 2	Suzuki-Miyaura coupling	**9**, **11**	Gram-positive bacteria
Scheme 3	Masuda borylation-Suzuki coupling	**14**, **17**	MRSA, gram-positive bacteria
Scheme 4	Suzuki cross-coupling and cyclization	**24b**	*S. aureus*
Scheme 5	Microwave-assisted Suzuki coupling	**30a**, **30b**, **30c**, **30d**	Various bacterial strains
Scheme 6	Pd-catalyzed Suzuki coupling	**34a**, **34b**, **34c**	Various bacterial strains
Scheme 7	Metal-catalyzed Suzuki coupling	**40**, **40a**, **40b**	NDM-1-positive A. baumannii, *K. pneumoniae*
Scheme 8	Pd-catalyzed Suzuki coupling	**47**	Gram-positive and negative bacteria
Scheme 9	Suzuki-Miyaura reaction by using 100 °C temperature	**51**, **52**, **53**, **54**	Various bacterial strains
Scheme 10	Cyclization and Suzuki-Miyaura process	**59**, **61**	*P. aeruginosa*, *S. aureus*
Scheme 11	Suzuki-Miyaura reaction in inert atmosphere	**65**, **65a**, **65b**	ESBL-producing *E. coli*
Scheme 12	Suzuki-Miyuara coupling	**68**, **69**, **69a**, **69b**	XDR pathogens
Scheme 13	Pd-catalyzed Suzuki coupling reaction	**71a**, **71e**, **71g**	*P. aeruginosa*, *E. coli*
Scheme 14	Pd-catalyzed Suzuki cross-coupling	**79a**, **79b**, **79c**, **79d**, **79e**	*E. coli*, *S. agalactiae*
Scheme 15	Pd-catalyzed Suzuki cross-coupling	**84**, **86**	*M. luteus*
Scheme 16	Suzuki-Miyaura coupling	**90**	*P. aeruginosa*
Scheme 17	Suzuki cross-coupling	**94**, **95**	*E. coli*
Scheme 18	Pd-catalyzed Suzuki cross-coupling	**100**, **102**, **104**, **106**	Gram-positive and negative bacteria
Scheme 19	Suzuki cross-coupling using Pd (PPh_3_)_4_ catalyst	**113**, **114**	*S. Typhimurium*, *S. aureus*
Scheme 20	Suzuki-Miyaura coupling	**116**, **117**, **117a**, **117b**, **117c**, **117d**	MRSA, *E. coli*
Scheme 21	Suzuki-Miyaura cross-coupling	**120a**, **121a**, **122**	Gram (+) bacteria
Scheme 22	Suzuki-Miyaura cross-coupling	**126**, **126c**, **126f**, **126e**	*S. typhaes*, *Bacillus subtilis*, *Pseudomonas aeruginosa*, and *E. coli*
Scheme 23	Suzuki cross-coupling	**134**	*S. aureus*, *E. coli*
Scheme 24	Suzuki-Miyaura coupling	**137**, **138**	ESBL *E. coli* ST405, MRSA
Scheme 25	Suzuki cross-coupling	**141**	*B. subtilis*
Scheme 26	Suzuki-Miyaura cross-coupling	**144**	*B. subtilis*, *S. aureus*
Scheme 27	Pd-catalyzed Suzuki cross-coupling	**151**	Gram-positive and negative bacteria
Scheme 28	SMC process	**155**	Gram-positive and negative pathogens
Scheme 29	Suzuki cross-coupling with Pd(dppf)Cl_2_	**161**	*P. aeruginosa*, *E. coli*
Scheme 30	Suzuki cross-coupling in the presence of K_3_PO_4_, Pd catalyst, and dioxane	**164**, **165**	*E. coli*, *S. aureus*
Scheme 31	Pd-catalyzed Suzuki cross-coupling	**173**	MRSA, *S. aureus*, MSSA
Scheme 32	Sonogashira cross-coupling (copper-free)	**177a**–**g**	*Micrococcus luteus*, *Pseudomonas aeruginosa*
Scheme 33	Multi-component Sonogashira cross-coupling (copper-free)	**181a**–**g**	*M. luteus*, *P. aeruginosa*
Scheme 34	Sonogashira cross-coupling (copper-free)	**185a**–**b**	*B. subtilis*, *P. aeruginosa*
Scheme 35	Sonogashira cross-coupling	**191a**, **189a**	*S. aureus*, *P. aeruginosa*
Scheme 36	Sonogashira cross-coupling followed by TBAF deprotection	**199a**–**c**	MRSA, *P. aeruginosa*
Scheme 37	PPE derivatives using Sonogashira polymerization	**202**	*E. coli*
Scheme 38	Dione derivatives using Sonogashira cross-coupling	**207a**–**d**	*M. luteus*, *P. aeruginosa*
Scheme 39	Pd-catalyzed Stille coupling	**212**	*E. faecalis* ATCC 29,212
Scheme 40	Stille coupling and Sonogashira coupling	**215**, **219**	*S. aureus* ATCC 25923, *P. aeruginosa* ATCC 27853
Scheme 41	Buchwald-Hartwig coupling	**224**	*S. aureus*, *B. subtilis*, *E. coli*
Scheme 42	Buchwald-Hartwig reaction	**229**	Various gram-positive bacteria
Scheme 43	Buchwald-Hartwig reaction	**232**	*B. cereus*, *S. aureus*
Scheme 44	Pd-catalyzed Heck reaction	**235**	*M. luteus*, *P. aeruginosa*
Scheme 45	Reductive Heck reaction	**241**	*M. luteus*, *M. abscessus*, and *S. murinus*
Scheme 46	Pd-catalyzed 1,4-addition to dienes	**245E1**–**E5**	*Xanthomonas oryzae pv. Oryzae (Xoo)*
Cu-Catalyzed Synthesis			
Scheme 47	Cu (II) salen complex-catalyzed click reaction	**252a**, **252b**, **252c**	*B. subtilis*, *M. luteus*
Scheme 48	Cu-catalyzed Ullmann cross-coupling	**256a**, **256b**, **256c**	*S. aureus* (resistant to methicillin)
Scheme 49	Chan-Evans-Lam cross-coupling	**258a**	MRSA, ESBL *E. coli*
Scheme 50	Chan-Lam cross-coupling	**260**	Gram-positive and gram-negative strains
Scheme 51	Cu-catalyzed Chan-Evans-Lam cross-coupling	**262**	*S. pneumoniae*
Scheme 52	CuO–Ag-catalyzed Knoevenagel and Michael’s addition	**267**	Gram-positive and gram-negative bacteria
Scheme 53	Cu(I)-catalyzed Huisgen 1,3-dipolar cycloaddition	**271**, **275**	*E. faecalis*
Scheme 54	Cu-catalyzed 1,3-dipolar cycloaddition	**284**	*Mycobacterium tuberculosis*, *B. subtilis*
Scheme 55	Cu-catalyzed 1,3-dipolar cycloaddition	**287**, **288**	Various gram-positive and gram-negative strains
Scheme 56	Cu-catalyzed azide-alkyne cycloaddition (CuAAC)	**293**, **295**	*E. coli*, other bacteria
Scheme 58	Cu-catalyzed oxidative cross-dehydrogenative coupling	**306a**, **306c**, **306d**	*Mycobacterium tuberculosis*
Scheme 59	CuI/oxone-catalyzed cyclization of thioureas	**310**	Various bacteria
Scheme 60	Cu-catalyzed 1,2-addition	**312a**, **312b**, **312c**	*E. coli*
Nickel-catalyzed synthesis			
Scheme 61	Ni-catalyzed Kumada cross-coupling	**315**	Gram-positive and gram-negative bacteria
Scheme 62	Kumada and Suzuki cross-coupling	**320**	Gram-positive and gram-negative bacteria
Scheme 63	Ni-catalyzed Buchwald-Hartwig reaction	**324**	Gram-positive and gram-negative bacteria
Scheme 64	Photocatalytic cross-coupling	**328**	*S. faecalis*, *B. subtilis*, *K. pneumonia*, *E. coli*, and *P. aeruginosa*

## Abbreviations

**Full Form**	**Abbreviation**
Global Antimicrobial Resistance and Use Surveillance System	GLASS
Antimicrobial resistance	AMR
Dimethylformamide dimethyl acetal	DMFDMA
Extended-spectrum β-lactamase	ESBL
Extensively drug-resistant	XDR
Dichloromethane	DCM
Thymidylate monophosphate kinase	TMPK
photoactive polyphenylene ethynylene	PPE
Minimum inhibitory concentrations	MIC
Bacterial leaf blight	BLB
Chan-Evans-Lam	CEL
Minimum Inhibitory Concentration	MIC
Cu-catalyzed 1,3-dipolar cycloaddition	CuAAC
